# Proteomics fingerprinting reveals importance of iron and oxidative stress in *Streptomyces scabies*–*Solanum tuberosum* interactions

**DOI:** 10.3389/fmicb.2024.1466927

**Published:** 2024-10-02

**Authors:** Lauriane Giroux, Iauhenia Isayenka, Sylvain Lerat, Nathalie Beaudoin, Carole Beaulieu

**Affiliations:** Département de Biologie, Centre SÈVE, Université de Sherbrooke, Sherbrooke, QC, Canada

**Keywords:** common scab, iron, proteomics, oxidative stress, potato, *Streptomyces scabies*

## Abstract

**Introduction:**

The Gram-positive actinobacterium *Streptomyces scabies* is the major causal agent of potato common scab. The main pathogenicity factor is thaxtomin A, a phytotoxin that causes atypical cell death, although other secondary metabolites have been described to play a role in *S. scabies* virulence. Despite this, many aspects of the interaction between *S. scabies* and its primary host *Solanum tuberosum* L. remain to be elucidated.

**Methods:**

Intracellular proteins of *S. scabies* EF-35 grown in the presence of *in vitro* produced tubers (microtubers) of the Russet Burbank and Yukon Gold potato cultivars were extracted and analysed by electrospray mass spectrometry (ES MS/MS). Based on the results of proteomic analysis, iron quantification by ICP-MS and nitrite quantification using Griess reagent in growth media as well as RT-qPCR analysis of the siderophore pyochelin gene expression were performed in the presence and absence of microtubers. Hydrogen peroxide accumulation was also determined in the nutrient medium used for co-cultivation of bacteria and potato microtubers.

**Results:**

Potato microtubers caused an increase in the content of bacterial proteins involved in stress and defense, secondary metabolism, and cell differentiation, as well as secreted proteins. Co-cultivation with potato microtubers induced the accumulation of *S. scabies* proteins implicated in siderophore pyochelin biosynthesis, nitrite production and oxidative stress perception and response. The increase in the abundance of proteins related to pyochelin biosynthesis was consistent with a significant decrease in the iron content in the culture medium, as well as with induction of expression of pyochelin biosynthesis genes. Elevated nitrite/sulfite reductase protein levels were associated with increased nitrite excretion by *S. scabies* cells in the presence of host microtubers. The increase in the levels of proteins associated with signaling and oxidative stress response could have been caused by the accumulation of ROS, in particular hydrogen peroxide, detected in the studied system.

**Discussion:**

These findings show that interactions of *S. scabies* with living potato microtubers induce the production of secondary metabolites, defense responses, and protection from oxidative stress. This study suggests the importance of iron during host - *S. scabies* interactions, resulting in competition between pathogen and its host.

## 1 Introduction

Potato common scab is a disease causing esthetic defects on potato tubers characterized by suberized lesions appearing on their surface. These lesions may be superficial, raised or pitted. Even though the lesions mainly affect the appearance of tubers, the disease causes significant economic losses for the producers (Hill and Lazarovits, 2005). One of the main causal agents of potato common scab in North America (Canada) is *Streptomyces scabies.* However, other *Streptomyces* species were also reported to cause similar symptoms (Goyer et al., 1996; Wanner, 2009; Hudec et al., 2021).

*S. scabies* is a sporulating Gram-positive actinobacterium inhabiting soil that infects young tubers during their development. Its pathogenicity is linked to the production of phytotoxins called thaxtomins (Loria et al., 2008). Thaxtomins are cyclic dipeptides derived from 4-nitrotryptophan and phenylalanine that inhibit the biosynthesis of cellulose (King and Calhoun, 2009). Thaxtomin A, the main form of toxin produced by *S. scabies*, also induces a programmed cell death in plants that is not a typical hypersensitive reaction, since defense responses generally associated with the hypersensitive response, such as oxidative burst, were not activated in the presence of the toxin (Duval et al., 2005). Although pathogenicity is dependent on thaxtomin A production (Healy et al., 2000), *S. scabies* synthesizes other toxins as well as proteins that may participate in pathogenicity (Liu et al., 2021). This plant pathogen produces coronafacoyl toxins, a group of toxins that enhance disease symptom severity produced by a large number of plant pathogenic bacteria (Fyans et al., 2015; Bignell et al., 2018; Cheng et al., 2019), concanamycins, inhibitors of V-ATPase (Natsume et al., 1998) and scabin, a mono-ADP-ribosyltransferase toxin (Lyons et al., 2016). It also produces a necrotic factor, the Nec1 protein (Joshi M. V. et al., 2007; Joshi M. et al., 2007), a tomatinase that degrades plant glycoalkaoloids (Seipke and Loria, 2008) and various hydrolytic enzymes including the suberinase Sub1 (Jabloune et al., 2020; Komeil et al., 2014). Among these factors, thaxtomins and concanamycins were shown to be produced *in planta* (King et al., 1989; Natsume et al., 2017).

Proteome and secretome of *S. scabies* have been characterized especially during its growth on plant cell wall constituents such as suberin (Lauzier et al., 2008; Komeil et al., 2014; Beaulieu et al., 2016), cellulose (Padilla-Reynaud et al., 2015) or cellobiose disaccharide, the main product of cellulose degradation (Planckaert et al., 2018). The interest of analyzing the protein profile of *S. scabies* in the presence of suberin or cellobiose relies on the fact that these plant constituents were shown to induce thaxtomin biosynthesis (Lerat et al., 2010). Although these previous studies shed a light on some aspects of *S. scabies* pathogenicity, the proteomic response of *S. scabies* to the presence of living potato tubers has not yet been investigated.

This pathogen was shown to infect young tubers through stomata, lenticels, or wounds, but the thickness and texture of the tuber periderm also plays a role in the development of infection (Loria et al., 1997; Khatri et al., 2011; Beaudoin et al., 2021). Studies of the extracellular and intracellular *S. scabies* proteome have shown that the pathogen reacts to the presence of potato tuber suberin by changing its physiological state, switching to secondary metabolism (Lauzier et al., 2008; Komeil et al., 2014; Beaulieu et al., 2016; Planckaert et al., 2018). In the presence of suberin, *S. scabies* produced extracellular proteins such as lipolytic enzymes and glycosyl hydrolases that allowed the degradation of this recalcitrant polymer affecting the aliphatic domain and sugar content of suberin (Komeil et al., 2014; Beaulieu et al., 2016; Khalil et al., 2019). Further research has shown that the presence of cellobiose, which induces *S. scabies* virulent behavior, triggers a range of bacterial processes such as toxin production, starch utilization, and siderophore synthesis (Planckaert et al., 2018).

*In vitro* production of potato tubers has been used for many years in the study of potato metabolism. For instance, *in vitro*-produced microtubers have provided a model system to study potato tuberization, potato dormancy and stress metabolism (Coleman et al., 2001; Shan et al., 2013; Chourasia et al., 2021). Microtubers have also been used to test virulence of plant pathogens including *S. scabies* (Lawrence and Barker, 1963). In the present study, we use developing *in vitro* grown potato tubers to mimic a more complete system of pathogen-host interactions. *S. scabies* was grown in a liquid culture medium supplemented or not with living potato microtubers. The effect of the presence of potato tubers on *S. scabies* proteome has been analyzed.

## 2 Materials and methods

### 2.1 Bacterial growth conditions

*Streptomyces scabies* strain EF-35 (Faucher et al., 1992) was pre-cultured with agitation at 30°C for 3 days in YME (yeast malt extract medium: 4 g/L glucose, 4 g/L yeast extract, and 10 g/L malt extract). Bacteria were then recovered by centrifugation for 10 min at 3450 *g* and supernatant was discarded. Bacteria were washed with saline (*ca.* 10 mL of 0.85% NaCl) twice and pelleted again by centrifugation. The bacterial inoculum was prepared by resuspending the pellet in 5 volumes (based on pellet volume) of saline. The obtained bacterial suspension (150 μL, unless otherwise stated) was used to inoculate 10 mL of Murashige-Skoog medium (Murashige and Skoog, 1962) supplemented with 0.05% (w/v) starch (MS-S medium). Five *in vitro*-grown microtubers (see below) were added to the culture medium when needed. In all experiments, unless otherwise stated, cultures with or without microtubers were incubated for 5 days at 30°C with agitation (125 rpm).

### 2.2 Plant material and growth conditions

Potato cultivars Russet Burbank (RB; moderately resistant to common scab) and Yukon Gold (YG; susceptible to common scab) were used in this study. *In vitro*-potato plantlets were maintained and propagated on MS medium (Murashige and Skoog Basal Salt Mixture, M5524, Sigma), pH 5.7, supplemented with 3% (w/v) sucrose and 0.7% (w/v) agar. Light conditions were set to 60–75 μmol/s/m^2^ with a 16/8-h light/dark period and a constant temperature of 22°C in a Sanyo MLR-350 plant growth cabinet. Aseptically grown microtubers were produced using one-node stem segments propagated on MS supplemented with 8% (w/v) sucrose, 0.7% (w/v) agar (pH 5.7) and kept in the dark for 6 weeks at 22°C/18°C 16 h/8 h period (Sadek Hossain et al., 2017). After 6 weeks, microtubers of 3 to 7 mm diameter were harvested and instantly used for experiments.

### 2.3 Intracellular soluble protein extraction

After removal of potato microtubers, bacterial cultures grown in the presence of microtubers, as well as control cultures grown in the absence of microtubers, were recovered by centrifugation at 3,450 g for 10 min. The bacterial pellet was washed with cold phosphate buffered saline (PBS) and centrifuged at 3,450 g for 5 min. The supernatant was discarded, and the pellet resuspended in 0.6 mL of PBS. The samples were subjected to two cycles of freezing and thawing which consisted of 20 min in ethanol at −80°C followed by 10 min at room temperature. After freeze/thaw cycles, the samples were vortexed for 10 s and sonicated on ice for 20 s. The samples were centrifuged for 10 min at 16,000 g and the supernatants were collected. EDTA (0.3 mM final concentration) was added to the supernatant to avoid protein degradation.

### 2.4 Proteomic analysis

Fifteen μL of the isolated intracellular proteins were migrated in sodium dodecyl sulfate-polyacrylamide gel (10% w/v SDS-PAGE) for 30 min at 70 V. The band containing proteins was excised from the gel. In-gel digestion followed by mass spectrometry were performed at the Proteomics Platform of the Centre hospitalier universitaire de Québec Research Center (Quebec City, QC, Canada).

Briefly, proteins were reduced with 10 mM DTT and alkylated with 55 mM iodoacetamide. Trypsin digestion was performed using 126 nM of modified porcine trypsin (sequencing grade, Promega, Madison, WI, United States) at 37°C for 18 h. Digestion products were extracted using 1% formic acid and 2% acetonitrile followed by 1% formic acid and 50% acetonitrile. The recovered extracts were pooled, vacuum centrifuge-dried and resuspended in 0.1% formic acid.

Peptide samples were separated by online reversed phase (RP) nanoscale capillary liquid chromatography (nanoLC) and analyzed by electrospray mass spectrometry (ES MS/MS). The experiments were performed with an Ekspert NanoLC425 (Eksigent) coupled to a 5,600+ mass spectrometer (Sciex, Framingham, MA, United States) equipped with a nanoelectrospray ion source. Peptide separation took place on a self-packed picofrit column (New Objective) with Reprosil 3 μm 120 Å C18, 15 cm × 0.075 mm internal diameter. Peptides were eluted with a linear gradient from 5 to 35% of acetonitrile with 0.1% formic acid in 35 min at 300 nL/min. Mass spectra were acquired using a data dependent acquisition mode using Analyst software version 1.7. Each full scan mass spectrum (400–1,250 m/z) was followed by collision-induced dissociation of the 20 most intense ions. Dynamic exclusion was set for a period of 12 s and tolerance of 100 ppm.

Mascot generic format (MGF) peak list files were created using Protein Pilot version 4.5 software (Sciex). MGF sample files were then analyzed using Mascot (Matrix Science, London, UK; version 2.5.1). Mascot was set up to search the Uniprot TAX_Streptomyces_database (702,270 entries) assuming the digestion enzyme trypsin. Mascot was searched with a fragment ion mass tolerance of 0.100 Da and a parent ion tolerance of 0.100 Da. Carbamidomethyl of cysteine was specified in Mascot as a fixed modification. Deamidation of asparagine and glutamine and oxidation of methionine were specified in Mascot as variable modifications.

Scaffold (version Scaffold_4.8.4, Proteome Software Inc., Portland, OR, United States) was used to validate MS/MS based peptide and protein identifications. Peptide identifications were accepted if false discovery rate (FDR) was at 1.0% by the Scaffold Local FDR algorithm or if relevant FDR was less than 1.0% and contained at least 2 identified peptides. Protein probabilities were assigned by the Protein Prophet algorithm (Nesvizhskii et al., 2003). Proteins that contained similar peptides and could not be differentiated based on MS/MS analysis alone were grouped to satisfy the principles of parsimony. This experiment was done on four biological replicates.

Identified proteins were assigned to a protein function group using the UniProt, KEGG and eggNOG databases. The normalized spectral count (NSpC) was defined as a spectral count of a protein divided by its molecular weight and the normalized spectral abundance factor (NSAF) was a ratio between NSpC of a protein and the total NSpC.

### 2.5 Nitrite, iron and thaxtomin A quantification

Concentration of nitrite in culture supernatants was determined using the Griess test (Green et al., 1982) after 5 days of incubation. Culture supernatant (300 μL) was mixed with 100 μL of Griess reagent (0.1% N-(1-naphthyl)ethylenediamine dihydrochloride, 1% sulfanilamide and 5% phosphoric acid) and 600 μL of water. After incubation at room temperature for 10 min, absorbance was measured with a spectrophotometer at 540 nm. Nitrite concentration was determined using a sodium nitrite standard curve. Uninoculated MS-S medium was used as the reference blank. The experiment was carried out in three biological replicates of *S. scabies* grown with or without microtubers.

The quantification of iron content in growth medium was carried out using 500 μL of culture supernatant from *S. scabies* grown with or without microtuber exudates (see below) or grown in the presence of microtubers. Samples were digested with HNO_3_ (2% final concentration) for 2 h at 65°C. Iron content was determined by ICP-MS (inductively coupled plasma mass spectrometry) using a X-Series II ICP-MS (ThermoFisher Scientific, Waltham, MA, United States). Microtuber exudates were obtained by removing microtubers from MS-S medium after 5 days of cultivation. This experiment was performed in three biological replicates.

Thaxtomin A was extracted from 10 mL of bacteria-free culture supernatant, purified and quantified as previously described (Lerat et al., 2010). This experiment was performed in three biological replicates.

### 2.6 Reverse transcription quantitative real-time PCR

The expression of *txtD*, the gene encoding for a nitric oxide synthase involved in thaxtomin biosynthesis (SCAB_31841) was compared between cultures in the presence or absence of potato microtubers. Bacteria were collected from 10 mL of a 5 days-old culture medium by centrifugation for 10 min at 3,450 g. RNA extraction, reverse transcription and reverse transcription quantitative real-time PCR (qRT-PCR) were carried out according to Khalil et al. (2019). Relative expression levels were determined by using the comparative CT values according to Pfaffl (2001) with *gyrA* (gyrase A) as a reference gene. Primers were designed specifically for the experiment using PrimerQuest tool (Integrated DNA Technologies, Inc.). Primer sequences for the expression analysis of *gyrA* and *txtD* genes were previously reported in Lerat et al. (2010). Sequences of the primers used in this assay are listed in Supplementary Table S1. The experiment was carried out in three biological replicates.

Expression of three genes located in *S. scabies* pyochelin gene cluster (SCAB_1381, SCAB_1391, and SCAB_1481) was tested in standard MS-S as well as in a MS-S medium deprived of iron (see below) in the presence or not of YG cultivar microtubers. To compare conditions with and without iron, the MS medium (M5524, Sigma) used in the experiments was prepared manually according to the list of solutes provided by the manufacturer. The ferrous sulfate solution (FeSO_4_) was prepared separately, sterilized by filtration, and added, if necessary, to the prepared MS media to a final concentration of 100 μM. Bacteria were cultivated in these media in the presence or absence of microtubers at 30°C, 125 rpm for 48 h. Bacterial cells were harvested by centrifugation, total RNA was isolated, and transcriptional levels were analyzed as described above. The experiment was carried out in at least three biological replicates.

The effect of oxidative stress on the expression of SCAB_1381, SCAB_1391, and SCAB_1481 was tested as follows. Bacterial inoculum (300 μL) was added to 20 mL of MS-S medium. Oxidative stress was induced by 1 mM sodium nitroprusside or 50 μM of H_2_O_2_, added to the MS-S medium with full iron content (Fe 100 μM) or during iron deprivation (Fe 0 μM) after 24 h of *S. scabies* EF-35 cultivation. Oxygen stress was applied for additional 24 h, after which bacterial cells were harvested, RNA isolated, and transcriptional level of the pyochelin biosynthesis genes was analyzed. The experiment was carried out in four biological replicates.

### 2.7 Determination of peroxide accumulation in the culture medium

Bacterial cells *S. scabies* EF-35 were prepared and incubated in MS supplemented with 0.05% starch in the presence or absence of microtubers as described above. After 3, 5 and 7 days, 500 μL of culture medium was collected from three (for MS-S medium only) or four biological samples and centrifuged at 8,000 g for 5 min to remove debris. The supernatant was transferred to new tubes and analyzed using Peroxide Quantitative Assay Kit (Water-compatible) (BSP069 BioBasic) according to manufacturer’s instruction. Samples of 20 μL were mixed with 200 μL of working reagent consisting of 1 volume of Solution A and 100 volumes of Solution B in microplate wells. Assay reactions were mixed and incubated for 20 min at room temperature. After incubation, optical density was measured at 560 nm. To avoid endogenous transition metal interference, the absorbance of the blank omitting reagent A was subtracted from the absorbance values obtained. The amount of peroxide was calculated from a standard curve generated by diluting a 3% peroxide solution to standard concentrations from 0 to 80 μM.

## 3 Results

### 3.1 Potato microtubers induce changes in the intracellular proteome of *Streptomyces scabies*

The intracellular proteome of *S. scabies* EF-35 grown in the presence of potato microtubers of Russet Burbank (RB) and Yukon Gold cultivars (YG) was characterized in comparison with a bacterial culture cultivated in the absence of plant material. A total of 1,777 proteins were identified during the analysis (data available at Borealis V1 database, Isayenka, 2024). A total of 876 proteins presenting a mean of at least three spectral counts in at least one of the culture conditions were retained for further analysis and were assigned to a protein functional group (Supplementary Table S2).

**Figure 1:**
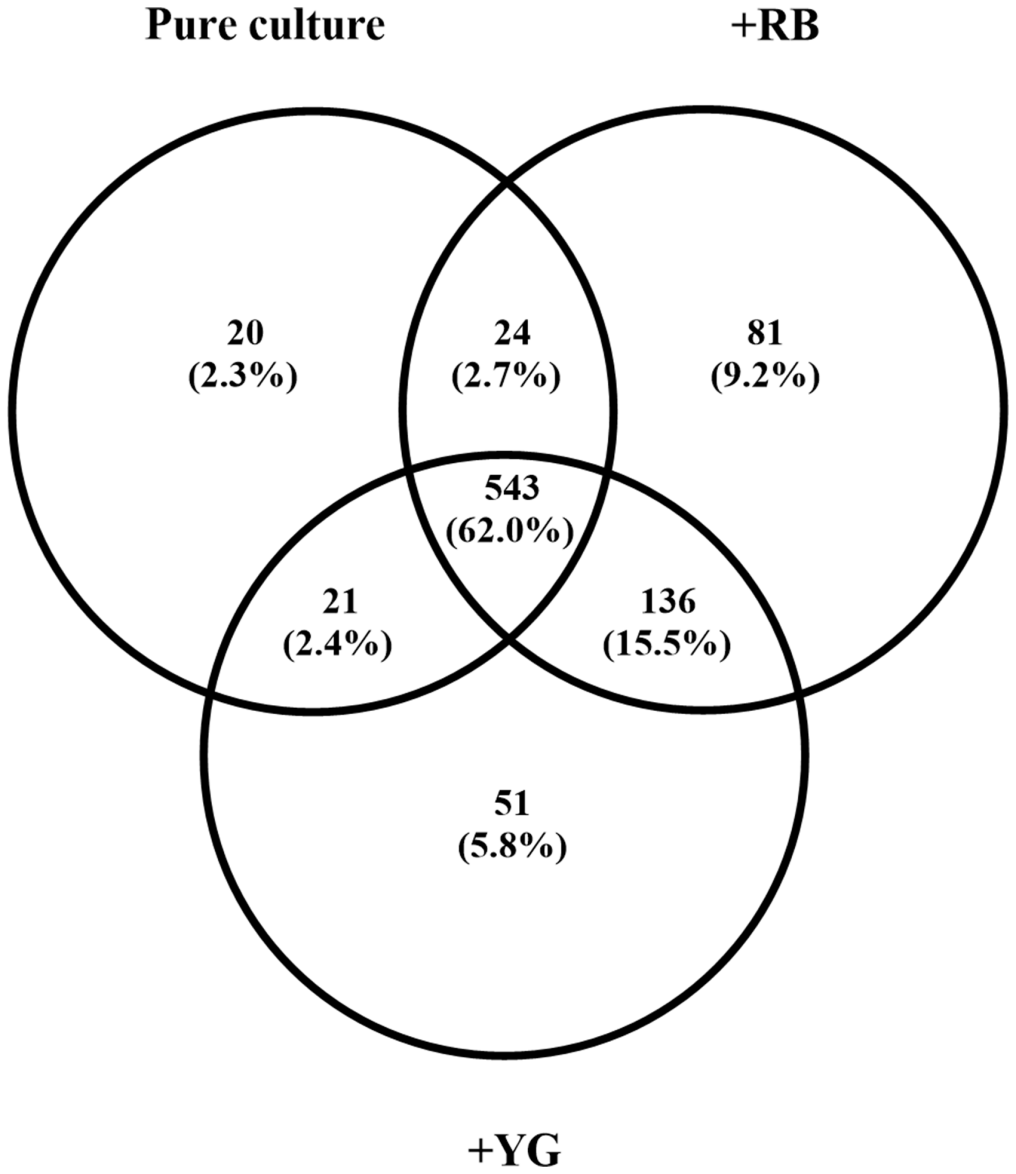
Number of *Streptomyces scabies* proteins produced after 5 days in pure culture or when cultivated in the presence of Russet Burbank (RB) or Yukon Gold (YG) microtubers. Only proteins associated with an average of three spectral counts were retained. The analysis was performed in four biological replicates.

Five hundred eighty-eight (about 70%) of these proteins were produced independently of the presence of potato microtubers (Figure 1). A total of 268 proteins were detected only in the presence of microtubers and 136 (51%) of these proteins were detected in the presence of both cultivars (Figure 1).

Comparison of *S. scabies* proteome in pure culture and those during interaction with potato microtubers revealed changes of over 10% in the presence of both potato cultivars in several functional groups (Table 1). The percentage of stress and defense related proteins increased by 38% and by 20% in the presence of RB and YG, respectively. The contribution of proteins involved in secondary metabolism and differentiation increased by 43% in the presence of RB and 39% in the presence of YG cultivar. The content of proteins involved in secretion increased by 21% with the addition of RB and 17% with the addition of YG tubers compared to a pure bacterial culture.

**Table 1:** Distribution of *Streptomyces scabies* EF-35 proteins within functional groups depending on culture conditions*.

Protein functional group	Normalized spectral abundance factor (%)^†^		
	Pure culture	EF-35 + RB	EF-35 + YG
Amino acid metabolism	10.76	9.52	9.81
Carbohydrate metabolism	12.81	12.15	13.31
Cell cycle control, cell division, chromosome partitioning	0.56	0.46	0.39
Cell wall/membrane/envelope biogenesis	2.08	2.09	2.19
Coenzyme transport and metabolism	1.55	1.37	1.40
Energy production and conversion	13.49	14.1	13.49
Inorganic ion metabolism	2.15	1.66	1.98
Lipid metabolism and ketogenesis	4.36	4.52	4.81
Nucleotide metabolism	2.30	2.35	2.26
Post-translational modification, protein turnover, and proteolysis	4.06	4.33	4.23
Replication, recombination and DNA repair	1.47	1.30	1.32
Transcription and RNA processing	6.88	6.09	6.08
Translation, ribosomal structure and biogenesis	14.25	13.25	13.00
Secondary metabolism and differentiation	1.58	2.26	2.20
Stress and defense mechanism	7.50	10.38	9.02
Secretion	0.47	0.57	0.55
General function predicted only	5.41	4.70	4.77
Unknown function	8.31	8.90	9.17

^*^S. scabies was grown in MS-S medium supplemented or not with potato microtubers cv. Russet Burbank (RB) or Yukon Gold (YG). Proteomic analysis was performed after 5 days of cultivation.

^†^Data are the mean of four biological replicates.

In contrast, a decrease in the representation of proteins involved in control of the cell cycle, cell division, chromosome partitioning, transcription and RNA processing, DNA replication, recombination and repair was observed. The decrease also occurred in the group of proteins with predicted general function (Table 1).

Proteins with an NSAF value at least 2-fold higher or 2-fold lower in the presence of both cultivars than in pure culture are listed in Supplementary Tables S3, S4, respectively. Six proteins assigned to the functional group of secondary metabolism and differentiation were at least twice overproduced in the presence of microtubers of both cultivars.

Of the nine proteins assigned to the functional group of stress and defense mechanism, seven appeared to be linked to oxidative stress perception (C9ZEV0 and C9YVQ3) and adaptation (C9YY94, C9YY95, C9ZEV0, C9YZM9, C9YU91) (Supplementary Table S3).

For the functional group including inorganic ion metabolism proteins, protein content decreased by 23% in the presence of RB tubers and by 8% in the presence of YG tubers (Table 1). However, only the upregulated proteins had at least a 2-fold NSAF variation (Supplementary Table S3). These included proteins C9ZCX9 and C9ZCY1 which are involved in pyochelin biosynthesis (Seipke et al., 2011) and nitrite/sulfite reductase (C9YUJ7). The C9ZCX9 normalized spectral abundance factors of 0.034 and 0.56% were determined in samples with YG and RB, respectively, while no peptides were detected in the absence of microtubers. The presence of microtubers increased NSAF of the C9ZCY1 protein from 0.006% in the absence of microtubers to 0.026% (RB) and 0.021% (YG). NSAF of nitrite/sulfite reductase (C9YUJ7) increased from 0.014% in the absence of potato microtubers up to 0.031 and 0.030% in the presence of RB and YG microtubers, respectively.

In the carbohydrate metabolism functional group, 24 proteins were found to be overproduced in the presence of both cultivars with at least a 2-fold NSAF variation (Supplementary Table S3). Upregulated proteins in this large functional group exhibit hydrolase (C9ZBJ5, A0A081XI56, A0A1Q5LYT5), oxidoreductase (C9YT49, C9Z4B0), isomerase (C9ZFY5, C9Z1V1, C9ZD79) and transferase (C9Z9C4) enzymatic activities, which are possibly related to the breakdown and interconversion of carbohydrates. Other overproduced proteins are directly involved in carbohydrate degradation (A0A101PMD4, A0A0M8WDG6). Beta-glucosidase (C9YXN3), Levansucrase (C9YSZ3) and Glucose-1-phosphate adenylyltransferase (C9Z540) are implicated in starch and sucrose metabolism. Several proteins are implicated in the Krebs cycle (TCA cycle) (A0A170XU30), glycolysis and pyruvate metabolism (C9YXR3 and C9ZGR4). Another seven putative extracellular substrate-binding proteins belong to the sugar (C9ZD81, C9Z1U7, C9YYL7), monosaccharide (C9ZDX0), or carbohydrate (C9ZAA7, C9Z619, A0A0L8L2H4) transport system of *S. scabies*. Another protein, the putative rhamnosidase (C9YXN7) exhibiting hydrolase activity could be implicated in degradation of plant flavonoids—rutin and naringin. Downregulated proteins belonging to the carbohydrate metabolism functional group are involved in glycolysis (C9YUA4, A0A089X9G2), associated with the pentose phosphate pathway (C9YSV0) or catalyzed the hydrolysis of glycosidic bonds (C9Z737). A decrease in the content of two DNA-binding proteins related to ABC transporters (A0A0U3Q3H2, L1KT66) and maltodextrin-binding transport protein MdxE was also detected (A0A1D2IGZ0, Supplementary Table S4).

The NSAF value of peptides involved in metabolism of alanine, aspartate and glutamate increased at least by 2-fold in the presence of potato tubers (C9ZGG2, C9Z7K3, C9Z402, and A0A143C079, Supplementary Table S3). A decrease in the abundance of three proteins involved in L-lysine biosynthesis was detected in the presence of host tubers (C9YYA5, C9ZBW7, C9ZA98, Supplementary Table S4). The content of two proteins involved in the transport of amino acids and peptides via ABC transporters was also significantly reduced in the presence of potato tubers (C9YWN6 and C9Z5D2).

The functional group of energy metabolism included diverse proteins predominantly characterized by dehydrogenase and oxidoreductase activities (Supplementary Table S3). In the functional group related to lipid metabolism, it was found that the content of a multifunctional oxidative enzyme (C9Z865) involved in the breakdown of fatty acids increased in the presence of potato tubers (Supplementary Table S3). At the same time presence of host tubers reduced concentration of long-chain-fatty-acid-CoA ligase, involved in lipid biosynthesis (C9ZGL8, Supplementary Table S4).

### 3.2 *Streptomyces scabies* excretes nitrite in the presence of microtubers

A 2-fold increase in the relative abundance of nitrite/sulfite reductase protein (C9YUJ7, Supplementary Table S3) in the presence of microtubers could indicate the induction of nitrite production. Nitrite was detected in growth medium (after 5 days incubation) when *S. scabies* EF-35 was grown in the presence of both RB (159.0 ± 4.3 μM) and YG (140.4 ± 59.4 μM) cultivars. However, no nitrite was detected in control conditions when bacterial cells were grown without microtubers or when the microtubers were incubated in the absence of bacterial cells.

### 3.3 Co-cultivation alters culture medium iron content

**Figure 2:**
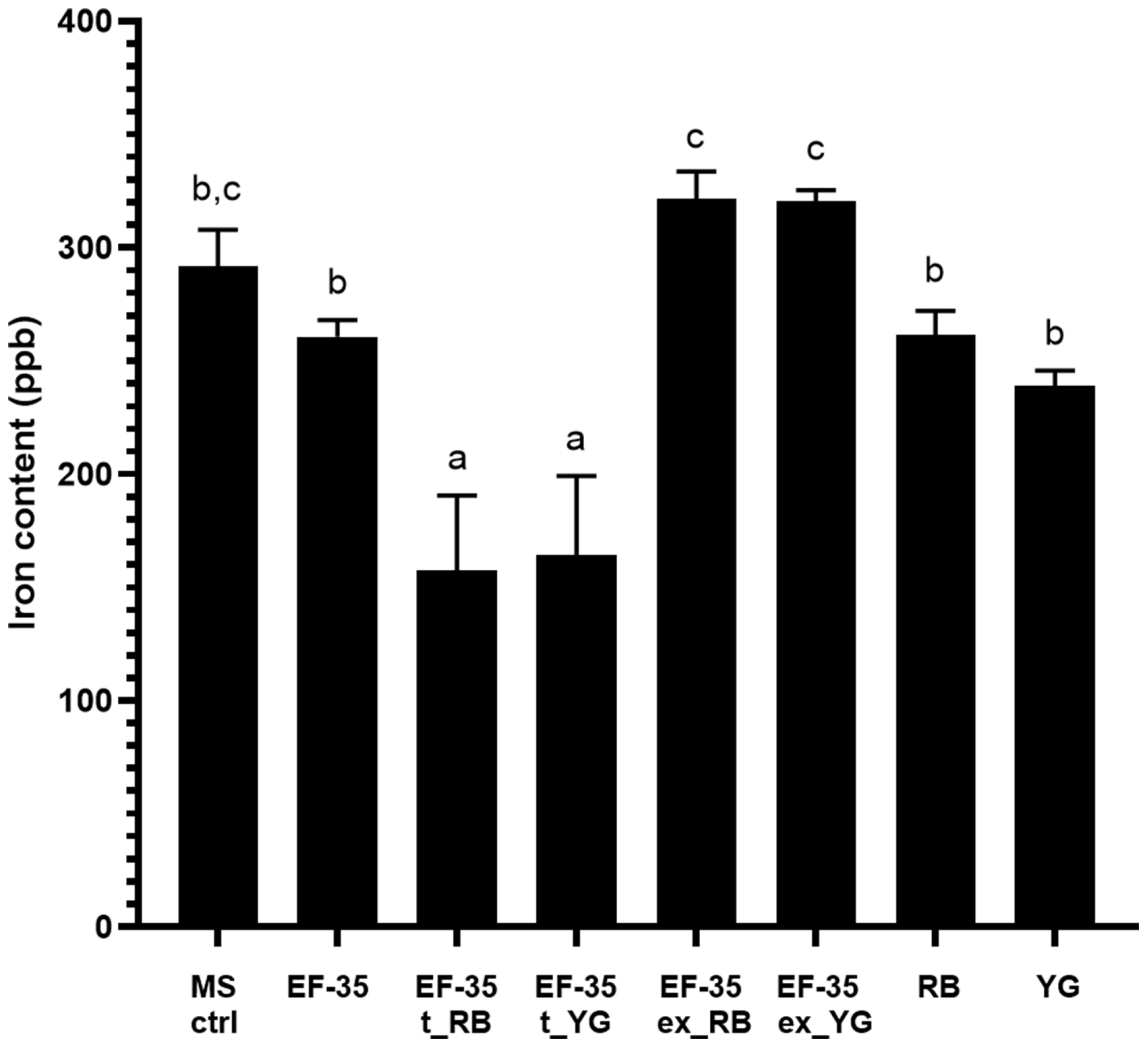
Presence of microtubers reduces iron content in culture medium after 5 days. Uninoculated MS-S culture medium (MS ctrl). *Streptomyces scabies* EF-35 MS-S culture medium without microtubers (EF-35), with Russet Burbank microtubers (EF-35 t_RB), with Yukon Gold microtubers (EF-35 t_YG). *S. scabies* MS-S culture medium containing exudates of Russet Burbank (EF-35 ex_RB) or Yukon Gold (EF-35 ex_YG) microtubers. MS-S culture medium with Russet Burbank (RB) or Yukon Gold microtubers (YG). The values are the mean of three biological replicates (± standard error of the mean). Data with the same letter are not significantly different (*p* < 0.05, one-way ANOVA followed by Fisher’s LSD test). ppb, parts per billion.

Since the current study identified some overproduced proteins associated with iron acquisition and metabolism, ICP-MS analysis was performed to quantify the amount of iron in the culture medium (MS-S) used to maintain *S. scabies* in the presence or absence of host microtubers. Results indicate that the control medium lacking biological material, contained 291.7 ± 16.3 ppb of iron. Separate cultivation of bacteria or microtubers in MS-S medium did not significantly change iron content (Figure 2). Cultivation of the bacteria for 5 days resulted in a slight not significant decrease in the iron content to 260.7 ± 7.3 ppb in MS-S medium compared to the non-inoculated medium. Similarly, incubation of potato tubers for 5 days in the MS-S medium did not significantly decrease the amount of iron: 261.3 ± 10.7 ppb for RB and 239.0 ± 6.7 ppb for YG microtubers compared to the control non-inoculated MS-S. However, when *S. scabies* was grown in the presence of RB or YG microtubers, the amount of iron in MS-S decreased significantly to 157.3 ± 33.3 and 164.0 ± 35.2 ppb, respectively, compared to conditions when the bacterium grew in the absence of plant material, or when the microtubers were cultivated in the absence of bacteria. To determine if higher iron intake was due to interactions between host plant and bacteria, potato microtubers were incubated for 5 days in MS-S medium for exudate production, after which they were removed from the medium, which was subsequently inoculated with *S. scabies* EF-35 for another 5 days. After the cultivation of *S. scabies*, the amount of iron in supernatants containing tuber exudates was significantly higher (321.7 ± 11.9 for RB exudate and 320.3 ± 5.0 for YG) than after bacterial growth in MS-S medium without potato tubers or after cultivation of tubers alone (Figure 2).

### 3.4 Iron deprivation and presence of microtubers affect pyochelin biosynthesis gene expression

**Figure 3:**
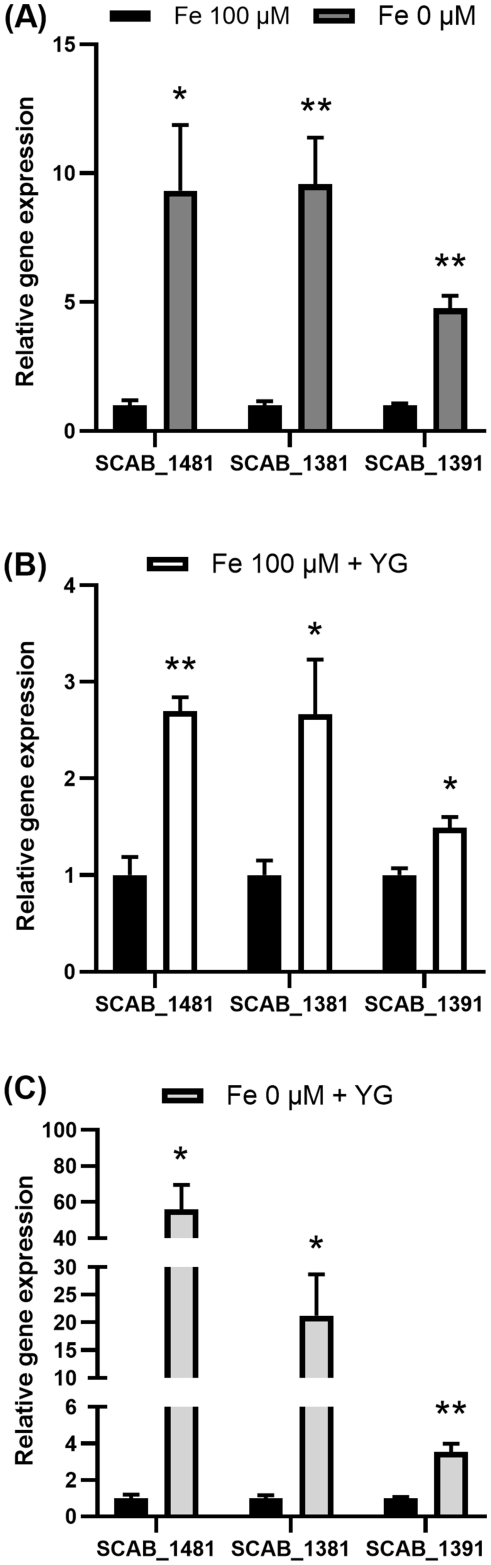
Transcription levels of pyochelin biosynthesis genes in *Streptomyces scabies* after 48 h incubation in MS-S medium with 0 μM or 100 μM Fe^2+^. Expression of SCAB_1481, SCAB_1381 and SCAB_1391 measured during iron deprivation (Fe^2+^ 0 μM) **(A)**, in the presence of host tubers of Yukon Gold (YG) **(B)**, in the absence of iron with the presence of potato tubers (Fe^2+^ 0 μM + YG) **(C)** in comparison to MS-S medium containing 100 μM of iron (Fe^2+^ 100 μM) as a control. Relative expression levels were determined using *gyrA* as a reference gene. The values are the mean of three biological replications (±standard error of the mean). Data analysis according to unpaired *t*-tests; *p values < 0.05 and **p values < 0.01. SCAB_1481: L-cysteine-[L-cysteinyl-carrier protein] ligase PchE, SCAB_1381: putative salicylate synthase and SCAB_1391: DUF885 domain-containing protein.

Proteomic analysis showed that *S. scabies* produced proteins C9ZCX9 (SCAB_1391) and C9ZCY1 (SCAB_1411) in the presence of potato microtubers. These proteins are responsible for the synthesis of the siderophore pyochelin, since the corresponding genes, which belong to the pyochelin biosynthesis gene cluster, were previously described in *S. scabies* (Seipke et al., 2011). The expression of genes located in pyochelin cluster (SCAB_1391, SCAB_1481, and SCAB_1381) was thus analyzed through qRT-PCR (Figure 3). The MS-S medium used in this study contained 100 µM or 0 µM of iron (Fe^2+^). As expected, when *S. scabies* was grown in MS-S deprived of iron, the transcriptional level of SCAB_1481 increased 9.3 ± 2.5-fold, 9.6 ± 1.8 for SCAB_1381 and 4.8 ± 0.5 for SCAB_1391 relative to their expression in the MS-S with a complete iron content (Fe^2+^ 100 μM) (Figure 3A). The presence of microtubers in the MS-S medium (Fe^2+^ 100 μM) caused a significant increase in the expression of SCAB_1481 by 2.7 ± 0.1-fold, SCAB_1381 by 2.7 ± 0.6-fold and SCAB_1391 by 1.5 ± 0.1-fold compared to the medium with 100 μM of Fe^2+^ which did not contain plant material (Figure 3B). The combined effect of the iron deprivation in the MS-S medium with the presence of potato microtubers caused a 56.0 ± 13.6-fold, 21.2 ± 7.4-fold and 3.5 ± 0.4-fold increase in the expression of SCAB_1481, SCAB_1381 and SCAB_1391, respectively, compared to the transcriptional level in the medium containing 100 μM of Fe^2+^ without host tubers (Figure 3C).

### 3.5 *Streptomyces scabies* secretes thaxtomin a in the presence of microtubers

When *S. scabies* EF-35 was grown in MS-S, only traces of thaxtomin A were detected in the culture medium (0.23 ± 0.12 μg). The production of thaxtomin A was up to 20-fold higher when the bacteria were exposed to RB microtubers (4.99 ± 4.14 μg) and 100-fold higher in the presence of YG microtubers (23.43 ± 7.93 μg). SCAB_31841 encoding TxtD enzyme was significantly overexpressed in 5 days-old culture medium in the presence of both RB and YG microtubers with a 2.98 ± 0.20 and 2.68 ± 0.25-fold change, respectively.

### 3.6 Co-cultivation of *Streptomyces scabies* and potato microtubers causes peroxide accumulation in the culture medium

**Figure 4:**
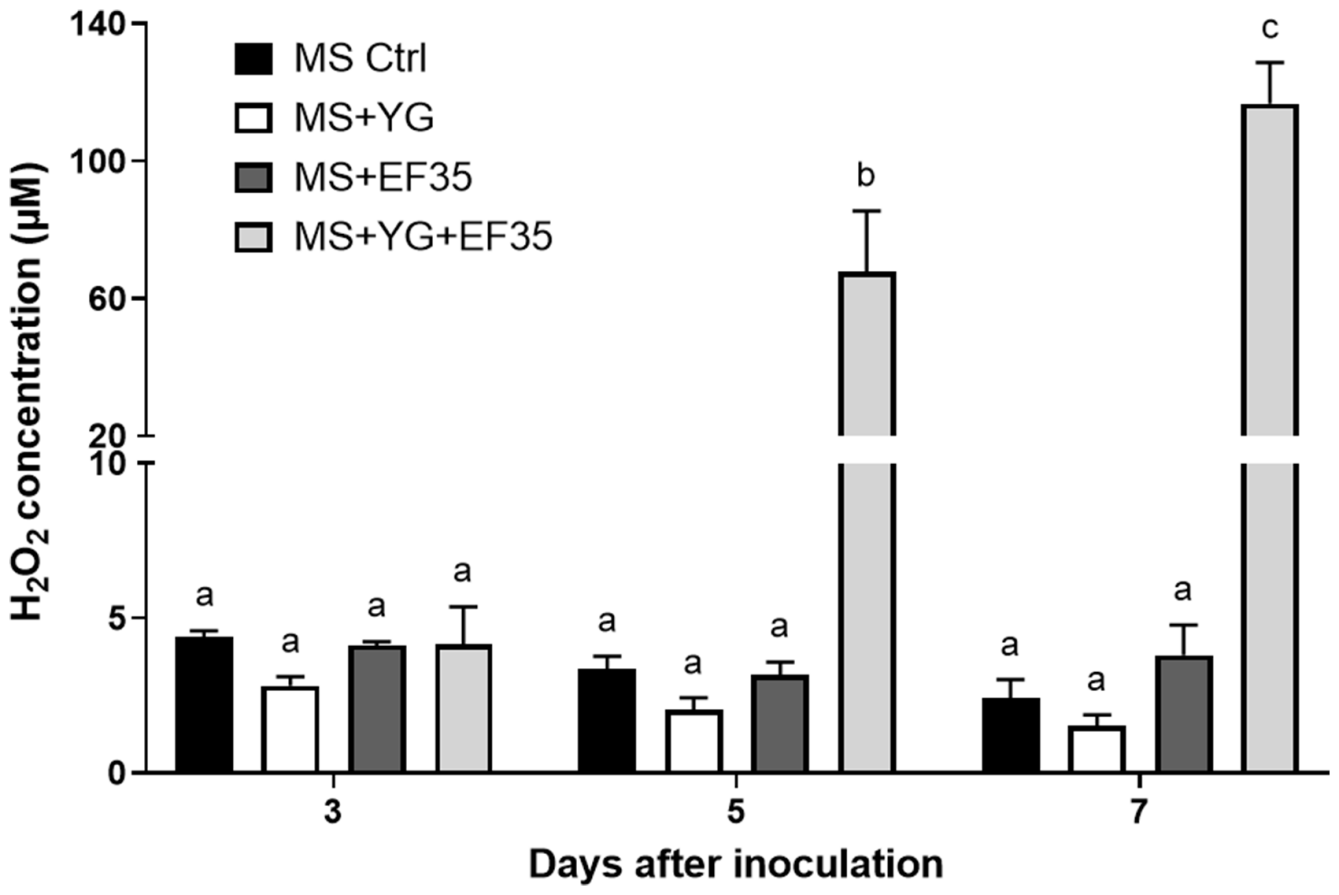
Peroxide content in the culture medium containing *Streptomyces scabies* EF-35 and/or microtubers of potato cv. Yukon Gold (YG) compared to the medium devoid of plant material and bacterial cells. MS Ctrl: MS-S medium without biological material; MS + YG: MS-S medium containing microtubers of cv. Yukon Gold; MS + EF-35: MS-S medium inoculated with *S. scabies* EF-35; MS + YG + EF-35: containing both microtubers and bacterial cells. The values are the mean of three biological replicates for MS Ctrl and four biological replicates for all other conditions (±standard error of the mean). Data with the same letter are not significantly different (*p* < 0.05, two-way ANOVA followed by Fisher’s LSD test).

The amount of peroxide present in a MS-S medium where *S. scabies* EF-35 or potato microtubers were cultivated separately was estimated in a time-dependent manner. The concentration of peroxide in the medium containing no biological material ranged from 4.4 ± 0.2 (day 3) to 2.4 ± 0.6 μM (day 7) (Figure 4). The presence of *S. scabies* EF-35 or potato tubers in MS-S medium did not significantly affect the level of peroxide. Co-cultivation of bacterial cells with YG microtubers significantly increased peroxide level starting from the 5th day of co-cultivation (67.8 ± 17.6 μM at 5th and 116.7 ± 11.9 μM at 7th day) (Figure 4). Similar results were obtained with the microtubers of RB cultivar when they were co-cultivated with *S. scabies* EF-35 (Supplementary Figure S1).

### 3.7 Oxidative stress does not induce the expression of pyochelin biosynthesis genes

**Figure 5:**
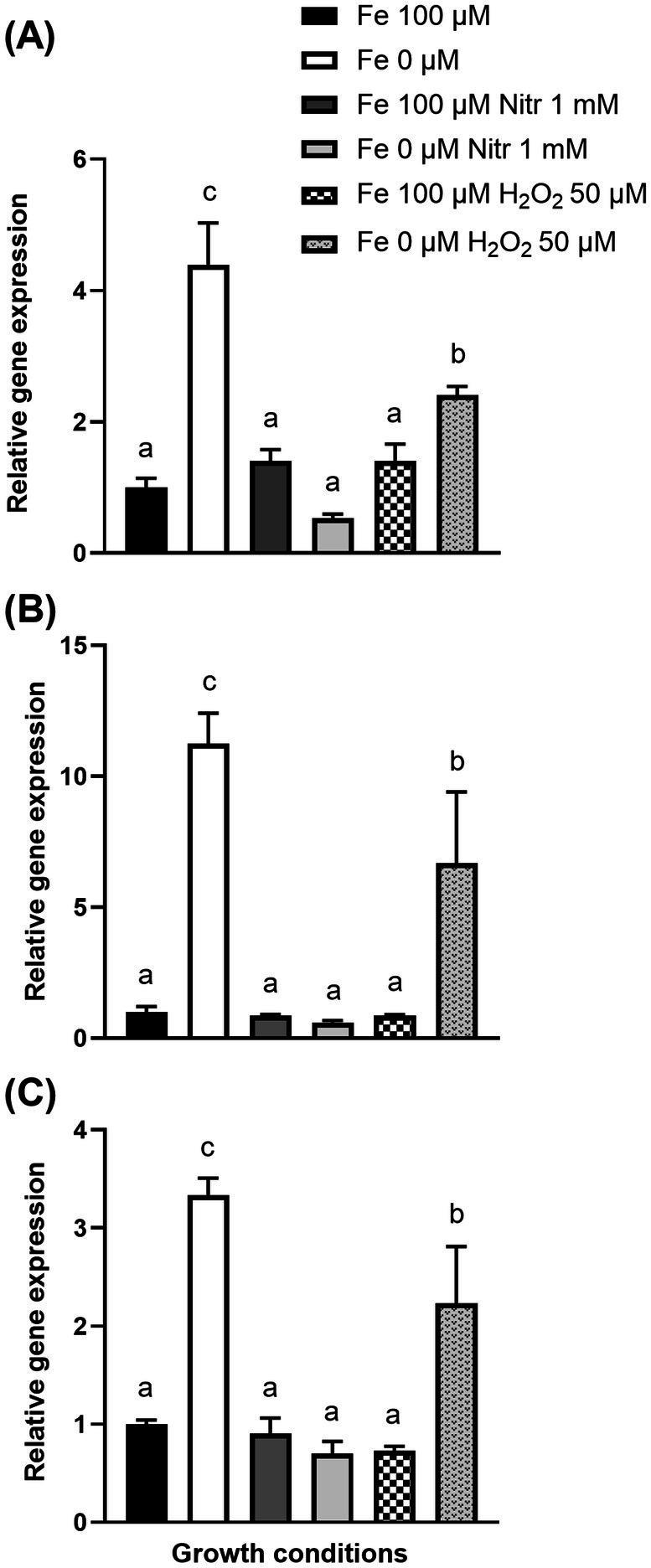
Transcription levels of pyochelin biosynthesis genes SCAB_1481 **(A)**, SCAB_1381 **(B)**, SCAB_1391 **(C)** in *Streptomyces scabies* after 24 h incubation in MS-S medium during oxidative stress induced by sodium nitroprusside (Nitr) or hydrogen peroxide (H_2_O_2_) added after 24 h cultivation. Relative expression levels were determined using *gyrA* as a reference gene. The values are the average of four biological replicates (±standard error of the mean). Data with the same letter are not significantly different (one-way ANOVA, *p* < 0.05, followed by Fisher’s LSD test).

To determine the impact of oxidative stress on pyochelin biosynthesis upon potato tuber-*S. scabies* interactions, the expression of pyochelin biosynthesis genes under *S. scabies* EF-35 exposure to 1 mM sodium nitroprusside and 50 μM of hydrogen peroxide was investigated (Figure 5). Sodium nitroprusside and hydrogen peroxide cause oxidative stress through the formation of reactive oxygen species (ROS) and iron-related radical reactions (Tanou et al., 2012; Ransy et al., 2020).

Iron deprivation in the MS-S medium, caused an increase in the expression of the SCAB_1481, SCAB_1381, and SCAB_1391 genes by 4.4 ± 0.6, 11.2 ± 1.2, and 3.3 ± 0.2-fold, respectively, compared with expression in control MS-S containing iron (Fe^2+^ 100 μM). Exposure of *S. scabies* EF-35 to 1 mM nitroprusside in MS-S containing 100 μM Fe^2+^ did not significantly alter the expression of pyochelin biosynthesis genes comparing to iron-containing MS-S. The addition of nitroprusside to the iron-free medium did not significantly affect the gene expression levels that were comparable to the initial expression in the control MS-S medium containing 100 μM iron (Figure 5).

Addition of 50 μM peroxide to MS-S with 100 μM iron did not induce significant change in the expression of pyochelin genes. When peroxide was added to the medium without iron, the transcriptional level of SCAB_1481, SCAB_1381, and SCAB_1391 increased by 2.4 ± 0.13, 6.7 ± 2.7, and 2.2 ± 0.58 respectively, compared to control conditions (100 μM iron).

## 4 Discussion

### 4.1 Potato microtubers: a model to study plant-microbe interactions

Although previous studies examined the effect of potato plant cell constituents (Lauzier et al., 2008; Komeil et al., 2014; Padilla-Reynaud et al., 2015; Planckaert et al., 2018) on *S. scabies* proteome and secretome, aim of this work was to document the changes in *S. scabies* protein profiles during its interactions with potato tubers. The model used in this study allows the interaction of *S. scabies* with living, physiologically active potato microtubers. However, this model may not necessarily reflect the interactions between *S. scabies* and potato encountered in the field, since metabolic and physiological differences may be observed between tubers and microtubers (Donnelly et al., 2003). Nevertheless, microtubers have been shown to be a reliable model research system (Coleman et al., 2001). Here, we selected two potato cultivars with different levels of common scab resistance: Russet Burbank (RB), which is moderately resistant and Yukon Gold (YG), which is susceptible. This approach made possible to reveal protein determinants that are common in the response of *S. scabies* to different potato cultivars.

### 4.2 Proteins involved in *Streptomyces scabies* secondary metabolites production

The pathogenicity of *S. scabies* is attributed to the production of various secondary metabolites including thaxtomins, concanamycins and coronafacic acid (Bignell et al., 2014). Interestingly, in this study, the functional group including secondary metabolism and differentiation proteins showed the highest NSAF variation within the proteomes of *S. scabies* grown in the absence or the presence of microtubers. The induction of protein A0A0L0L3E1, a carbamoyltransferase involved in concanamycin synthesis, in presence of microtubers, suggests that this toxin plays a role in the *S. scabies*-tuber interaction. The production of concanamycin in the presence of potato tubers is also supported by the fact that proteomic analysis revealed seven other proteins involved in the biosynthesis of concanamycins (C9YYJ0, C9YYJ1, C9YYJ3, C9YYJ6, C9YYI6, C9YYI7, and C9YYI9). The spectral counts of these proteins were below three, however all of them were induced 1.9 to 6.1-fold in the presence of microtubers (Supplementary Table S2). The phytotoxin concanamycin was proposed to play a potential role in the pathogenicity of *S. scabies* and this phytotoxin has been shown to promote the development of deep pitted lesions in potato tubers infected with common scab (Natsume et al., 2017). The overproduction of proteins linked to concanamycin biosynthesis may be linked to the release of cello-oligosaccharides from the plant cell walls since these oligosaccharides induce the production of concanamycins (Deflandre et al., 2022).

It has been proposed that the pathogenic lifestyle of *S. scabies* was characterized by a specialized set of secondary metabolites that mediate interactions between *S. scabies* and its plant host (Liu et al., 2021). In this study, proteins possibly involved in the synthesis of unidentified secondary metabolites were detected in *S. scabies* and overproduced in the presence of microtubers. These include C9ZD03, which is an AurF domain-containing protein. This domain is associated with non-heme di-iron monooxygenase that catalyzes oxidation of aminoarenes to nitroarenes. In *Streptomyces thioluteus*, AurF catalyzes the formation of the polyketide synthase starter unit p-nitrobenzoic acid in the biosynthesis of antibiotic aureothin (Choi et al., 2008). Another protein is C9ZCZ1, a putative carbamoyltransferase that exhibits amino sequence similarity to the decarbamoylnovobiocin carbamoyltransferase involved in the synthesis of novobiocin in *Streptomyces lavendulae* (Etienne et al., 1991). C9YYT5 is an amidohydro-rel domain-containing protein that has been shown to be produced in the presence of suberin (Komeil et al., 2014). C9YUJ1 exhibited homology with the 2,3-diaminopropionate biosynthesis protein SbnB. The gene encoding C9YUJ1 is located in *S. scabies* genome at close proximity of a gene homolog to sbnA and a gene encoding a nonribosomal petide synthetase. Biosynthesis of L-2,3-diaminopropionate, a non-protein amino acid, depends on the activity of both SbnA and SbnB. L-2,3-diaminopropionate serves as a precursor to siderophores and antibiotics (Kobylarz et al., 2014). Finally, a polyketide cyclase/dehydrase (C9ZEV2) was also overproduced in the presence of potato tubers. This protein contains the following motifs: Polyketide_cyc2, Polyketide_cyc and Bet_v_1. While rare bacterial proteins share similar motifs with C9ZEV2, these motifs are found in plant proteins that act as receptors for the plant hormone abscisic acid. While there is no evidence that C9ZEV2 could serve as abscisic acid receptor, this hormone is involved in the regulation of wound-induced suberization (Lulai et al., 2008) and interaction of *S. scabies* with developing potato tubers is known to stimulate suberin biosynthesis (Thangavel et al., 2016). Studying the role of these proteins points to future avenues to unravel the mechanism of interaction between *S. scabies* and its host plant.

Thaxtomin is a predominant virulence factor essential for pathogenicity (Lawrence et al., 1990; Goyer et al., 1998; Joshi M. V. et al., 2007; Joshi M. et al., 2007; Bignell et al., 2014). No proteins encoded by the thaxtomin biosynthetic gene cluster were detected via this proteomic analysis. However, we showed that this secondary metabolite was synthesized by *S. scabies* in the presence of both cultivars. Production of thaxtomin A is an integral feature of the switch to secondary metabolism and morphological differentiation in *S. scabies* (Lerat et al., 2009; Bignell et al., 2014). It was also shown in other *Streptomyces* species (e.g., *S. coelicolor*) that a nitrogen oxide metabolic cycle (arginine → NO → NO_3_^−^ → NO_2_^−^) and nitrite removal system is involved in the regulation of differentiation and secondary metabolism initiation (Sasaki et al., 2016; Honma et al., 2022). Detection of nitrite in *S. scabies* culture medium in the presence of potato microtubers suggests that *S. scabies* also retains the nitrogen oxide cycle and the nitrite removal system or that NO, produced in excess by TxtD during of thaxtomin biosynthesis (Johnson et al., 2008; Vincent and Bignell, 2024), is converted into nitrite, its inert metabolic form. It is unknown if nitrite production plays a role in *S. scabies* pathogenicity but *Mycobacterium tuberculosis*, a human actinobacterial pathogen, produces profuse amount of nitrite while infecting human macrophages. This production of nitrite in *M. tuberculosis* induces the expression of genes associated with adaptation to various stress, including oxidative stress and iron deprivation (Cunningham-Bussel et al., 2013).

### 4.3 Proteins involved in *Streptomyces scabies* stress and defense mechanisms

The development of mutual plant-pathogen interactions may be indicated by increased production of bacterial proteins belonging the functional group of stress and defense mechanisms (Leonard et al., 2016). In this study, several proteins associated with stress adaptation were overproduced. The phage shock protein A (C9YTS9) encoded by *pspA* was among the stress proteins that were at least twice more abundant in presence of microtubers. In *Streptomyces lividans*, *pspA* overexpression improves the Tat-dependent protein secretion (Vrancken et al., 2007). PspA is known to be strongly induced under stress conditions that alter membrane integrity (Vrancken et al., 2008). This suggests that the presence of potato tubers may affect *S. scabies* membrane structure. This may be due to the action of *S. scabies* suberinase (Jabloune et al., 2020), as suberin fatty acid monomers are known as membrane perturbants (Douliez, 2004).

Other upregulated proteins appeared to play a role in redox stress perception and adaptation to oxidative stress. C9ZEV0 is a PAS-domain containing protein. The exact function of this protein has not been investigated. However, the PAS domain is involved in sensing redox change and oxygen level (Taylor and Zhulin, 1999). C9YVQ3 (NreC) belongs to the superfamily of bacterial sensor-transmitter response regulators. The protein responds to oxygen levels and acts as an activator of transcription of genes involved in nitrite reduction (Fedtke et al., 2002). C9ZGD0 is a serine/threonine protein kinase that shows high level of homology with StkP, a global regulator of gene expression in *Streptococcus pneumoniae*. This protein kinase is associated with resistance to various environmental stress and controls the expression of a large set of genes including those involved in iron uptake and oxidative stress (Nováková et al., 2010). The iron-regulated ABC transporter SufB and the Fe-S cluster assembly protein SufD are part of the SUF system. This system is responsible for the assembly of iron–sulfur (Fe-S) clusters, cofactors for numerous proteins involved in electron transfer and sensing of oxygen and iron that operates under iron starvation and oxidative stress (Roche et al., 2013). C9YU91 and A0A1W7CYX9 are peroxidases, reaction oxygen species (ROS)-detoxifying enzymes, while C9YZM9 was identified as organic peroxide resistance protein OhrB.

The determination of upregulated proteins involved in sensing and protecting against oxidative stress reveals a new type of interaction between *S. scabies* and potato tubers that has not been previously described. The development of oxidative stress, detected by proteomic analysis, was confirmed by direct monitoring of the hydrogen peroxide level in the medium during the interaction of *S. scabies* with tubers (Figure 4). Despite the fact that the development of oxidative stress is an important part of the plant defense mechanism against pathogenic microorganisms, when analyzing potato tubers presenting common scab symptoms, no periderm-associated proteins were identified that could directly confirm the production or presence of ROS (Xia et al., 2022). However, an increase in the content of polyphenol oxidase (PPO, Q06355) in the peridermal zone of infected tubers may indicate a possible induction of oxidative stress regulation (Boeckx et al., 2015). In addition to its putative involvement in the oxidation of phenols to quinones (Parveen et al., 2010), chloroplast PPOs may act as regulators of oxygen availability, activated by elevated oxygen levels or play a role of enzymatic antioxidants (Mayer and Harel, 1979; Vaughn and Duke, 1984; Hasanuzzaman et al., 2020). The analysis performed by Xia et al. (2022) revealed a decrease in the content of peroxidase 12 and suberization-associated anionic peroxidase 2-like in tuber flesh of infected potato. The authors suggested the redox homeostasis of tuber flesh cells might be compromised by the common scab-inducing pathogen as it has been suggested that thaxtomin A may play a role in alleviating oxidative stress and ROS-induced programmed cell death (Awwad et al., 2019; Xia et al., 2022).

### 4.4 Importance of iron in *Streptomyces scabies*—potato microtubers interactions

In this study, the overproduction of serine/threonine-protein kinase (StkP), iron-regulated ABC transporter (SufB) and Fe-S cluster assembly protein (SufD) suggests a role of iron in *S. scabies*-potato tuber interactions. The involvement of iron in the interaction is also supported the overproduction by *S. scabies* of proteins linked to the siderophore pyochelin biosynthesis (C9ZCY1 and C9ZCX9) in the presence of potato microtubers. Four other proteins linked to the biosynthesis of the siderophore pyochelin (C9ZCY0, C9ZCY8, C9ZCY5, and C9ZCY3) were found in the proteomic analysis, however, they did not meet the selection criteria of at least three spectral counts (Supplementary Table S2). Analysis of the expression of genes involved in pyochelin biosynthesis confirmed an induction caused by the presence of host microtubers (Supplementary Table S3; Figure 3B). Induction of the pyochelin biosynthesis operon was also induced by the absence of iron in the nutritive medium. However, the most pronounced inducing effect was exerted by the combined presence of microtubers and the absence of iron in the medium. These data give support to the possible competition for iron present in the environment. The bioavailability of iron is influenced by its oxidation state. In soil, iron occurs predominantly as Fe^+3^ oxides, which is biologically unavailable form (Bodek et al., 1988). This proteomic study suggests that iron acquisition mechanisms may play a role in pathogenicity. Seipke et al. (2011) suggested that pyochelin biosynthesis was not necessary for *S. scabies* pathogenicity since a mutant deficient in the synthesis of pyochelin was still virulent on potato tuber slices. However, a pathogenicity test on potato tuber slices may not have been relevant since the tuber slicing may have caused iron release from plant tissue.

ICP-MS analysis of iron content showed that interactions between *S. scabies* and tubers cause a significant reduction in iron content in the growth medium. This lower iron content could be due to a nutrient modulation by tubers in response to the presence of *S. scabies*. It was previously demonstrated that nutrient uptake in plants could be affected by bacterial infection and that nutrient status influenced resistance to pathogens (Hood and Skaar, 2012; Nicolas et al., 2019). When only plant or bacterial cells are present in the system, iron requirements are low and the pyochelin proteins were not produced. The presence of potato microtubers appears to be required for inducing production of pyochelin biosynthetic proteins as no significant reduction in the amount of iron was observed when the pathogen was cultivated in the presence of potato exudates. Under those conditions, higher amounts of iron were even detected in the growth medium. This data suggests that accumulation of some plant metabolites in the exudates or a depletion of some minerals have caused a higher level of mortality in bacterial cells and a subsequent release of iron in the culture medium.

Previous studies have provided indirect data related to changes in the amount and distribution of iron in potato tubers or surrounding soil can affect the severity of common scab infection. While searching for factors associated with natural soil suppressivity to potato common scab, the elevated iron content in the periderm of potato tubers was detected in suppressive fields (Sagova-Mareckova et al., 2015). The idea that iron supplementation could directly inhibit the growth or virulence of *S. scabies* in addition to modulation of host defense was proposed by Sarikhani et al. (2017). In *Arabidopsis*, it has been shown that iron deficiency results in an inability to generate ROS in response to pathogens (Kieu et al., 2012). More recently, it was proposed that enhanced iron storage in thaxtomin A-habituated YG somaclone tubers overexpressing ferritin may contribute to increasing resistance to *S. scabies* by limiting iron availability to the pathogen (Labidi et al., 2023).

Since field studies suggest a role for iron in the development of common scab disease, the next important step will be to analyze the effect of mutations in the siderophore genes of *S. scabies* on the development of disease symptoms. These studies will provide a clearer answer to the importance of iron in *S. scabies*-host interactions.

### 4.5 Evidence of oxidative stress during potato microtubers—*Streptomyces scabies* interactions

As expected, the application of oxidative stress using hydrogen peroxide and nitroprusside did not induce the expression of pyochelin genes, which indicates the absence of a direct induction of pyochelin biosynthesis by oxidative stress (Figure 5). Nevertheless, the crosstalk between oxidative stress and iron homeostasis may be regulated post-transcriptionally by aconitate hydratase (aconitase, ADL12_38700, Supplementary Table S3) (Hantke, 2001; Andrews et al., 2003; Michta et al., 2012). The significant accumulation of the bacterial protein aconitase in the presence of microtubers of both potato cultivars may influence the regulation of iron transport and storage. Aconitase has a dual function as an enzyme involved in the initial stages of the TCA cycle and as a regulator of iron homeostasis in apo-form (apoprotein IRP1). In the presence of oxidative stress, aconitase loses its 4Fe-4S cofactor and gains RNA binding properties as IRP1. IRP1 sequence has an affinity for iron-responsive elements (IREs) – conserved stem-loop structures in the mRNA, by binding to which it is able to stabilize messenger RNA molecules and enhance the synthesis of iron accumulation (ferritin) and transport (import transferrin receptor and export ferroportin) proteins (Narahari et al., 2000; Austin et al., 2015).

Oxidative stress is a known mechanism for protecting plant cells from various pathogens of bacterial and fungal origins (Tripathy and Oelmüller, 2012). The most frequently plant produced ROS in plant-pathogen interactions are products of the sequential reduction of molecular oxygen (Wojtaszek, 1997). Oxidative burst and H_2_O_2_ production were shown to play an important role in potato resistance to several phytopathogens (Wu et al., 1995; Taheri and Kakooee, 2017). The overproduction of peroxidases and peroxide resistance proteins by *S. scabies* in the presence of potato microtubers suggests that potato microtuber cells react to the presence of *S. scabies* by producing ROS. Although oxidative stress was not previously reported in potato tubers during potato - *S. scabies* interaction, some evidence suggests that ROS may be produced by plant cells. Pre-treatment of *Arabidopsis* cell cultures with hydrogen peroxide reduces cell death caused by thaxtomin A (Awwad et al., 2019) which is consistent with previously characterized *Arabidopsis* mutant plants resistant to thaxtomin A (Scheible et al., 2003). These mutant plants displayed increased accumulation of ROS and exhibited increased resistance to virulent pathogens (Huang et al., 2013). The present study also suggests that the production of ROS may be one of the protective mechanisms of potato tuber cells, reducing sensitivity to *S. scabies* infection.

Another possibility could be the production of ROS by *S. scabies* cells. Typically, the development of oxidative stress is a deadly weapon against various types of bacteria. However, it has been shown that heterotrophic bacteria from various sources can produce extracellular superoxide. Superoxide production has been reported by bacteria belonging to various aquatic and terrestrial environments, including soil inhabiting *Actinobacteria* (*Arthrobacter* sp. A528) (Diaz et al., 2013). The mechanisms of extracellular production of ROS by bacterial cells are not well understood. The discovery and biochemical characterization of bacterial transmembrane enzymes of the NADPH oxidase family led to the idea that bacterial cells can produce extracellular ROS, which are involved in cell-to-cell signaling (Hajjar et al., 2017). An analysis of extracellular proteins from *S. scabies* revealed two oxidoreductases, putative FAD/NAD(P) binding oxidoreductase C9ZGW6 and a putative secreted oxidoreductase C9Z871, which might be involved in the production of extracellular superoxide (Komeil et al., 2014).

For some other potato diseases, such as late blight induced by *Phytophthora infestans*, it has been shown that the level of resistance of potato cultivars was related to the production of ROS in response to the pathogen (El_Komy et al., 2020). Research aimed at assessing the oxidative response of potato plants when infected with *S. scabies* may be a useful tool for common scab management and crop development.

## 5 Conclusion

In conclusion, the observed changes in several functional groups of proteins influencing the metabolism of *S. scabies* have been documented in the presence of microtubers of two potato cultivars. We have shown evidence of oxidative stress that develops from the interactions of the pathogen with potato tubers. Additionally, our study suggests that pyochelin plays a significant role in pathogenicity of *S. scabies* during the interaction with potato tubers.

## Data Availability

The datasets presented in this study can be found in online repositories. The names of the repository/repositories and accession number(s) can be found at: https://doi.org/10.5683/SP3/N7YXNC.
