# Supplementary material for: Proteomics fingerprinting reveals importance of iron and oxidative stress in Streptomyces scabies–Solanum tuberosum interactions
Source: Front Microbiol. 2024 Oct 2;15:1466927. doi: 10.3389/fmicb.2024.1466927 (PMC11479980; doi:10.3389/fmicb.2024.1466927)
Supplement: Supplementary file 1 [file Table_1.docx]

Supplementary Material

Proteomics fingerprinting reveals importance of iron and oxidative stress in *Streptomyces scabies* - *Solanum tuberosum* interactions

Lauriane Giroux^†^, Iauhenia Isayenka^†^, Sylvain Lerat, Nathalie Beaudoin and Carole Beaulieu*

^†^ These authors contributed equally to this work and share first authorship

Département de biologie, Centre SÈVE, Université de Sherbrooke, Sherbrooke, QC, Canada

*** Correspondence:**Carole Beaulieu
carole.beaulieu@usherbrooke.ca


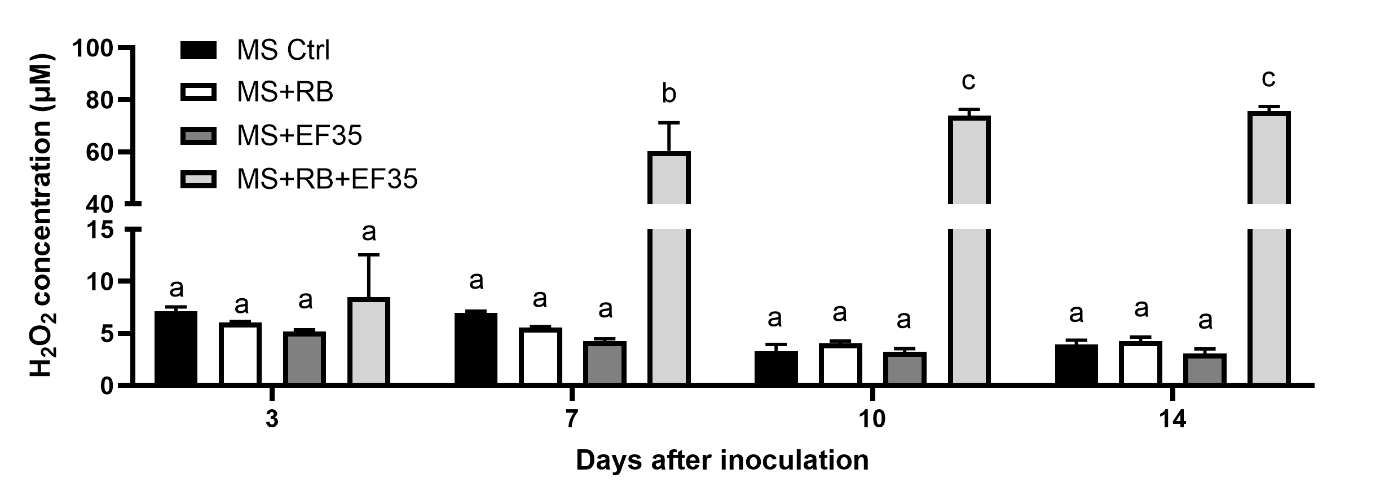


**Supplementary Figure S1.** Peroxide content in the nutrient medium containing *Streptomyces scabies* EF-35 and/or microtubers of potato cv. Russet Burbank compared to the medium devoid of plant material and bacterial cells. MS Ctrl: MS-S medium, without biological material; MS+RB: MS-S medium containing microtubers of cv. Russet Burbank; MS+EF-35: MS-S medium inoculated with *S. scabies* EF-35; MS+RB+EF-35: containing both microtubers and bacterial cells. The values are the mean of three biological replicates (± standard error of the mean) for MS Ctrl and four biological replicates for all other conditions. Data with the same letter are not significantly different (*p* < 0.05, two-way ANOVA followed by Fisher’s LSD test).
