# Supplementary material for: Proteomics fingerprinting reveals importance of iron and oxidative stress in Streptomyces scabies–Solanum tuberosum interactions
Source: Front Microbiol. 2024 Oct 2;15:1466927. doi: 10.3389/fmicb.2024.1466927 (PMC11479980; doi:10.3389/fmicb.2024.1466927)
Supplement: Supplementary file 2 [file Table_2.docx]

Supplementary Material

Proteomics fingerprinting reveals importance of iron and oxidative stress in *Streptomyces scabies* - *Solanum tuberosum* interactions

Lauriane Giroux^†^, Iauhenia Isayenka^†^, Sylvain Lerat, Nathalie Beaudoin and Carole Beaulieu*

^†^ These authors contributed equally to this work and share first authorship

Département de biologie, Centre SÈVE, Université de Sherbrooke, Sherbrooke, QC, Canada

*** Correspondence:**Carole Beaulieu
carole.beaulieu@usherbrooke.ca

**Supplementary Table S1.** List of primers used in this study.

| **Gene assignation** | **Predicted function of the corresponding protein** | **Primer set (5’ to 3’)** | **Reference** |
| --- | --- | --- | --- |
| *gyrA* | Gyrase A | For: GCCATCAACCTCCGTGAAA Rev: CGGATCGATTGTGCCTTCTT | 1 |
| *txtD* | Nitric oxide synthase | For*:* GAGCTGGTCTTGGAGGTCCCTATC Rev: CGCATGTTGGAGATGACGGGTACG | 1 |
| SCAB_1381 | Putative salicylate synthase | For: GCACGAACACGCCATTTC Rev: GCTCCTTGATCGACATGTACTC | This study* |
| SCAB_1391 | Conserved hypothetical protein | For: CTACGTCGAGAGCGAAATCATC Rev: CAGGCCTTGAGGTCGAAAG | This study |
| SCAB_1481 | L-cysteine-[L-cysteinyl-carrier protein] ligase PchE | For: GGCTGCTGGACATGGAC Rev: GGTTGAGCTTGTTGGTGAGA | This study |
| SCAB_31841 | Nitric oxide synthase oxygenase | For: GAGCTGGTCTTGGAGGTCCCTATC Rev: CGCATGTTGGAGATGACGGGTACG | This study |

1. Lerat, S., Simao-Beaunoir, A.-M., Wu, R., Beaudoin, N., and Beaulieu, C. (2010). Involvement of the plant polymer suberin and the disaccharide cellobiose in triggering thaxtomin A biosynthesis, a phytotoxin produced by the pathogenic agent *Streptomyces scabies*. Phytopathology 100, 91–96.

* Primers were designed using PrimerQuest tool (Integrated DNA Technologies, Inc.).

**Supplementary Table S3.** Proteins with a normalized spectral abundance factor value twice higher in the presence of both potato cultivars than in a pure culture of *Streptomyces scabies* EF-35.

| Uniprot accession number | Putative function of the protein | Gene assignation | Normalized spectral abundance factor (%) * | | | |
| --- | --- | --- | --- | --- | --- | --- |
|  |  |  | EF-35 | EF-35 + RB^†^ | EF-35 +  YG | |
| Amino acid metabolism | | | | | | |
| C9ZGG2 | 4-aminobutyrate aminotransferase | SCAB_18021 | 0.0330 | 0.148 | | 0.085 |
| C9Z7K3 | Carbamoyl-phosphate synthase | SCAB_75231 (*pyrA carB*) | 0.004 | 0.015 | | 0.015 |
| C9Z7B7 | Aspartate-semialdehyde dehydrogenase | SCAB_59621 (*asd1 asd*) | 0.003 | 0.033 | | 0.016 |
| C9Z402 | 5-aminovalerate aminotransferase | SCAB_41841 | ND^¶^ | 0.002 | | 0.030 |
| A0A1Q5KV42 | D-3-phosphoglycerate dehydrogenase | AMK31_15210 | 0.021 | 0.043 | | 0.042 |
| A0A0L8MVR0 | Tryptophan synthase | ADK75_14015 (*trpB*) | 0.008 | 0.045 | | 0.038 |
| A0A143C079 | Aminopeptidase N | A4E84_13905 | ND | 0.013 | | 0.013 |
| Carbohydrate metabolism | | | | | | |
| A0A101PMD4 | Transaldolase | AQI96_06170 (*tal*) | 0.039 | 0.141 | | 0.114 |
| A0A1Q5LYT5 | Glucoamylase | AMK09_36290 | ND | 0.029 | | 0.024 |
| A0A0M8WDG6 | Transaldolase | ADL01_09595 (*tal*) | ND | 0.050 | | 0.085 |
| C9ZBJ5 | Alpha-mannosidase | SCAB_15481 | 0.031 | 0.081 | | 0.089 |
| C9ZFY5 | Xylose isomerase | SCAB_79861 (*xylA*) | 0.074 | 0.214 | | 0.166 |
| C9YXN7 | Rhamnosidase | SCAB_22771 | ND | 0.054 | | 0.061 |
| C9YXR3 | 2-oxoacid oxidoreductase | SCAB_37561 | 0.010 | 0.047 | | 0.034 |
| C9ZGR4 | Phosphoenolpyruvate carboxykinase | SCAB_34111 (*pckG*) | 0.009 | 0.021 | | 0.086 |
| C9ZD79 | Sugar phosphate isomerase/epimerase | SCAB_16731 | 0.020 | 0.078 | | 0.127 |
| C9Z9C4 | Mannose-1-phosphate guanyltransferase | SCAB_76231 | 0.007 | 0.027 | | 0.022 |
| C9YXN3 | Beta-glucosidase | SCAB_22731 | ND | 0.009 | | 0.052 |
| C9YT49 | Alditol oxidase | SCAB_19421 | 0.027 | 0.059 | | 0.061 |
| C9Z1V1 | Sugar isomerase | SCAB_9441 | ND | 0.055 | | 0.096 |
| C9YSZ3 | Levansucrase | SCAB_18831 | ND | 0.038 | | 0.027 |
| A0A081XI56 | Glucoamylase | BU52_31710 | ND | 0.023 | | 0.020 |
| A0A170XU30 | Malate dehydrogenase | STXM2123_2743 (*mdh*) | ND | 0.187 | | 0.128 |
| C9Z540 | Glucose-1-phosphate adenylyltransferase | SCAB_11761 (*glgC*) | ND | 0.039 | | 0.022 |
| C9ZD81 | Secreted sugar binding protein | SCAB_16751 | 0.160 | 0.574 | | 0.573 |
| C9Z1U7 | Secreted sugar-binding lipoprotein | SCAB_9401 | 0.004 | 0.060 | | 0.055 |
| C9ZAA7 | Extracellular carbohydrate-binding protein | SCAB_29961 | ND | 0.059 | | 0.110 |
| C9YYL7 | Sugar-binding transport lipoprotein | SCAB_7331 | 0.003 | 0.047 | | 0.040 |
| C9ZDX0 | Secreted monosaccharide-binding protein | SCAB_63951 | 0.013 | 0.038 | | 0.026 |
| C9Z619 | Extracellular carbohydrate-binding lipoprotein | SCAB_74641 | ND | 0.021 | | 0.054 |
| A0A0L8L2H4 | Phosphoenolpyruvate-protein phosphotransferase | ADK37_28190 | ND | 0.016 | | 0.037 |
| C9Z4B0 | Putative oxidoreductase | SCAB_58381 | ND | 0.041 | | 0.056 |
| Coenzyme transport and metabolism | | | | | | |
| C9Z3M4 | Aminotransferase | SCAB_25861 | 0.016 | 0.083 | | 0.069 |
| C9ZBZ9 | Phosphomethylpyrimidine synthase | SCAB_46341 (*thiC*) | 0.002 | 0.019 | | 0.015 |
| Energy production and conversion | | | | | | |
| C9YTR4 | Pyruvate dehydrogenase | SCAB_67031 | ND | 0.050 | | 0.065 |
| C9YZN8 | Aldehyde dehydrogenase | SCAB_70651 | 0.015 | 0.045 | | 0.032 |
| C9Z4N1 | Putative oxidoreductase | SCAB_87211 | 0.014 | 0.041 | | 0.042 |
| C9YXV0 | NADH dehydrogenase subunit | SCAB_37971 (*nuoM*) | 0.021 | 0.105 | | 0.051 |
| C9Z5Y8 | Aldo/keto reductase | SCAB_74311 | 0.007 | 0.040 | | 0.028 |
| C9Z3L5 | Aldehyde dehydrogenase | SCAB_25771 | ND | 0.015 | | 0.027 |
| A0A086GWS5 | Succinate dehydrogenase | IQ62_16970 (*sdhA*) | 0.008 | 0.016 | | 0.050 |
| A0A100JPX1 | Pyruvate phosphate dikinase | SsS58_03888 (*ppdK_3*) | ND | 0.004 | | 0.031 |
| C9YXC1 | Alcohol dehydrogenase class III | SCAB_7101 (*adhC*) | 0.039 | 0.089 | | 0.123 |
| A0A101JBT2 | Aconitate hydratase | ADL12_38700 (*acnA*) | 0.033 | 0.089 | | 0.077 |
| A0A0L0KSG9 | Dihydrolipoyl dehydrogenase | IQ64_30375 | 0.093 | 0.653 | | 0.350 |
| A0A0C5GBX8 | Dihydrolipoyl dehydrogenase | TU94_09485 | ND | 0.309 | | 0.153 |
| C9Z1V2 | Rhamnulose-1-phosphate aldolase | SCAB_9451 | ND | 0.008 | | 0.035 |
| Inorganic ion metabolism | | | | | | |
| C9YUJ7 | Nitrite/sulphite reductase | SCAB_19771 | 0.014 | 0.031 | | 0.030 |
| C9ZCX9 | Pyochelin biosynthesis | SCAB_1391 | ND | 0.034 | | 0.056 |
| C9ZCY1 | AMP-binding NRPS ligase (pyochelin biosynthesis) | SCAB_1411 | 0.006 | 0.026 | | 0.021 |
| Lipid metabolism | | | | | | |
| C9Z865 | Fatty acid oxidative multifunctional enzyme | SCAB_13351 (*fadB1*) | 0.006 | 0.012 | | 0.016 |
| V6KKE6 | Biotin carboxyl carrier protein | M878_15880 | 0.007 | 0.018 | | 0.014 |
| Nucleotide metabolism | | | | | | |
| C9YWT5 | Pseudouridine-5'-phosphate glycosidase | SCAB_69391 (*psuG*) | 0.014 | 0.068 | | 0.073 |
| Post-translational modification, protein turnover and proteolysis | | | | | | |
| A0A1I2UI89 | Tricorn protease homolog | SAMN02787118_12824 | ND | 0.020 | | 0.023 |
| C9Z7I2 | Peptidase | SCAB_60251 | 0.120 | 0.354 | | 0.324 |
| A0A385DHU0 | Pup-protein ligase | D0C37_27930 (*pafA*) | ND | 0.095 | | 0.131 |
| Replication, recombination and DNA repair | | | | | | |
| C9YVZ8 | Helicase SNF2 family protein | SCAB_21231 | 0.006 | 0.019 | | 0.016 |
| Transcription and RNA processing | | | | | | |
| A0A0M9ZEE2 | Ribonuclease J | ADL00_40440 (*rnj*) | ND | 0.058 | | 0.033 |
| G1AQV2 | RNA polymerase | *rpoB* | 0.030 | 0.086 | | 0.073 |
| Translation, ribosomal structure and biogenesis | | | | | | |
| C9YW71 | 50S ribosomal protein L16 | SCAB_36871 (*rplP*) | 0.043 | 0.092 | | 0.115 |
| A0A0C1XJ11 | Elongation factor Tu | HY68_07095 (*tuf*) | ND | 0.156 | | 0.414 |
| Secondary metabolism and differentiation | | | | | | |
| C9YUJ1 | Ornithine cyclodeaminase | SCAB_19711 | 0.054 | 0.127 | | 0.116 |
| C9ZD03 | AurF domain-containing protein | SCAB_1641 | ND | 0.042 | | 0.042 |
| C9ZEV2 | Polyketide cyclase/dehydrase | SCAB_17551 | 0.044 | 0.126 | | 0.112 |
| C9ZCZ1 | Carbamoyltransferase | SCAB_1511 | ND | 0.124 | | 0.123 |
| C9YYT5 | Amidohydrolase-related domain-containing protein | SCAB_8041 | 0.004 | 0.033 | | 0.043 |
| A0A0L0L3E1 | Carbamoyltransferase (concanamycin) | IQ64_11950 | 0.008 | 0.041 | | 0.022 |
| Stress and defense mechanism | | | | | | |
| C9ZGD0 | Serine/threonine protein kinase StkP | SCAB_3621 | 0.013 | 0.112 | | 0.133 |
| C9YVQ3 | Oxygen regulatory protein NreC | SCAB_6061 | 0.026 | 0.064 | | 0.125 |
| C9YY94 | Iron-regulated ABC transporter SufB | SCAB_70031 | 0.019 | 0.042 | | 0.046 |
| C9YY95 | Fe-S cluster assembly protein SufD | SCAB_70041 | 0.021 | 0.044 | | 0.043 |
| C9YTS9 | Phage shock protein A | SCAB_67181 | 0.039 | 0.215 | | 0.106 |
| C9ZEV0 | PAS domain-containing protein | SCAB_17531 | 0.024 | 0.052 | | 0.056 |
| C9YZM9 | Organic hydroperoxide resistance protein | SCAB_55571 | 0.048 | 0.192 | | 0.203 |
| C9YU91 | Non-heme chloroperoxidase | SCAB_4601 (*cpo*) | ND | 0.070 | | 0.115 |
| A0A1W7CYX9 | Catalase-peroxidase | CAG99_14675 (*katG*) | ND | 0.054 | | 0.056 |
| General function predicted only | | | | | | |
| A0A0M9YG60 | Histidine kinase | ADK54_14870 | ND | 0.015 | | 0.011 |
| C9ZCZ9 | Putative hydrolase | SCAB_1601 | ND | 0.050 | | 0.037 |
| C9Z0G5 | Acyl-coenzyme A thioesterase THEM4 | SCAB_24031 | 0.016 | 0.033 | | 0.100 |
| M3DHC3 | DSBA domain-containing protein | SBD_3460 | 0.010 | 0.059 | | 0.021 |
| A0A0F5VRB1 | ATP-binding protein | TN53_32520 | 0.014 | 0.032 | | 0.027 |
| C9Z1Q3 | Putative regulatory protein | SCAB_85931 | 0.022 | 0.047 | | 0.051 |
| A0A1C6NA13 | Histidine kinase | YWIDRAFT_01395 | ND | 0.012 | | 0.006 |
| Unknown function | | | | | | |
| C9YY57 | Secreted protein | SCAB_54651 | 0.163 | 0.553 | | 0.340 |
| C9YUE9 | Putative secreted protein | SCAB_5221 | 0.168 | 0.495 | | 0.370 |
| A0A117EFE3 | Tetratricopeptide repeat protein | SsS58_05887 | 0.010 | 0.027 | | 0.024 |
| C9ZAJ4 | DNA-binding protein | SCAB_45671 | 0.045 | 0.141 | | 0.110 |
| C9YZB7 | S-adenosyl methyltransferase | SCAB_39001 | 0.014 | 0.041 | | 0.044 |
| C9Z8Q6 | Putative secreted protein | SCAB_44691 | ND | 0.020 | | 0.016 |
| C9YYX4 | Band 7 domain-containing protein | SCAB_23161 | ND | 0.003 | | 0.089 |
| C9Z623 | Putative secreted protein | SCAB_74681 | ND | 0.017 | | 0.015 |
| A0A0M8WS29 | DUF885 domain-containing protein | ADL04_37195 | 0.008 | 0.021 | | 0.024 |

Analysis was performed after 5 days of growth in MS-S medium (see Materials and methods for details).

*Data are the mean of four biological replicates

^†^RB: Russet Burbank and YG: Yukon Gold

^¶^ND: Not detected

**Supplementary Table S4.** Proteins with a normalized spectral abundance factor value twice lower in the presence of both potato cultivars than in a pure culture of *S. scabies* EF-35.

| Uniprot accession number | Putative function of the protein | Gene assignation | Normalized spectral abundance factor (%) * | | |
| --- | --- | --- | --- | --- | --- |
|  |  |  | Pure culture | EF-35+ RB^†^ | EF-35+ YG |
| Amino acid metabolism | | | | | |
| C9Z599 | D-3-phosphoglycerate dehydrogenase | SCAB_27051 (*serA*) | 0.221 | 0.106 | 0.109 |
| A0A100JIC3 | Betaine aldehyde dehydrogenase | SsS58_00441 (*gbsA_1*) | 0.130 | 0.065 | 0.097 |
| C9YYA5 | 2,3,4,5-tetrahydropyridine-2,6-dicarboxylate N-succinyltransferase | SCAB_70141 (*dapD*) | 0.116 | 0.058 | 0.049 |
| C9YVH4 | Indole-3-glycerol phosphate synthase | SCAB_68491 (*trpC*) | 0.105 | 0.048 | 0.047 |
| C9ZBW7 | Succinyl-diaminopimelate desuccinylase | SCAB_31231 (*dapE*) | 0.056 | 0.025 | 0.021 |
| C9ZA98 | Polar amino acid transport system | SCAB_29881 (*atrA*) | 0.132 | 0.042 | 0.065 |
| C9YWN6 | Branched-chain amino acid transport system permease | SCAB_68891 | 0.099 | 0.048 | 0.030 |
| C9Z5D2 | Oligopeptide ABC transporter component | SCAB_27391 | 0.087 | 0.020 | 0.037 |
| Carbohydrate metabolism | | | | | |
| C9YSV0 | Probable phosphoketolase | SCAB_4201 | 0.052 | 0.015 | 0.021 |
| C9Z737 | Ricin-type beta-trefoil lectin domain protein | SCAB_43661 | 0.043 | 0.015 | 0.021 |
| C9YUA4 | Glyceraldehyde-3-phosphate dehydrogenase | SCAB_4751 (*gap*) | 0.054 | 0.008 | 0.003 |
| A0A089X9G2 | Phosphoglycerate kinase | SGLAU_08735 (*pgk*) | 0.067 | 0.028 | ND^¶^ |
| A0A0U3Q3H2 | ABC transporter substrate-binding protein | AS200_12400 | 0.157 | 0.041 | 0.046 |
| L1KT66 | D-xylose ABC transporter | STRIP9103_07714 | 0.167 | 0.020 | 0.046 |
| A0A1D2IGZ0 | Maltodextrin-binding protein MdxE | APS67_001960 (*mdxE*) | 0.160 | 0.041 | ND |
| Cell wall/membrane/envelope biogenesis | | | | | |
| C9YVC2 | UDP-N-acetylmuramoyl-L-alanyl-D-glutamate—2,6-diaminopimelate ligase | SCAB_67961 (*murE*) | 0.039 | 0.011 | 0.016 |
| C9Z722 | Carboxypeptidase | SCAB_43501 | 0.049 | 0.019 | 0.020 |
| Energy production and conversion | | | | | |
| C9YYW7 | Carbonic anhydrase | SCAB_23091 | 0.187 | 0.092 | 0.072 |
| C9Z770 | Protein-lysine 6-oxidase | SCAB_44001 | 0.104 | 0.038 | 0.022 |
| C9ZDZ7 | Aldo/keto reductase | SCAB_64231 | 0.148 | 0.071 | 0.039 |
| A0A0X3W4F0 | Isocitrate dehydrogenase | ADL30_25195 | 0.077 | 0.025 | 0.038 |
| L1KK73 | Pyruvate dehydrogenase | STRIP9103_02727 | 0.105 | 0.039 | 0.045 |
| A0A1J4NVA0 | 2-oxoglutarate dehydrogenase, E2 component, dihydrolipoamide succinyltransferase | WN71_023115 | 0.187 | 0.092 | ND |
| Lipid metabolism | | | | | |
| C9ZGL8 | Long-chain-fatty-acid-CoA ligase (Lipid biosynthesis proteins) | SCAB_18621 | 0.050 | 0.003 | 0.009 |
| Replication, recombination and DNA repair | | | | | |
| C9ZGH3 | Exonuclease | SCAB_18151 | 0.153 | 0.052 | 0.069 |
| Transcription and RNA processing | | | | | |
| A0A1I2IKF5 | DNA-directed RNA polymerase | SAMN02787118_106325 | 0.325 | 0.126 | 0.137 |
| G1AQW6 | RNA polymerase | *rpoB* | 0.119 | 0.055 | 0.055 |
| Translation, ribosomal structure and biogenesis | | | | | |
| C9Z659 | Alanine--tRNA ligase | SCAB_75061 (*alaS*) | 0.114 | 0.054 | 0.057 |
| C9YW53 | 30S ribosomal protein S13 | SCAB_36691 (*rpsM*) | 0.204 | 0.168 | 0.101 |
| A0A124C3F5 | Peptide deformylase | SsS58_01480 (*def_1 def*) | 0.127 | 0.047 | 0.057 |
| C9YW79 | 30S ribosomal protein S10 | SCAB_36951 (*rpsJ*) | 0.204 | 0.065 | 0.051 |
| A0A1Q5C2Q | Elongation factor Ts | AMK11_31075 (*tsf*) | 0.190 | 0.078 | 0.048 |
| General function predicted only | | | | | |
| C9ZDK2 | Putative ATP binding protein | SCAB_47231 | 0.196 | 0.094 | 0.092 |
| C9Z085 | Putative oxidoreductase | SCAB_8331 | 0.051 | 0.012 | 0.021 |
| C9ZCL9 | Putative secreted tripeptidylaminopeptidase | SCAB_78431 | 0.053 | 0.009 | 0.002 |
| C9ZAH7 | Protein-serine/threonine phosphatase | SCAB_45491 | 0.066 | 0.029 | 0.028 |
| C9ZC02 | Putative phosphoesterase | SCAB_46371 | 0.067 | 0.032 | 0.049 |
| C9Z0S9 | Putative two component system response regulator | SCAB_39621 | 0.088 | 0.035 | 0.034 |
| A0A101PCJ3 | Histidine kinase | AQI96_26145 | 0.026 | 0.009 | 0.007 |
| L7ESI1 | Histidine kinase | STRTUCAR8_07988 | 0.024 | 0.005 | ND |
| Unknown function | | | | | |
| C9YYP6 | S-adenosyl methyltransferase | SCAB_7641 | 0.252 | 0.1245 | 0.125 |
| A0A117EDT7 | BNR/Asp-box repeat protein | SsS58_03198 | 0.130 | 0.0479 | 0.056 |
| C9ZC26 | Putative membrane protein | SCAB_46621 | 0.175 | 0.0676 | 0.072 |

Analysis was performed after 5 days of growth in MS-S medium (see Materials and methods for details).

*Data are the mean of four biological replicates

^†^RB: Russet Burbank and YG: Yukon Gold

^¶^ND: Not detected
