# Supplementary material for: Proteomics fingerprinting reveals importance of iron and oxidative stress in Streptomyces scabies–Solanum tuberosum interactions
Source: Front Microbiol. 2024 Oct 2;15:1466927. doi: 10.3389/fmicb.2024.1466927 (PMC11479980; doi:10.3389/fmicb.2024.1466927)
Supplement: Supplementary file 3 [file Table_3.docx]

Supplementary Material

Proteomics fingerprinting reveals importance of iron and oxidative stress in *Streptomyces scabies* - *Solanum tuberosum* interactions

Lauriane Giroux^†^, Iauhenia Isayenka^†^, Sylvain Lerat, Nathalie Beaudoin and Carole Beaulieu*

^†^ These authors contributed equally to this work and share first authorship

Département de biologie, Centre SÈVE, Université de Sherbrooke, Sherbrooke, QC, Canada

*** Correspondence:**Carole Beaulieu
carole.beaulieu@usherbrooke.ca

**Supplementary Table S2**. Intracellular *Streptomyces scabies* EF-35 proteins with at least three spectral counts in one or more experimental conditions.

| Uniprot Accession Number | Putative function | Gene assignation (name) | Normalized spectral abundance factor (%) * | | |
| --- | --- | --- | --- | --- | --- |
|  |  |  | **EF-35** | **EF-35 + RB**^†^ | **EF-35 + YG**^†^ |
| Amino acid metabolism | | | | | |
| C9YWP0 | Secreted binding protein | SCAB_68931 | 0.4581 | 0.4895 | 0.2877 |
| C9YWB0 | Aminotransferase | SCAB_37271 (*aspC*) | 0.3185 | 0.3762 | 0.4040 |
| C9ZHG5 | Glutamine synthetase | SCAB_66881 (*glnA*) | 0.2955 | 0.2947 | 0.2178 |
| C9Z7C5 | Aminopeptidase | SCAB_59701 | 0.1134 | 0.1010 | 0.1320 |
| C9Z7B6 | Aminopeptidase N | SCAB_59611 (*pepN*) | 0.1102 | 0.1069 | 0.1142 |
| C9ZAQ7 | Secreted metalloendopeptidase | SCAB_61831 | 0.2337 | 0.2515 | 0.3098 |
| C9YZJ1 | Adenosylhomocysteinase | SCAB_55181 (*sahH ahcY*) | 0.1645 | 0.1579 | 0.1255 |
| C9Z599 | D-3-phosphoglycerate dehydrogenase | SCAB_27051 (*serA*) | 0.2053 | 0.1120 | 0.0936 |
| C9YTF8 | Serine hydroxymethyltransferase | SCAB_35641 (*glyA1 glyA*) | 0.1972 | 0.1199 | 0.1352 |
| A0A117EFS2 | S-adenosylmethionine synthase | SsS58_06481 (*metK*) | 0.1643 | 0.2020 | 0.1625 |
| C9YTK4 | Phosphoserine aminotransferase | SCAB_51101 (*serC*) | 0.1183 | 0.1733 | 0.1957 |
| C9Z234 | 4-hydroxy-tetrahydrodipicolinate synthase | SCAB_25191 (*dapA dapA1*) | 0.1680 | 0.2236 | 0.2417 |
| C9Z2Y0 | Peptidase | SCAB_73011 | 0.0890 | 0.0992 | 0.1166 |
| C9YVK4 | Aminopeptidase | SCAB_68791 | 0.0477 | 0.0430 | 0.0397 |
| C9Z5A0 | Ketol-acid reductoisomerase (NADP(+)) | SCAB_27061 (*ilvC*) | 0.1482 | 0.1295 | 0.0853 |
| C9YXA4 | Sarcosine oxidase alpha subunit | SCAB_6931 (*soxA*) | 0.0487 | 0.0303 | 0.0405 |
| A0A100JIC3 | Betaine aldehyde dehydrogenase | SsS58_00441 (*gbsA_1*) | 0.1051 | 0.0601 | 0.0750 |
| A0A0L0KXA4 | 3-isopropylmalate dehydrogenase | IQ64_23960 (*leuB*) | 0.1269 | 0.1209 | 0.1236 |
| A0A100JVB0 | Threonine synthase | SsS58_06775 (*thrC_3*) | 0.1291 | 0.1027 | 0.0975 |
| C9ZC04 | Phenylalanine aminotransferase | SCAB_46391 (*pat*) | 0.1082 | 0.0867 | 0.1020 |
| C9Z4G8 | Glutamine synthetase | SCAB_73851 | 0.0814 | 0.0678 | 0.0752 |
| A0A100JN41 | Threonine synthase | SsS58_02937 (*thrC_2*) | 0.0525 | 0.0732 | 0.0592 |
| C9YVH5 | Tryptophan synthase beta chain | SCAB_68501 (*trpB*) | 0.0529 | 0.0834 | 0.0462 |
| C9Z238 | 4-hydroxy-tetrahydrodipicolinate reductase | SCAB_25231 (*dapB*) | 0.1254 | 0.1268 | 0.1560 |
| C9Z5A2 | Acetolactate synthase | SCAB_27081 | 0.0562 | 0.0316 | 0.0380 |
| C9Z593 | Aldehyde dehydrogenase | SCAB_26991 | 0.0621 | 0.0380 | 0.0690 |
| C9Z8G9 | Diaminopimelate decarboxylase | SCAB_28971 (*lysA*) | 0.0587 | 0.0486 | 0.0523 |
| C9YY51 | Cystathionine beta-synthase | SCAB_54591 | 0.0596 | 0.0577 | 0.0582 |
| C9ZCM3 | Ornithine aminotransferase | SCAB_78471 (*rocD*) | 0.0487 | 0.0589 | 0.1266 |
| C9YUJ1 | Uncharacterized protein | SCAB_19711 | 0.0412 | 0.1309 | 0.1096 |
| C9Z7H9 | Gamma-glutamyl phosphate reductase | SCAB_60221 (*proA*) | 0.0589 | 0.0634 | 0.0398 |
| A0A100JS54 | Alanine dehydrogenase | SsS58_05086 (*ald_2*) | 0.1313 | 0.0321 | 0.0707 |
| C9ZBY2 | Dipeptidyl-peptidase IV | SCAB_31381 | 0.0448 | 0.0290 | 0.0256 |
| C9Z8G8 | Homoserine dehydrogenase | SCAB_28961 (*thrA*) | 0.0615 | 0.0522 | 0.0390 |
| C9Z3X7 | Peptidase | SCAB_41581 | 0.0463 | 0.0303 | 0.0290 |
| C9Z581 | 2-isopropylmalate synthase | SCAB_26871 | 0.0483 | 0.0322 | 0.0289 |
| C9Z665 | 3-dehydroquinate synthase | SCAB_75121 (*aroB*) | 0.0747 | 0.0483 | 0.0600 |
| C9YTR8 | Probable cytosol aminopeptidase | SCAB_67071 (*pepA*) | 0.0462 | 0.0664 | 0.0596 |
| C9ZGG2 | Uncharacterized protein | SCAB_18021 | 0.0224 | 0.0813 | 0.0625 |
| C9YYA5 | 2,3,4,5-tetrahydropyridine-2,6-dicarboxylate N-succinyltransferase | SCAB_70141 (*dapD*) | 0.0954 | 0.0561 | 0.0429 |
| A0A100JIE1 | Choline dehydrogenase | SsS58_00444 (*betA_1*) | 0.0374 | 0.0393 | 0.0470 |
| C9YZE4 | Urocanate hydratase | SCAB_54701 (*hutU*) | 0.0281 | 0.0270 | 0.0523 |
| C9YVG0 | Histidinol dehydrogenase | SCAB_68351 (*hisD*) | 0.0463 | 0.0367 | 0.0460 |
| C9ZHC6 | Glutamine synthetase | SCAB_66491 | 0.0328 | 0.0294 | 0.0447 |
| C9Z153 | 4-hydroxyphenylpyruvate dioxygenase | SCAB_56371 (*hpd*) | 0.0201 | 0.0376 | 0.0518 |
| C9Z7P2 | ATP phosphoribosyltransferase | SCAB_75651 (*hisG*) | 0.0594 | 0.0642 | 0.0509 |
| C9YTU9 | Anthranilate phosphoribosyltransferase | SCAB_67381 (*trpD1 trpD*) | 0.0653 | 0.0563 | 0.0494 |
| C9Z3Y3 | Aspartokinase | SCAB_41651 (*lysC*) | 0.0473 | 0.0378 | 0.0316 |
| C9ZAF2 | 3-phosphoshikimate 1-carboxyvinyltransferase | SCAB_30421 (*aroA2 aroA*) | 0.0334 | 0.0448 | 0.0273 |
| C9Z781 | Argininosuccinate synthase | SCAB_44121 (*argG*) | 0.0347 | 0.0339 | 0.0174 |
| C9YVH6 | Tryptophan synthase alpha chain | SCAB_68511 (*trpA*) | 0.0664 | 0.0681 | 0.0473 |
| C9Z588 | Branched-chain-amino-acid aminotransferase | SCAB_26941 | 0.0606 | 0.0366 | 0.0288 |
| C9YXU0 | Fumarylacetoacetase | SCAB_37861 | 0.0367 | 0.0328 | 0.0538 |
| C9Z4K2 | N-acetyl-gamma-glutamyl-phosphate reductase | SCAB_74201 (*argC*) | 0.0400 | 0.0442 | 0.0624 |
| C9YVH4 | Indole-3-glycerol phosphate synthase | SCAB_68491 (*trpC1 trpC*) | 0.0875 | 0.0501 | 0.0444 |
| C9ZGV2 | Histidine ammonia- | SCAB_34501 (*hutH*) | 0.0393 | 0.0233 | 0.0424 |
| C9Z8U5 | Probable M18 family aminopeptidase 2 | SCAB_45071 (*apeB*) | 0.0359 | 0.0279 | 0.0236 |
| C9Z7K3 | Carbamoyl-phosphate synthase large chain | SCAB_75231 (*pyrA carB*) | 0.0034 | 0.0122 | 0.0128 |
| C9Z0P6 | Dihydroxy-acid dehydratase | SCAB_39291 (*ilvD*) | 0.0216 | 0.0177 | 0.0167 |
| C9YTX6 | Phospho-2-dehydro-3-deoxyheptonate aldolase | SCAB_67661 (*aroH*) | 0.0280 | 0.0300 | 0.0167 |
| C9ZAP7 | Cystathionine/methionine gamma-synthase/lyase | SCAB_46251 (*cysA*) | 0.0498 | 0.0195 | 0.0352 |
| A0A1M5UHI8 | Sarcosine oxidase subunit beta | SAMN05444521_1159 | 0.0372 | 0.0242 | 0.0255 |
| C9YVI6 | Ferredoxin-dependent glutamate synthase 1 | SCAB_68611 (*gltB*) | 0.0060 | 0.0069 | 0.0071 |
| C9YXA3 | Sarcosine oxidase gamma subunit | SCAB_6921 (*soxG*) | 0.0611 | 0.0605 | 0.0745 |
| A0A100JWN8 | Kynureninase | SsS58_07499 (*kynU*) | 0.0154 | 0.0189 | 0.0523 |
| C9ZBX0 | Aminotransferase | SCAB_31261 | 0.0207 | 0.0329 | 0.0309 |
| C9Z8G6 | Homoserine kinase | SCAB_28941 (*thrB*) | 0.0332 | 0.0305 | 0.0340 |
| C9YY98 | Cysteine desulfurase | SCAB_70071 | 0.0234 | 0.0250 | 0.0164 |
| C9Z443 | Aspartate aminotransferase | SCAB_42271 | 0.0180 | 0.0240 | 0.0186 |
| C9ZGT9 | Cystathionine gamma-synthase | SCAB_34371 (*metB*) | 0.0272 | 0.0228 | 0.0204 |
| C9ZBW7 | Succinyl-diaminopimelate desuccinylase | SCAB_31231 (*dapE*) | 0.0430 | 0.0259 | 0.0176 |
| A0A100JYB2 | Glutamate-pyruvate aminotransferase AlaA | SsS58_08373 (*alaA_2*) | 0.0221 | 0.0261 | 0.0215 |
| A0A124C401 | Phosphoribosyl isomerase A | SsS58_03275 (*priA_1 hisA priA*) | 0.0675 | 0.0316 | 0.0201 |
| C9Z7T1 | Oxidoreductase | SCAB_76051 | 0.0241 | 0.0077 | 0.0179 |
| A0A117EER3 | Argininosuccinate lyase | SsS58_04844 (*argH*) | 0.0166 | 0.0113 | 0.0244 |
| C9YYX3 | Arginine deiminase | SCAB_23151 (*arcA2 arcA*) | 0.0279 | 0.0120 | 0.0373 |
| C9Z7B7 | Aspartate-semialdehyde dehydrogenase | SCAB_59621 (*asd1 asd*) | 0.0041 | 0.0272 | 0.0136 |
| A0A069K7G5 | 4-hydroxy-tetrahydrodipicolinate synthase | DT87_22480 (*dapA*) | 0.0672 | 0.0983 | 0.0754 |
| G2GCN0 | D-3-phosphoglycerate dehydrogenase | SZN_16175 | 0.0671 | 0.0521 | 0.0274 |
| C9Z402 | Aminotransferase | SCAB_41841 | ND^¶^ | 0.0015 | 0.0251 |
| A0A0L0KTF0 | NAD-glutamate dehydrogenase | IQ64_32445 | 0.0300 | 0.0246 | 0.0279 |
| A0A081XSG7 | 3-isopropylmalate dehydrogenase | BU52_13805 (*leuB*) | 0.0689 | 0.0253 | 0.0745 |
| A0A0N0YWX9 | S-adenosylmethionine synthase | ADL27_19595 (*metK*) | 0.0690 | 0.0485 | 0.0233 |
| A0A1Q5KV42 | D-3-phosphoglycerate dehydrogenase | AMK31_15210 | 0.0196 | 0.0353 | 0.0350 |
| A0A177HGG8 | NAD-specific glutamate dehydrogenase | STSP_65790 (*gdhB_3*) | 0.0058 | 0.0104 | 0.0045 |
| A0A1X2MQU4 | Serine hydroxymethyltransferase | B5181_17290 (*glyA*) | 0.0441 | 0.0809 | 0.0748 |
| L8P9A6 | Glutamine synthetase | STVIR_6340 | 0.1467 | 0.1031 | ND |
| A0A0L8MVR0 | Tryptophan synthase beta chain | ADK75_14015 (*trpB*) | 0.0102 | 0.0367 | 0.0316 |
| A0A143C079 | Aminopeptidase N | A4E84_13905 | ND | 0.0106 | 0.0106 |
| A0A1J4NZU0 | Aminotransferase | WN71_013225 | 0.0398 | 0.0229 | ND |
| C9ZCE7 | Cyclase | SCAB_63371 | 0.3068 | 0.2366 | 0.2706 |
| C9YXA7 | Dehydrogenase | SCAB_6961 | 0.0719 | 0.0711 | 0.0971 |
| C9ZBY9 | Transport system extracellular solute-binding protein | SCAB_31461 | 0.5808 | 0.2900 | 0.3169 |
| C9Z204 | Glutamate uptake system binding subunit | SCAB_24891 (*gluB*) | 0.4141 | 0.6436 | 0.7274 |
| C9YXA8 | Glycine betaine-binding lipoprotein | SCAB_6971 | 0.1731 | 0.1003 | 0.1041 |
| C9Z0P2 | Glutamate uptake system ATP-binding subunit | SCAB_24881 (*gluA*) | 0.1956 | 0.1285 | 0.1806 |
| C9ZBZ2 | Peptide ABC transporter | SCAB_31501 | 0.1062 | 0.0757 | 0.0708 |
| C9YWN7 | ABC transporter ATP-binding subunit | SCAB_68901 | 0.0947 | 0.0487 | 0.0846 |
| C9ZA98 | Polar amino acid transport system | SCAB_29881 (*atrA*) | 0.1055 | 0.0465 | 0.0626 |
| C9YZP9 | Secreted solute-binding protein | SCAB_70761 | 0.0586 | 0.0760 | 0.0763 |
| C9Z206 | Glutamate uptake system | SCAB_24911 (*gluD*) | 0.0777 | 0.0454 | 0.0600 |
| C9YXB0 | Glycine betaine ABC transport system ATP-binding protein | SCAB_6991 | 0.0599 | 0.0282 | 0.0400 |
| A0A1S2K4L9 | Branched chain amino acid ABC transporter substrate-binding protein | BJP40_29165 | 0.0614 | 0.0290 | 0.0355 |
| C9YWN6 | ABC transporter ATP-binding subunit | SCAB_68891 | 0.1049 | 0.0660 | 0.0330 |
| C9Z5D2 | Oligopeptide ABC transporter component | SCAB_27391 | 0.0714 | 0.0243 | 0.0298 |
| A0A0M8UZZ0 | Glutamate-binding protein | ADL00_30255 | 0.1364 | 0.1783 | 0.2254 |
| A0A101URN4 | Glutamate ABC transporter ATP-binding protein | AQJ91_40805 | 0.0241 | 0.0690 | 0.0771 |
| C9Z7M2 | Secreted substrate-binding protein (Transport system associated) | SCAB_75441 | 0.0239 | 0.0699 | 0.0533 |
| Carbohydrate metabolism | | | | | |
| A0A101PMD4 | Transaldolase | AQI96_06170 (*tal*) | 0.0186 | 0.1784 | 0.1332 |
| A0A101SS39 | Fructose-bisphosphate aldolase | AQJ64_29310 | 0.0437 | 0.2113 | 0.0317 |
| A0A1Q5LYT5 | Glucoamylase | AMK09_36290 | ND | 0.0236 | 0.0200 |
| A0A0M8WDG6 | Transaldolase | ADL01_09595 (*tal*) | ND | 0.0403 | 0.1003 |
| A0A117ECF6 | Malate dehydrogenase | SsS58_01230 (*mdh*) | 0.4585 | 0.4650 | 0.7118 |
| C9YY62 | Glyceraldehyde-3-phosphate dehydrogenase | SCAB_69701 (*gap*) | 0.4801 | 0.3837 | 0.2622 |
| C9YU13 | Pyruvate carboxylase | SCAB_81861 | 0.0909 | 0.0957 | 0.0968 |
| C9YY67 | Glucose-6-phosphate isomerase | SCAB_69751 (*pgi2 pgi*) | 0.1897 | 0.1997 | 0.2146 |
| C9Z6V6 | Glycogen phosphorylase | SCAB_27951 (*glgP*) | 0.0853 | 0.1244 | 0.1218 |
| C9YY84 | Transketolase | SCAB_69921 (*tktA*) | 0.1585 | 0.1260 | 0.1477 |
| C9ZAZ8 | Secreted protein | SCAB_77201 | 0.0376 | 0.0749 | 0.2157 |
| C9Z433 | Fructose-bisphosphate aldolase | SCAB_42161 (*fba*) | 0.3057 | 0.3760 | 0.1962 |
| C9Z545 | 6-phosphogluconate dehydrogenase, decarboxylating | SCAB_11811 | 0.1961 | 0.1963 | 0.1919 |
| C9YY63 | Phosphoglycerate kinase | SCAB_69711 (*pgk*) | 0.2501 | 0.2187 | 0.1582 |
| C9Z5R8 | Citrate synthase | SCAB_58791 (*citA*) | 0.2520 | 0.1496 | 0.1914 |
| C9YY83 | Transaldolase | SCAB_69911 (*tal tal2*) | 0.2536 | 0.2348 | 0.2177 |
| C9YUT7 | Succinate--CoA ligase [ADP-forming] subunit beta | SCAB_35801 (*sucC*) | 0.1991 | 0.1863 | 0.1366 |
| C9YWG2 | UTP--glucose-1-phosphate uridylyltransferase | SCAB_53131 (*gtaB*) | 0.1903 | 0.1903 | 0.1767 |
| C9YY64 | Triosephosphate isomerase | SCAB_69721 (*tpiA*) | 0.2169 | 0.2207 | 0.2015 |
| A0A100JT13 | 4-aminobutyrate aminotransferase GabT | SsS58_05564 (*gabT*) | 0.1355 | 0.1596 | 0.2122 |
| C9Z6Y3 | Pyruvate kinase | SCAB_28241 (*pyk2*) | 0.1513 | 0.0881 | 0.0827 |
| C9YTC3 | Succinate dehydrogenase flavoprotein subunit | SCAB_35291 (*dhsA*) | 0.1283 | 0.0989 | 0.0907 |
| C9YUT6 | Succinate--CoA ligase [ADP-forming] subunit alpha | SCAB_35791 (*sucD*) | 0.2140 | 0.1778 | 0.1644 |
| C9YY37 | Enolase | SCAB_54441 (*eno*) | 0.1235 | 0.1167 | 0.1056 |
| C9YY82 | Glucose-6-phosphate 1-dehydrogenase | SCAB_69901 (*zwf*) | 0.0939 | 0.0835 | 0.0997 |
| C9YX58 | Phosphoglucomutase | SCAB_6461 (*pgm*) | 0.0966 | 0.0696 | 0.0723 |
| C9Z6V8 | Alpha-1.4-glucan:maltose-1-phosphate maltosyltransferase | SCAB_27981 (*pep1A glgE*) | 0.0695 | 0.0708 | 0.0567 |
| C9ZBJ5 | alpha-mannosidase | SCAB_15481 | 0.0238 | 0.0380 | 0.0613 |
| C9ZAM6 | 1L-myo-inositol-1-phosphate synthase | SCAB_46011 (*ino1*) | 0.1407 | 0.0768 | 0.0735 |
| C9ZGJ3 | Malate synthase | SCAB_ (*aceB1*) | 0.0847 | 0.0391 | 0.0627 |
| C9Z5Q8 | Ribokinase | SCAB_58701 (*rbsK*) | 0.1182 | 0.1399 | 0.1373 |
| C9ZFY5 | Xylose isomerase | SCAB_79861 (*xylA*) | 0.0535 | 0.1188 | 0.1368 |
| C9Z1A6 | Glycosyl hydrolase | SCAB_71771 | 0.0438 | 0.0456 | 0.0506 |
| C9Z462 | Glutamine--fructose-6-phosphate aminotransferase [isomerizing] | SCAB_57861 (*glmS glmS2*) | 0.0366 | 0.0554 | 0.0539 |
| C9Z6W1 | 1,4-alpha-glucan branching enzyme GlgB | SCAB_28011 (*glgB1 glgB*) | 0.0326 | 0.0286 | 0.0372 |
| C9ZBM1 | Uncharacterized protein | SCAB_15741 | 0.0724 | 0.0747 | 0.0640 |
| C9ZH18 | 2,3-bisphosphoglycerate-dependent phosphoglycerate mutase | SCAB_49941 (*gpmA*) | 0.1225 | 0.0998 | 0.0925 |
| C9YW22 | Carbohydrate epimerase | SCAB_21461 | 0.0374 | 0.0679 | 0.0628 |
| C9YXN7 | Rhamnosidase | SCAB_22771 | ND | 0.0268 | 0.0461 |
| C9YXR3 | Oxidoreductase | SCAB_37561 | 0.0098 | 0.0385 | 0.0279 |
| A0A086GSJ1 | Glucose-1-phosphate adenylyltransferase | IQ62_25440 (*glgC*) | 0.0633 | 0.0433 | 0.0584 |
| C9ZGR4 | Phosphoenolpyruvate carboxykinase [GTP] | SCAB_34111 (*pckG*) | 0.0066 | 0.0326 | 0.0780 |
| C9YW45 | Phosphoglucosamine mutase | SCAB_36611 (*glmM*) | 0.0465 | 0.0460 | 0.0332 |
| C9ZD79 | DNA-binding protein | SCAB_16731 | 0.0116 | 0.0745 | 0.1112 |
| C9YUM4 | Malto-oligosyltrehalose trehalohydrolase | SCAB_20081 | 0.0237 | 0.0243 | 0.0375 |
| C9YSV0 | Probable phosphoketolase | SCAB_4201 | 0.0388 | 0.0159 | 0.0156 |
| C9YY80 | 6-phosphogluconolactonase | SCAB_69881 (*pgl*) | 0.0632 | 0.0648 | 0.0959 |
| C9Z9C4 | Mannose-1-phosphate guanyltransferase | SCAB_76231 | 0.0047 | 0.0218 | 0.0179 |
| C9YXN3 | Glycosyl hydrolase | SCAB_22731 | ND | 0.0077 | 0.0469 |
| C9ZH59 | Trehalose-phosphate synthase | SCAB_50381 | 0.0314 | 0.0235 | 0.0313 |
| C9ZBM2 | 6-phosphogluconate dehydrogenase | SCAB_15751 | 0.0623 | 0.0551 | 0.0414 |
| A0A0Q8YR68 | Malate dehydrogenase | ASE41_08580 (*mdh*) | 0.3357 | 0.3351 | 0.5115 |
| C9ZF65 | Fructose-1.6-bisphosphatase | SCAB_33521 | 0.0420 | 0.0454 | 0.0439 |
| C9Z737 | Secreted protein | SCAB_43661 | 0.0364 | 0.0134 | 0.0152 |
| C9ZBM0 | Glucose-6-phosphate 1-dehydrogenase | SCAB_15731 (*zwf*) | 0.0736 | 0.0273 | 0.0640 |
| C9ZFY4 | Xylulose kinase | SCAB_79851 (*xylB*) | 0.0304 | 0.0216 | 0.0218 |
| C9Z7D9 | Sugar-phosphate isomerase | SCAB_59851 | 0.0885 | 0.0641 | 0.0377 |
| C9YT49 | Oxidoreductase | SCAB_19421 | 0.0213 | 0.0289 | 0.0446 |
| A0A086GWQ1 | Polyphosphate glucokinase | IQ62_16835 | 0.0733 | 0.0429 | 0.0569 |
| C9Z0H2 | Inositol-1-monophosphatase | SCAB_24101 | 0.0390 | 0.0421 | 0.0317 |
| C9Z5T1 | Thiamine pyrophosphate-dependent protein | SCAB_58921 | 0.0218 | 0.0085 | 0.0185 |
| C9YZ87 | Bisphosphoglycerate mutase | SCAB_38701 | 0.0528 | 0.0479 | 0.0396 |
| C9Z1A1 | NAD kinase | SCAB_71721 (*ppnK2 nadK*) | 0.0427 | 0.0270 | 0.0209 |
| C9Z6W0 | Conserved hypothetical Pep2A protein | SCAB_28001 (*pep2A*) | 0.0240 | 0.0205 | 0.0136 |
| C9ZGI1 | Dehydrogenase | SCAB_18231 | 0.0230 | 0.0280 | 0.0209 |
| C9Z1V1 | Sugar isomerase | SCAB_9441 | ND | 0.0243 | 0.0619 |
| C9Z2P0 | NagD-like phosphatase | SCAB_57241 | 0.0369 | 0.0497 | 0.0252 |
| C9Z507 | Secreted hydrolase | SCAB_11431 | 0.0066 | 0.0012 | 0.0098 |
| A0A0U3NKZ9 | Pyruvate carboxylase | AS200_37785 | 0.0353 | 0.0208 | 0.0358 |
| C9YSZ3 | levansucrase | SCAB_18831 | ND | 0.0207 | 0.0206 |
| L1KZR8 | 1,4-alpha-glucan branching enzyme GlgB | STRIP9103_06855 (*glgB*) | 0.0091 | 0.0052 | 0.0145 |
| A0A1M5ZUR4 | Triosephosphate isomerase | SAMN05444521_6810 (*tpiA*) | 0.1273 | 0.1730 | 0.1527 |
| C9YUA4 | Glyceraldehyde-3-phosphate dehydrogenase | SCAB_4751 (*gap*) | 0.0467 | 0.0062 | 0.0024 |
| A0A0Q6X6P7 | Glucose-6-phosphate isomerase | ASD08_19530 (*pgi*) | 0.0433 | 0.0442 | 0.0781 |
| A0A1Q5M2S2 | Transketolase | AMK26_05785 (*pgi*) | 0.0507 | 0.0384 | 0.0201 |
| A0A081XI56 | Glucoamylase | BU52_31710 | ND | 0.0182 | 0.0162 |
| A0A0N0T1D6 | Malate dehydrogenase | ADK64_10185 (*mdh*) | 0.0871 | 0.1340 | 0.1968 |
| A0A0T1UG03 | Fructose-bisphosphate aldolase | ASD48_31890 | ND | 0.0450 | ND |
| D6K183 | Pyruvate kinase | SSTG_03457 | 0.0159 | 0.0099 | 0.0213 |
| A0A117P846 | Malate dehydrogenase | AQI70_18390 (*mdh*) | 0.0828 | 0.0233 | 0.2200 |
| A0A089X9G2 | Phosphoglycerate kinase | SGLAU_08735 (*pgk*) | 0.0617 | 0.0222 | ND |
| A0A170XU30 | Malate dehydrogenase | STXM2123_2743 (*mdh*) | ND | 0.0441 | 0.0674 |
| C9Z540 | Glucose-1-phosphate adenylyltransferase | SCAB_11761 (*glgC*) | ND | 0.0318 | 0.0175 |
| A0A1Q5KMA5 | Transaldolase | AMK31_21960 (*tal*) | 0.0263 | 0.0245 | ND |
| C9Z4A2 | AP_endonuc_2 domain-containing protein | SCAB_58301 (*tal*) | 0.1062 | 0.0869 | 0.1040 |
| C9ZHD5 | Secreted maltose-binding protein | SCAB_66581 (*malE*) | 0.9859 | 1.0932 | 0.8240 |
| C9YVX8 | Secreted solute-binding protein | SCAB_21021 | 0.6271 | 0.4377 | 0.2891 |
| C9ZD81 | Secreted solute binding protein | SCAB_16751 | 0.0824 | 0.3804 | 0.4608 |
| C9ZH37 | Maltose/cellubiose ABC transporter ATP-binding subunit | SCAB_50161 (*msiK*) | 0.2516 | 0.1930 | 0.1212 |
| C9YT14 | Probable solute-binding lipoprotein | SCAB_19051 | 0.1730 | 0.1093 | 0.1306 |
| C9YUG2 | Substrate-binding component of ABC transporter | SCAB_5351 | 0.0854 | 0.1720 | 0.1436 |
| C9Z9C1 | Phosphoenolpyruvate-protein phosphotransferase | SCAB_76201 | 0.0850 | 0.0484 | 0.0610 |
| C9YT90 | Sugar ABC transporter ATP-binding subunit | SCAB_34961 | 0.0561 | 0.0666 | 0.0657 |
| C9YVY2 | Extracellular solute-binding receptor | SCAB_21061 | 0.0505 | 0.0584 | 0.0918 |
| C9YYU9 | Substrate-binding component of ABC transporter | SCAB_8191 | 0.0464 | 0.0681 | 0.0744 |
| C9Z2T0 | Secreted solute-binding protein (Transport system associated) | SCAB_57661 | 0.0718 | 0.0535 | 0.0443 |
| C9ZHD7 | Maltose transporter | SCAB_66601 (*malG*) | 0.0840 | 0.0647 | 0.0669 |
| C9Z1U7 | Secreted solute-binding lipoprotein | SCAB_9401 | 0.0035 | 0.0678 | 0.0472 |
| C9ZAA7 | Extracellular substrate-binding protein | SCAB_29961 | ND | 0.0694 | 0.1002 |
| C9Z451 | Eecreted cellobiose-binding (Transport system associated) | SCAB_57751 | 0.0191 | 0.0347 | 0.0639 |
| C9ZDW4 | Secreted transport-associated protein | SCAB_63891 | 0.0148 | 0.0333 | 0.0397 |
| C9YYL7 | Substrate-binding transport lipoprotein | SCAB_7331 | 0.0027 | 0.0590 | 0.0434 |
| C9Z307 | Glycerol uptake facilitator protein | SCAB_73301 (*glpF*) | 0.0738 | 0.0533 | 0.0680 |
| C9YT89 | Sugar ABC transporter | SCAB_34951 | 0.0207 | 0.0318 | 0.0290 |
| C9ZDX0 | Secreted solute-binding protein | SCAB_63951 | 0.0104 | 0.0412 | 0.0215 |
| M3DFW3 | MalE protein | SBD_2895 | 0.6213 | 0.6566 | 0.5111 |
| C9Z619 | Extracellular solute-binding lipoprotein | SCAB_74641 | ND | 0.0087 | 0.0457 |
| A0A1Q4WEP6 | Sugar ABC transporter ATP-binding protein | AMK13_08585 | 0.0564 | 0.0744 | 0.0365 |
| A0A117EFR4 | Phosphoenolpyruvate-protein phosphotransferase | SsS58_06401 (*pstI*) | 0.0424 | 0.0311 | 0.0600 |
| A0A1S2PW04 | Sugar ABC transporter substrate-binding protein | BIV24_06555 | 0.0949 | 0.1587 | ND |
| A0A0U3Q3H2 | ABC transporter substrate-binding protein | AS200_12400 | 0.1575 | 0.0330 | 0.0384 |
| L1KT66 | D-xylose ABC transporter. periplasmic D-xylose-binding family protein | STRIP9103_07714 | 0.1699 | 0.0155 | 0.0384 |
| A0A1D2IGZ0 | Maltodextrin-binding protein MdxE | APS67_001960 (*mdxE*) | 0.1587 | 0.0335 | ND |
| A0A0L8L2H4 | Phosphoenolpyruvate-protein phosphotransferase | ADK37_28190 AQJ84_24530 | ND | 0.0149 | 0.0335 |
| C9Z9C8 | Glycogen regulation protein GarA | SCAB_76271 | 0.0851 | 0.0794 | 0.2494 |
| Cell cycle control, cell division, chromosome partitioning | | | | | |
| C9ZAL5 | Chromosome partitioning nuclease | SCAB_45901 (*parB*) | 0.0231 | 0.0613 | 0.0406 |
| C9YZX7 | Sporulation protein | SCAB_71571 | 0.0861 | 0.0959 | 0.0683 |
| C9Z7E9 | Trigger factor | SCAB_59931 (*tig*) | 0.1568 | 0.1124 | 0.0810 |
| C9YVC9 | Cell division protein FtsZ | SCAB_68031 (*ftsZ*) | 0.0370 | 0.0435 | 0.0429 |
| C9Z7F9 | Cell shape-determining protein MreB | SCAB_60021 (*mreB*) | 0.0539 | 0.0512 | 0.0336 |
| A0A1Q5KFF9 | Cell division protein DivIVA | AMK31_24410 | 0.0626 | 0.0486 | 0.0319 |
| C9Z1B1 | Sporulation protein | SCAB_71841 | 0.0392 | 0.0260 | 0.0300 |
| C9Z244 | AAA_31 domain-containing protein | SCAB_25291 | 0.0184 | 0.0219 | 0.0109 |
| Cell wall/membrane/envelope biogenesis | | | | | |
| C9Z994 | Tricorn protease homolog | SCAB_61611 (*tri1*) | 0.1759 | 0.1193 | 0.1494 |
| C9YT92 | Lipoprotein | SCAB_34981 | 0.3756 | 0.4899 | 0.6166 |
| C9Z8V2 | D-alanyl-D-alanine carboxypeptidase | SCAB_45141 | 0.2023 | 0.3218 | 0.3690 |
| C9Z836 | Nucleotide sugar-1-phosphate transferase | SCAB_13061 | 0.2026 | 0.1434 | 0.0770 |
| C9YY13 | Bifunctional protein GlmU | SCAB_54171 (*glmU*) | 0.0719 | 0.0527 | 0.0564 |
| C9ZAN1 | Uncharacterized protein | SCAB_46071 | 0.0652 | 0.0384 | 0.0522 |
| C9ZAN0 | Ala_racemase_N domain-containing protein | SCAB_46061 | 0.0810 | 0.0571 | 0.0593 |
| C9Z111 | UDP-N-acetylglucosamine 1-carboxyvinyltransferase | SCAB_55951 (*murA*) | 0.0500 | 0.0325 | 0.0515 |
| C9YVC3 | UDP-N-acetylmuramoyl-tripeptide--D-alanyl-D-alanine ligase | SCAB_67971 (*murF*) | 0.0321 | 0.0306 | 0.0342 |
| C9YYZ4 | Glycosyl transferase | SCAB_23361 | 0.0445 | 0.0365 | 0.0578 |
| C9ZDW7 | Dehydratase | SCAB_63921 | 0.0346 | 0.0392 | 0.0454 |
| C9YZ11 | dTDP-4-dehydrorhamnose 3.5-epimerase | SCAB_23531 | 0.0768 | 0.0737 | 0.0907 |
| C9YUS1 | UDP-N-acetylmuramate--L-alanine ligase | SCAB_20561 (*murC*) | 0.0285 | 0.0267 | 0.0244 |
| C9YZ06 | Nucleotide sugar epimerase/dehydratase | SCAB_23481 | 0.0469 | 0.0370 | 0.0401 |
| C9ZCN6 | Ligase | SCAB_78601 | 0.0420 | 0.0362 | 0.0242 |
| C9YW21 | Pyruvyl-transferase | SCAB_21451 | 0.0157 | 0.0351 | 0.0233 |
| C9ZBQ4 | Membrane protein | SCAB_30571 | 0.0347 | 0.0249 | 0.0336 |
| C9YVC2 | UDP-N-acetylmuramoyl-L-alanyl-D-glutamate--2.6-diaminopimelate ligase | SCAB_67961 (*murE*) | 0.0285 | 0.0087 | 0.0107 |
| C9Z722 | Carboxypeptidase | SCAB_43501 | 0.0490 | 0.0044 | 0.0176 |
| A0A1I2UI89 | Tricorn protease homolog | SAMN02787118_12824 | ND | 0.0250 | 0.0241 |
| C9ZBT1 | Reductase | SCAB_30871 | 0.0435 | 0.0683 | 0.0565 |
| Coenzyme transport and metabolism | | | | | |
| A0A100JWI2 | Glutamate--tRNA ligase | SsS58_07425 (*gltX*) | 0.1588 | 0.0917 | 0.1066 |
| C9Z1Y4 | Probable cobalamin biosynthesis protein cobN | SCAB_9771 (*cobN*) | 0.0417 | 0.0341 | 0.0407 |
| C9Z663 | Chorismate synthase | SCAB_75101 (*aroC*) | 0.1057 | 0.0999 | 0.0913 |
| C9YXA6 | Bifunctional protein FolD | SCAB_6951 (*folD*) | 0.1722 | 0.1243 | 0.1405 |
| C9Z3L8 | Adenosine/AMP deaminase | SCAB_25801 | 0.0831 | 0.0722 | 0.0591 |
| A0A100JRK2 | Pyridoxal 5'-phosphate synthase subunit PdxS | SsS58_04787 (*pdxS*) | 0.0617 | 0.0764 | 0.0691 |
| C9YUS8 | Bifunctional protein FolD | SCAB_35711 (*folD*) | 0.0575 | 0.0819 | 0.0693 |
| C9YXR6 | NAD(P)-bd_dom domain-containing protein | SCAB_37591 | 0.0629 | 0.0897 | 0.0519 |
| C9Z7P0 | 6,7-dimethyl-8-ribityllumazine synthase | SCAB_75631 (*ribH*) | 0.1490 | 0.1065 | 0.1684 |
| C9YZ58 | 3-octaprenyl-4-hydroxybenzoate carboxy-lyase | SCAB_38401 | 0.0482 | 0.0373 | 0.0477 |
| C9Z7F6 | Folylpolyglutamate synthase | SCAB_59991 (*fpgS*) | 0.0402 | 0.0283 | 0.0345 |
| C9YTS3 | Nicotinate-nucleotide--dimethylbenzimidazole phosphoribosyltransferase | SCAB_67121 (*cobT*) | 0.0378 | 0.0486 | 0.0459 |
| C9Z7K9 | Coenzyme A biosynthesis bifunctional protein CoaBC | SCAB_75291 (*cobT*) | 0.0424 | 0.0251 | 0.0386 |
| C9YZ88 | Glutamate-1-semialdehyde 2,1-aminomutase | SCAB_38711 (*hemL*) | 0.0315 | 0.0269 | 0.0305 |
| C9Z603 | Uroporphyrin-III methyltransferase | SCAB_74471 | 0.0436 | 0.0228 | 0.0274 |
| C9ZEC7 | Thiamine biosynthesis protein ThiF | SCAB_79581 | 0.0258 | 0.0146 | 0.0121 |
| C9Z3M4 | Aminotransferase | SCAB_25861 | 0.0111 | 0.0278 | 0.0408 |
| C9YWG3 | Molybdopterin molybdenumtransferase | SCAB_53141 (*moeA*) | 0.0188 | 0.0282 | 0.0175 |
| C9YXW8 | Demethylmenaquinone methyltransferase | SCAB_38161 (*menG*) | 0.0363 | 0.0332 | 0.0290 |
| C9ZBZ9 | Phosphomethylpyrimidine synthase | SCAB_46341 (*thiC*) | 0.0020 | 0.0150 | 0.0125 |
| C9YVW2 | Uroporphyrinogen decarboxylase | SCAB_20861 (*hemE*) | 0.0191 | 0.0353 | 0.0097 |
| A0A086GPI2 | Uroporphyrin-III C-methyltransferase | IQ62_31540 | 0.0318 | 0.0201 | 0.0195 |
| Energy production and conversion | | | | | |
| C9YTR7 | Dihydrolipoyl dehydrogenase | SCAB_67061 | 0.4399 | 0.5623 | 0.7618 |
| C9ZFY0 | Aldehyde dehydrogenase | SCAB_79811 (*thcA*) | 0.3607 | 0.5644 | 0.4464 |
| C9Z6J6 | Isocitrate dehydrogenase [NADP] | SCAB_12211 (*idh*) | 0.2877 | 0.2070 | 0.2381 |
| C9ZA89 | 2-oxoglutarate dehydrogenase | SCAB_29781 | 0.1325 | 0.1260 | 0.1226 |
| C9Z8F0 | ATP synthase subunit alpha | SCAB_28781 (*atpA*) | 0.3670 | 0.2955 | 0.2953 |
| C9YVY6 | Aconitate hydratase | SCAB_21101 (*sacA*) | 0.1846 | 0.1504 | 0.1573 |
| C9Z8E8 | ATP synthase subunit beta | SCAB_28761 (*atpD*) | 0.3723 | 0.3697 | 0.3249 |
| C9ZE04 | Pyruvate dehydrogenase E1 component | SCAB_64301 | 0.1397 | 0.1148 | 0.1283 |
| C9YXV6 | NADH-quinone oxidoreductase | SCAB_38031 (*nuoG*) | 0.1515 | 0.1412 | 0.1309 |
| C9YTR6 | Dihydrolipoamide acetyltransferase component of pyruvate dehydrogenase complex | SCAB_67051 | 0.2110 | 0.2088 | 0.1703 |
| C9YZL4 | NAD-glutamate dehydrogenase | SCAB_55421 (*gdh*) | 0.0442 | 0.0401 | 0.0393 |
| C9Z1J4 | L-glyceraldehyde 3-phosphate reductase | SCAB_85321 | 0.4297 | 0.2596 | 0.1836 |
| C9Z306 | Glycerol kinase | SCAB_73291 (*glpK1 glpK*) | 0.1735 | 0.1593 | 0.1832 |
| C9YUX5 | IMP dehydrogenase/ GMP reductase | SCAB_36201 | 0.2111 | 0.1627 | 0.1991 |
| C9Z098 | Oxidoreductase | SCAB_8471 | 0.1690 | 0.1485 | 0.1854 |
| C9YTU0 | Cytochrome c oxidase subunit II | SCAB_67291 (*ctaC*) | 0.1466 | 0.1549 | 0.2399 |
| C9ZGW6 | Oxidoreductase | SCAB_34651 | 0.1079 | 0.1167 | 0.1408 |
| C9ZAG4 | Aldehyde dehydrogenase | SCAB_45351 | 0.1191 | 0.0607 | 0.0902 |
| C9Z8E9 | ATP synthase gamma chain | SCAB_28771 (*atpG*) | 0.1706 | 0.1570 | 0.1775 |
| C9YXV7 | NADH-quinone oxidoreductase subunit F | SCAB_38041 (*nuoF*) | 0.0982 | 0.0834 | 0.0930 |
| C9ZGY7 | Sulfurtransferase | SCAB_49631 | 0.1454 | 0.1280 | 0.1010 |
| C9Z8F1 | ATP synthase subunit delta | SCAB_28791 (*atpH*) | 0.2218 | 0.1460 | 0.2083 |
| C9YY08 | Phosphoenolpyruvate carboxylase | SCAB_54111 (*ppc*) | 0.0385 | 0.0348 | 0.0345 |
| C9YWW6 | Electron transfer flavoprotein, alpha subunit | SCAB_82681 | 0.0951 | 0.1153 | 0.0889 |
| A0A100JQ69 | NAD-dependent malic enzyme | SsS58_03984 | 0.0821 | 0.0736 | 0.0631 |
| C9Z5T2 | Methylmalonic acid semialdehyde dehydrogenase | SCAB_58931 (*msdA*) | 0.0507 | 0.0509 | 0.0595 |
| C9YYW7 | Carbonic anhydrase | SCAB_23091 | 0.1700 | 0.0792 | 0.0598 |
| C9Z2E3 | K(+)-insensitive pyrophosphate-energized proton pump | SCAB_40831 (*hppA*) | 0.0424 | 0.0515 | 0.0748 |
| C9Z770 | Protein-lysine 6-oxidase | SCAB_44001 | 0.0864 | 0.0360 | 0.0186 |
| C9Z4G9 | Aldehyde dehydrogenase | SCAB_73861 | 0.0848 | 0.0497 | 0.0651 |
| C9ZDZ7 | Aldo/keto reductase | SCAB_64231 | 0.1394 | 0.0579 | 0.0325 |
| C9ZFK6 | Polyphosphate kinase | SCAB_49421 (*ppk*) | 0.0252 | 0.0249 | 0.0273 |
| C9YTU7 | Ubiquinol-cytochrome c reductase iron-sulfur subunit | SCAB_67361 (*qcrA*) | 0.0933 | 0.0641 | 0.0819 |
| A0A124C4M3 | NADP-dependent oxidoreductase YfmJ | SsS58_05317 (*yfmJ_2*) | 0.0756 | 0.1003 | 0.0762 |
| C9YTM7 | Citrate synthase | SCAB_51341 | 0.0693 | 0.0764 | 0.0850 |
| C9YXV8 | NADH dehydrogenase subunit NuoE | SCAB_38051 (*nuoE*) | 0.0850 | 0.0863 | 0.0840 |
| C9ZFH8 | Disulfide oxidoreductase | SCAB_49141 | 0.0259 | 0.0498 | 0.0606 |
| C9YYR1 | Aldo/keto reductase | SCAB_7801 | 0.0971 | 0.0623 | 0.0438 |
| C9Z8W1 | Quinone oxidoreductase | SCAB_45231 | 0.0666 | 0.0645 | 0.0657 |
| C9Z4X4 | Probable oxidoreductase | SCAB_11101 | 0.0440 | 0.0404 | 0.0351 |
| C9Z8F2 | ATP synthase subunit b | SCAB_28801 (*atpF*) | 0.1692 | 0.0971 | 0.1407 |
| C9Z3M2 | Aldehyde dehydrogenase | SCAB_25841 | 0.0399 | 0.0394 | 0.0374 |
| A0A117EGN5 | Fumarate hydratase class I | SsS58_07900 (*fumA*) | 0.0256 | 0.0288 | 0.0307 |
| C9YTJ5 | Oxidoreductase | SCAB_51011 | 0.0823 | 0.0560 | 0.0759 |
| C9YTR4 | Pyruvate dehydrogenase E1 component | SCAB_67031 | ND | 0.0407 | 0.0605 |
| A0A100JIY1 | Lactate 2-monooxygenase | SsS58_00622 | 0.0445 | 0.0544 | 0.0611 |
| C9YXV4 | NADH-quinone oxidoreductase subunit I | SCAB_38011 (*nuoI*) | 0.1204 | 0.0709 | 0.0503 |
| C9YTU1 | Cytochrome c oxidase subunit 1 | SCAB_67301 (*ctaD1*) | 0.0248 | 0.0348 | 0.0405 |
| C9ZEP5 | Oxidoreductase | SCAB_16961 | 0.0653 | 0.0543 | 0.0609 |
| C9Z7C6 | DSBA domain-containing protein | SCAB_59711 | 0.0591 | 0.0709 | 0.0811 |
| C9Z2X1 | FAD-binding protein | SCAB_72911 | 0.0250 | 0.0320 | 0.0229 |
| A0A0U3PWC9 | Dihydrolipoyl dehydrogenase | ASR50_10625 | 0.2039 | 0.2470 | 0.3069 |
| C9YZN8 | Aldehyde dehydrogenase | SCAB_70651 | 0.0160 | 0.0444 | 0.0292 |
| C9ZGF8 | Oxidoreductase | SCAB_17981 | 0.0566 | 0.0434 | 0.0500 |
| C9Z0H5 | FAD-dependent oxidoreductase | SCAB_24131 | 0.0451 | 0.0252 | 0.0343 |
| C9YXU9 | NADH-quinone oxidoreductase subunit N | SCAB_37961 (*nuoN*) | 0.0249 | 0.0246 | 0.0452 |
| C9Z0A0 | Aldehyde dehydrogenase family protein | SCAB_8501 | 0.0211 | 0.0219 | 0.0390 |
| C9Z990 | Oxidoreductase | SCAB_61571 | 0.0253 | 0.0375 | 0.0446 |
| C9Z2U3 | Aldehyde dehydrogenase | SCAB_72631 | 0.0319 | 0.0421 | 0.0231 |
| A0A1H8NBM4 | Dihydrolipoyl dehydrogenase | SAMN05216267_1021140 | 0.1559 | 0.1885 | 0.2256 |
| C9YXR4 | Oxidoreductase | SCAB_37571 | 0.0187 | 0.0300 | 0.0490 |
| C9YTU8 | Cytochrome B subunit | SCAB_67371 (*qcrB*) | 0.0283 | 0.0260 | 0.0303 |
| A0A1R1SHN3 | Dihydrolipoyl dehydrogenase | SPAR_19328 | 0.1497 | 0.1608 | 0.1967 |
| C9Z9A9 | Betaine aldehyde dehydrogenase | SCAB_76071 (*gbsA*) | 0.0510 | 0.0274 | 0.0390 |
| C9Z278 | Aldehyde dehydrogenase | SCAB_25651 | 0.0248 | 0.0369 | 0.0131 |
| C9YXV5 | NADH-quinone oxidoreductase subunit H | SCAB_38021 (*nuoH*) | 0.0176 | 0.0225 | 0.0322 |
| C9YUW4 | Aldehyde dehydrogenase family protein | SCAB_36091 | 0.0117 | 0.0193 | 0.0213 |
| C9Z2U4 | Zinc-binding alcohol dehydrogenase | SCAB_72641 | 0.0187 | 0.0272 | 0.0381 |
| C9Z8V6 | E1-alpha branched-chain alpha keto acid dehydrogenase system | SCAB_45181 (*bkdF*) | 0.0300 | 0.0333 | 0.0433 |
| C9Z6Y2 | Acetate kinase | SCAB_28231 (*ackA*) | 0.0188 | 0.0226 | 0.0347 |
| A0A170WG78 | Dihydrolipoyl dehydrogenase | STXM2123_1753 | 0.1986 | 0.2493 | 0.3282 |
| C9Z119 | Iron-suplhur oxidoreductase | SCAB_56041 | 0.0174 | 0.0148 | 0.0120 |
| A0A1V4AAH1 | Isocitrate dehydrogenase [NADP] | B1H18_12270 | 0.0657 | 0.0249 | 0.0464 |
| C9Z4N1 | Oxidoreductase F420-dependent glucose-6-phosphate dehydrogenase | SCAB_87211 | 0.0135 | 0.0335 | 0.0374 |
| C9YXV0 | NADH dehydrogenase subunit NuoM | SCAB_37971 (*nuoM*) | 0.0136 | 0.0390 | 0.0398 |
| A0A100JWB2 | Aldehyde dehydrogenase YfmT | SsS58_07322 (*yfmT*) | 0.0107 | 0.0152 | 0.0232 |
| A0A086GSF6 | Isocitrate dehydrogenase [NADP] | IQ62_25220 | 0.1285 | 0.0970 | 0.1040 |
| C9Z120 | Oxidoreductase | SCAB_56051 | 0.0069 | 0.0106 | 0.0128 |
| C9Z5Y8 | Oxidoreductase | SCAB_74311 | 0.0087 | 0.0384 | 0.0249 |
| A0A1C6PGX6 | L-glyceraldehyde 3-phosphate reductase | B046DRAFT_04129 | 0.0541 | 0.0343 | 0.0285 |
| A0A086H0J5 | L-glyceraldehyde 3-phosphate reductase | IQ62_09670 | 0.3397 | 0.1757 | 0.1319 |
| A0A1C4KFD2 | Menaquinol-cytochrome c reductase iron-sulfur subunit | GA0115242_109650 | 0.0591 | 0.0224 | 0.0443 |
| C9Z3L5 | Aldehyde dehydrogenase | SCAB_25771 | ND | 0.0120 | 0.0217 |
| A0A101S300 | Aconitate hydratase | AQJ54_17655 (*acnA*) | 0.1168 | 0.1011 | 0.0960 |
| A0A1C5EI39 | Aldehyde dehydrogenase | GA0115260_104793 | 0.1357 | 0.2159 | 0.1700 |
| A0A081XPK3 | ATP synthase subunit alpha | BU52_19755 (*atpA*) | 0.1625 | 0.1309 | 0.1103 |
| A0A086GWS5 | Succinate dehydrogenase | IQ62_16970 (*sdhA*) | 0.0064 | 0.0086 | 0.0361 |
| D9XG05 | ATP synthase subunit alpha | SSQG_05404 (*atpA*) | 0.1977 | 0.1635 | 0.1545 |
| A0A124IA70 | ATP synthase subunit alpha | AQJ67_09680 (*atpA*) | 0.1910 | 0.1956 | 0.1039 |
| A0A100JPX1 | Pyruvate, phosphate dikinase | SsS58_03888 (*ppdK_3*) | ND | 0.0036 | 0.0280 |
| C9ZGA4 | NT-sugar reductase | SCAB_3361 | 0.0738 | 0.0690 | 0.0782 |
| A0A0M8TGK4 | ATP synthase subunit beta | ADK57_32585 (*atpD*) | 0.2558 | 0.3314 | 0.2712 |
| C9YXC1 | Alcohol dehydrogenase class III | SCAB_7101 (*adhC* ) | 0.0371 | 0.1058 | 0.1313 |
| A0A117RSH8 | ATP synthase subunit alpha | AQJ58_39055 (*atpA*) | 0.1124 | 0.1500 | 0.1665 |
| A0A177HWN5 | ATP synthase subunit beta | STSP_13270 (*atpD*) | 0.1089 | 0.3086 | 0.0982 |
| I1Z0X2 | ATP synthase subunit beta (Fragment) | *atpD* | 0.2125 | 0.0913 | 0.0791 |
| A0A0X3W4F0 | Isocitrate dehydrogenase [NADP] | ADL30_25195 | 0.0646 | 0.0200 | 0.0318 |
| D7CHE7 | Alpha-ketoglutarate decarboxylase | SBI_03883 (*kgd*) | 0.0262 | 0.0212 | 0.0223 |
| A0A0L8QHA4 | Alpha-ketoglutarate decarboxylase (Fragment) | ADK38_30065 (*kgd*) | 0.0391 | 0.0348 | 0.0333 |
| A0A1K1VQG1 | 2-oxoglutarate dehydrogenase E1 component | SAMN02787144_100266 | 0.0258 | 0.0291 | 0.0249 |
| A0A101U4B6 | Ubiquinol-cytochrome C reductase | AQJ67_13545 | 0.0511 | 0.0259 | 0.0220 |
| A0A1V2RFL9 | ATP synthase subunit alpha | STBA_19010 (*atpA*) | 0.1438 | 0.1063 | 0.1124 |
| L1KK73 | Pyruvate dehydrogenase E1 component | STRIP9103_02727 (*aceE_2*) | 0.0886 | 0.0317 | 0.0369 |
| A0A101JBT2 | Aconitate hydratase | ADL12_38700 (*acnA*) | 0.0420 | 0.0943 | 0.0640 |
| A0A0X3X1R3 | Aldehyde dehydrogenase | ADL30_05350 | 0.0626 | 0.0372 | 0.1461 |
| A0A1A9BM54 | NADH-quinone oxidoreductase | GA0115233_1003146 | 0.0245 | 0.0147 | 0.0152 |
| A0A1J4NVA0 | 2-oxoglutarate dehydrogenase, E2 component, dihydrolipoamide succinyltransferase (Fragment) | WN71_023115 | 0.1631 | 0.1193 | ND |
| E2PVD8 | Isocitrate dehydrogenase [NADP] | SCLAV_0808 (*icdA*) | 0.0419 | 0.0127 | 0.0244 |
| A0A1C4S0U3 | ATP synthase subunit alpha | GA0115242_135910 (*atpA*) | 0.0662 | 0.0731 | 0.0925 |
| A0A022MJK8 | Pyruvate dehydrogenase E1 component | CF54_22890 | 0.0366 | 0.0204 | 0.0123 |
| A0A0L0KSG9 | Dihydrolipoyl dehydrogenase | IQ64_30375 | 0.0880 | 0.3747 | 0.1613 |
| A0A0C5GBX8 | Dihydrolipoyl dehydrogenase | TU94_09485 | ND | 0.1832 | 0.0703 |
| A0A124IEJ1 | NAD-glutamate dehydrogenase | AQJ91_24905 | 0.0059 | ND | 0.0097 |
| C9ZHB0 | F420-dependent NADP reductase | SCAB_66321 | 0.0521 | 0.0537 | 0.0511 |
| C9YWW5 | Electron transfer flavoprotein, beta subunit | SCAB_82671 | 0.0256 | 0.0373 | 0.0124 |
| C9YXV9 | NADH-quinone oxidoreductase subunit D | SCAB_38061 (*nuoD*) | 0.1590 | 0.1297 | 0.1551 |
| C9YXW1 | NADH-quinone oxidoreductase subunit B | SCAB_38081 (*nuoB*) | 0.1413 | 0.1517 | 0.1687 |
| C9YXV1 | NADH dehydrogenase subunit NuoL | SCAB_37981 (*nuoL*) | 0.0340 | 0.0209 | 0.0367 |
| C9YXW0 | NADH-quinone oxidoreductase subunit C | SCAB_38071 (*nuoC*) | 0.0782 | 0.0635 | 0.0372 |
| A0A0M4E3V9 | ATP synthase subunit alpha | ABE83_10710 (*atpA*) | 0.0899 | 0.0530 | 0.1206 |
| C9Z1V2 | Oxidoreductase | SCAB_9451 | ND | 0.0070 | 0.0295 |
| Inorganic ion metabolism | | | | | |
| C9YUJ7 | Nitrite/sulphite reductase | SCAB_19771 | 0.0097 | 0.0251 | 0.0252 |
| C9ZCX9 | Uncharacterized protein | SCAB_1391 | ND | 0.0274 | 0.0358 |
| C9ZCY1 | AMP-binding NRPS ligase | SCAB_1411 | 0.0047 | 0.0214 | 0.0176 |
| C9ZBH8 | Oxidoreductase alpha subunit | SCAB_15301 | 0.0675 | 0.0725 | 0.0836 |
| C9YVN0 | Ferredoxin reductase family protein | SCAB_5821 | 0.0514 | 0.0235 | 0.0409 |
| C9YXB6 | [2Fe-2S] protein | SCAB_7051 | 0.0338 | 0.0151 | 0.0401 |
| C9YTX8 | Bacterioferritin | SCAB_67681 (*bfr*) | 0.5720 | 0.3863 | 0.5865 |
| C9ZH25 | Phosphate-specific transport system accessory protein PhoU | SCAB_50031 | 0.2040 | 0.2158 | 0.2363 |
| C9ZFJ5 | Phosphate-binding protein PstS | SCAB_49311 (*pstS*) | 0.1777 | 0.0920 | 0.1391 |
| C9YZX2 | Iron complex transport system ATP-binding protein | SCAB_71511 | 0.1504 | 0.1129 | 0.0753 |
| C9YZU6 | Sodium:solute symporter | SCAB_71241 | 0.0413 | 0.0482 | 0.0657 |
| C9Z5D4 | Secreted oligopeptide-binding transport system protein | SCAB_27411 | 0.4305 | 0.3798 | 0.2834 |
| C9YUK3 | Lipoprotein (NitT/TauT family transporter) | SCAB_19841 | 0.0550 | 0.0166 | 0.0322 |
| C9Z5P9 | Iron(III) transporter | SCAB_58601 | ND | ND | 0.0692 |
| Lipid metabolism | | | | | |
| C9ZED6 | coA ligase | SCAB_79671 | 0.4130 | 0.3144 | 0.3351 |
| C9Z2I3 | Acetyl-coenzyme A synthetase | SCAB_41241 (*acsA*) | 0.1575 | 0.1939 | 0.2474 |
| C9YTK3 | Secreted peptidase | SCAB_51091 | 0.1432 | 0.2022 | 0.1350 |
| C9Z1E2 | Acyl-CoA dehydrogenase | SCAB_72171 | 0.1610 | 0.1489 | 0.1690 |
| C9Z6U1 | Enoyl-coA hydratase/isomerase family protein | SCAB_27801 | 0.2108 | 0.2332 | 0.2550 |
| C9YUX3 | Glycerol-3-phosphate dehydrogenase | SCAB_36181 | 0.1059 | 0.0679 | 0.0987 |
| C9ZGW2 | Acyl-CoA carboxylase alpha subunit | SCAB_34621 (*accA2*) | 0.0849 | 0.0632 | 0.0738 |
| A0A100JM29 | Acetyl-CoA acetyltransferase | SsS58_02397 (*thlA*) | 0.1131 | 0.1079 | 0.1105 |
| C9ZBL2 | PROBABLE ESTERASE/LIPASE LIPP | SCAB_15651 (*lipP*) | 0.0992 | 0.0894 | 0.0898 |
| C9Z9F5 | 3-oxoacyl-[acyl-carrier protein] reductase | SCAB_76541 | 0.1053 | 0.1319 | 0.1606 |
| C9Z264 | Acyl CoA dehydrogenase | SCAB_25501 | 0.0293 | 0.0456 | 0.1016 |
| C9YYH5 | Dihydroxyacetone kinase component | SCAB_83811 (*dhaK*) | 0.1029 | 0.0925 | 0.0662 |
| M3EDJ2 | FadA protein | SBD_3919 | 0.0482 | 0.0912 | 0.0626 |
| C9ZBW2 | Acyl CoA isomerase | SCAB_31181 (*chcB*) | 0.1533 | 0.1114 | 0.1015 |
| C9ZGV4 | Enoyl-CoA hydratase | SCAB_34521 | 0.0966 | 0.0918 | 0.1358 |
| C9ZFX1 | Dehydrogenase/reductase | SCAB_79721 | 0.1093 | 0.0775 | 0.1389 |
| C9ZC71 | Fatty acid CoA ligase | SCAB_47091 | 0.0461 | 0.0344 | 0.0341 |
| C9ZGV7 | Propionyl-CoA carboxylase complex B subunit | SCAB_34571 (*pccB*) | 0.0361 | 0.0345 | 0.0422 |
| C9ZFX0 | Oxidoreductase | SCAB_79711 | 0.0192 | 0.0451 | 0.0284 |
| C9Z980 | Long-chain fatty-acid CoA ligase | SCAB_61471 | 0.0405 | 0.0379 | 0.0334 |
| C9YX84 | Fused isobutyryl-CoA mutase | SCAB_6731 (*icmF*) | 0.0104 | 0.0155 | 0.0216 |
| C9Z8D6 | 3-hydroxybutyryl-coA dehydrogenase | SCAB_28641 | 0.0811 | 0.0859 | 0.0712 |
| C9YU82 | Phosphodiesterase | SCAB_82571 | 0.0980 | 0.0782 | 0.0907 |
| C9YV55 | Uncharacterized protein | SCAB_52141 | 0.0296 | 0.0284 | 0.0427 |
| C9YZV5 | Enoyl-[acyl-carrier-protein] reductase [NADH] | SCAB_71331 | 0.0326 | 0.0639 | 0.0447 |
| C9Z5Z2 | Secreted glycerophosphoryl diester phosphodiesterase | SCAB_74351 (*glpQ3*) | 0.0188 | 0.0318 | 0.0406 |
| C9ZCM0 | Acyltransferase | SCAB_78441 | 0.0514 | 0.0563 | 0.1115 |
| C9Z3U2 | Glycerol-3-phosphate dehydrogenase [NAD(P)+] | SCAB_26541 (*gpdA gpsA*) | 0.0421 | 0.0466 | 0.0215 |
| A0A0L8NH38 | 3-oxoacyl-ACP reductase | ADK77_30640 | 0.0629 | 0.0345 | 0.0368 |
| C9ZCG0 | Malonyl-CoA/methylmalonyl-CoA synthetase | SCAB_63511 | 0.0247 | 0.0286 | 0.0113 |
| C9Z8C1 | VOC domain-containing protein | SCAB_28491 | 0.0769 | 0.0404 | 0.0806 |
| C9Z9B9 | Acetoacetyl-CoA synthetase | SCAB_76181 (*acsA*) | 0.0225 | 0.0128 | 0.0121 |
| C9ZDZ0 | Malonyl CoA:acyl carrier protein malonyltransferase | SCAB_64161 (*fabD*) | 0.0310 | 0.0390 | 0.0254 |
| C9YTW9 | Lipase | SCAB_67581 | 0.0235 | 0.0344 | 0.0335 |
| C9Z865 | Fatty acid oxidative multifunctional enzyme | SCAB_13351 (*fadB1*) | 0.0052 | 0.0101 | 0.0144 |
| C9Z479 | Acyl-CoA dehydrogenase | SCAB_58051 (*fadE7*) | 0.0105 | 0.0334 | 0.0188 |
| C9ZGL8 | Long-chain-fatty-acid-CoA ligase | SCAB_18621 | 0.0390 | 0.0025 | 0.0072 |
| C9YVX2 | Fatty acid oxidation complex alpha-subunit | SCAB_20961 | 0.0059 | 0.0085 | 0.0150 |
| A0A0Q9AC72 | Glycerol kinase | ASE41_24365 (*glpK*) | 0.1291 | 0.1381 | 0.1344 |
| V6KKE6 | Biotin carboxyl carrier protein | M878_15880 | 0.0064 | 0.0148 | 0.0120 |
| A0A1U9QSP3 | Acetyl-coenzyme A synthetase | BBN63_14590 (*acsA*) | 0.0209 | 0.0273 | 0.0839 |
| C9ZDM8 | Dehydrogenase | SCAB_47481 | 0.0491 | 0.0476 | 0.0310 |
| C9ZGZ1 | UPF0678 fatty acid-binding protein-like protein SCAB_49671 | SCAB_49671 | 0.1078 | 0.0611 | 0.0598 |
| Nucleotide metabolism | | | | | |
| C9YUX6 | Inosine-5'-monophosphate dehydrogenase | SCAB_36211 (*guaB*) | 0.0701 | 0.1066 | 0.1200 |
| A0A100JYR8 | Adenylosuccinate synthetase | SsS58_08606 (*purA*) | 0.1304 | 0.0855 | 0.1027 |
| C9ZB78 | Adenylosuccinate lyase | SCAB_78051 | 0.1007 | 0.0502 | 0.0640 |
| C9YUT1 | Bifunctional purine biosynthesis protein PurH | SCAB_35741 (*purH*) | 0.0586 | 0.0481 | 0.0456 |
| C9ZDL4 | Uracil phosphoribosyltransferase | SCAB_47351 (*upp*) | 0.1389 | 0.1402 | 0.1381 |
| C9YTT8 | Kinase | SCAB_67271 | 0.0980 | 0.0921 | 0.0542 |
| C9ZGF9 | D-hydantoinase | SCAB_17991 | 0.0347 | 0.0348 | 0.0611 |
| C9Z7J8 | Bifunctional protein PyrR | SCAB_75181 (*pyrR*) | 0.1490 | 0.0992 | 0.0663 |
| C9YUW0 | GMP synthase [glutamine-hydrolyzing] | SCAB_36051 (*guaA*) | 0.0317 | 0.0374 | 0.0338 |
| C9Z1A7 | CTP synthase | SCAB_71801 (*pyrG*) | 0.0384 | 0.0269 | 0.0294 |
| C9Z7K0 | Dihydroorotase | SCAB_75201 (*pyrC*) | 0.0461 | 0.0367 | 0.0398 |
| C9YW57 | Adenylate kinase | SCAB_36731 (*adk*) | 0.0520 | 0.0774 | 0.0851 |
| C9ZDQ0 | Phosphoribosylamine--glycine ligase | SCAB_47701 (*purD*) | 0.0539 | 0.0440 | 0.0441 |
| C9YU38 | Oxidoreductase | SCAB_82111 | 0.0133 | 0.0265 | 0.0244 |
| C9Z3N8 | Uridylate kinase | SCAB_26001 (*pyrH*) | 0.0426 | 0.0661 | 0.0677 |
| C9ZDR6 | Phosphoribosylformylglycinamidine cyclo-ligase | SCAB_47861 (*purM*) | 0.0539 | 0.0385 | 0.0339 |
| A0A100JX10 | Phosphoribosylformylglycinamidine synthase subunit PurL | SsS58_07693 (*purL*) | 0.0190 | 0.0207 | 0.0136 |
| C9Z7F8 | Nucleoside diphosphate kinase | SCAB_60011 (*ndk*) | 0.1305 | 0.1207 | 0.1313 |
| C9ZDQ3 | Phosphoribosylaminoimidazole-succinocarboxamide synthase | SCAB_47731 (*purC*) | 0.0583 | 0.0240 | 0.0507 |
| C9ZGK9 | Uricase | SCAB_18521 | 0.0576 | 0.0310 | 0.0643 |
| C9YWT5 | Pseudouridine-5'-phosphate glycosidase | SCAB_69391 (*psuG*) | 0.0132 | 0.0682 | 0.0619 |
| C9ZGX4 | Secreted 5'-nucleotidase | SCAB_49491 | 0.0150 | 0.0352 | 0.0271 |
| C9ZGG0 | CN hydrolase domain-containing protein | SCAB_18001 | 0.0449 | 0.0581 | 0.0567 |
| C9Z0P9 | Guanosine-5'-triphosphate.3'-diphosphate pyrophosphatase | SCAB_39321 | 0.0583 | 0.0240 | 0.0340 |
| C9YWF6 | Purine nucleoside phosphorylase | SCAB_53071 | 0.0390 | 0.0416 | 0.0552 |
| C9YT86 | Thymidine phosphorylase | SCAB_34921 (*deoA*) | 0.0285 | 0.0189 | 0.0295 |
| A0A100JUT9 | 5'-nucleotidase | SsS58_06520 | 0.0692 | 0.0697 | 0.0489 |
| C9Z7K4 | Dihydroorotate dehydrogenase (quinone) | SCAB_75241 (*pyrD*) | 0.0289 | 0.0123 | 0.0410 |
| C9Z995 | Hit-family protein | SCAB_61621 | 0.0704 | 0.0779 | 0.0558 |
| C9ZGG3 | Hydrolase | SCAB_18031 | 0.0126 | 0.0326 | 0.0366 |
| A0A1B1B401 | Adenylosuccinate lyase | AVL59_31870 | 0.0461 | 0.0290 | 0.0445 |
| A0A0M9ZDX9 | Uridylate kinase | ADL00_43000 (*pyrH*) | ND | 0.0453 | ND |
| C9Z0M0 | Vitamin B12-dependent ribonucleotide reductase | SCAB_24631 (*nrdJ*) | 0.0217 | 0.0236 | 0.0185 |
| Post-translational modification, protein turnover and proteolysis | | | | | |
| A0A117EE94 | Peptidase_M48 domain-containing protein | SsS58_03792 | 0.1457 | 0.1934 | 0.2228 |
| C9Z0I8 | M16 family peptidase | SCAB_24271 | 0.1262 | 0.1113 | 0.0894 |
| C9YZX4 | Secreted protein (Modulator of FtsH protease HflK) | SCAB_71541 | 0.1990 | 0.1838 | 0.1814 |
| C9Z0I7 | M16 family peptidase | SCAB_24261 | 0.1048 | 0.0861 | 0.0819 |
| C9Z4D8 | WYL domain-containing protein | SCAB_73551 | 0.0708 | 0.0629 | 0.0634 |
| C9Z4D4 | Pup--protein ligase | SCAB_73511 (*pafA*) | 0.3449 | 0.3088 | 0.3659 |
| C9Z4D0 | Proteasome subunit beta | SCAB_73471 (*prcB*) | 0.3647 | 0.3905 | 0.3857 |
| A0A100JRU0 | AAA ATPase forming ring-shaped complexes | SsS58_04932 (*arc*) | 0.1112 | 0.1046 | 0.0930 |
| C9Z4D1 | Proteasome subunit alpha | SCAB_73481 (*prcA*) | 0.2573 | 0.2336 | 0.2581 |
| C9ZAJ2 | Peptidyl-prolyl cis-trans isomerase | SCAB_45651 | 0.2500 | 0.2721 | 0.2756 |
| C9Z7F0 | ATP-dependent Clp protease proteolytic subunit | SCAB_59941 (*clpP clpP1*) | 0.2383 | 0.2110 | 0.1552 |
| C9Z0U4 | ATP-dependent zinc metalloprotease FtsH | SCAB_39781 (*ftsH*) | 0.0445 | 0.0523 | 0.0649 |
| C9ZBS9 | Sulfurylase | SCAB_30851 | 0.0954 | 0.0560 | 0.0683 |
| C9Z4C8 | Proteasome component | SCAB_73451 | 0.0551 | 0.0460 | 0.0503 |
| C9Z4D5 | Peptidylprolyl isomerase | SCAB_73521 (*fkbB*) | 0.1128 | 0.0995 | 0.0975 |
| C9Z6Y7 | Thioredoxin | SCAB_28281 | 0.0917 | 0.0843 | 0.0944 |
| C9Z7F1 | ATP-dependent Clp protease proteolytic subunit | SCAB_59951 (*clpP clpP2*) | 0.1346 | 0.1166 | 0.0907 |
| A0A124C5I2 | Peptide methionine sulfoxide reductase MsrA | SsS58_08114 (*msrA3 msrA*) | 0.1031 | 0.0921 | 0.1150 |
| C9Z4C9 | Prokaryotic ubiquitin-like protein Pup (Bacterial ubiquitin-like modifier) | SCAB_73461 (*pup*) | 0.1751 | 0.1663 | 0.1701 |
| C9ZHK2 | ATP-dependent zinc metalloprotease FtsH | SCAB_80861 | 0.0124 | 0.0162 | 0.0239 |
| C9Z7I2 | Peptidase | SCAB_60251 | 0.1114 | 0.1964 | 0.2409 |
| A0A100JL60 | Protease TldD | SsS58_01892 | 0.1142 | 0.1107 | 0.1053 |
| C9ZC88 | NAD(P)/FAD-dependent oxidoreductase | SCAB_62711 | 0.0536 | ND | 0.0048 |
| A0A0W7WTT0 | Proteasome subunit beta | AT728_01885 (*prcB*) | 0.1202 | 0.1419 | 0.1364 |
| A0A385DHU0 | Pup--protein ligase | D0C37_27930 pafA | ND | 0.1530 | 0.1715 |
| Replication, recombination and DNA repair | | | | | |
| C9Z650 | Uncharacterized protein | SCAB_74971 | 0.1495 | 0.1301 | 0.1834 |
| C9Z210 | Protein RecA (Recombinase A) | SCAB_24951 (*recA*) | 0.1836 | 0.0899 | 0.0806 |
| C9YWP5 | DNA polymerase I | SCAB_68981 (*polA*) | 0.0464 | 0.0395 | 0.0367 |
| C9Z108 | DNA helicase | SCAB_55921 | 0.0331 | 0.0145 | 0.0223 |
| C9Z0M2 | LexA repressor | SCAB_24651 (*lexA*) | 0.0561 | 0.0955 | 0.0478 |
| C9ZGH3 | Exonuclease | SCAB_18151 | 0.1145 | 0.0458 | 0.0533 |
| C9Z5B8 | DNA ligase | SCAB_27251 (*ligA*) | 0.0258 | 0.0142 | 0.0165 |
| C9Z0I9 | DNA topoisomerase | SCAB_24291 | 0.0091 | 0.0097 | 0.0223 |
| A0A117EGM7 | DNA polymerase I | SsS58_07822 (*polA_2 polA*) | 0.0303 | 0.0249 | 0.0239 |
| A0A1B1M7W1 | DNA polymerase I | SLCG_6094 SLINC_2319 (*polA*) | ND | 0.0094 | ND |
| C9ZAK1 | DNA gyrase subunit A | SCAB_45751 (*gyrA*) | 0.1392 | 0.1179 | 0.1290 |
| C9Z2D8 | DNA topoisomerase 1 | SCAB_40781 (*topA*) | 0.0685 | 0.0598 | 0.0824 |
| C9ZAK2 | DNA gyrase subunit B | SCAB_45761 (*gyrB*) | 0.0783 | 0.0559 | 0.0737 |
| C9YW00 | RNA helicase | SCAB_21251 | 0.0072 | 0.0136 | 0.0180 |
| C9ZAK6 | Beta sliding clamp | SCAB_45811 (*dnaN*) | 0.0705 | 0.1229 | 0.0830 |
| C9ZAN4 | Single-stranded DNA-binding protein (SSB) | SCAB_46111 (*ssb2*) | 0.1620 | 0.1704 | 0.1444 |
| C9YVZ8 | Helicase SNF2 family protein | SCAB_21231 | 0.0049 | 0.0157 | 0.0129 |
| C9YUU7 | ATP-dependent DNA helicase | SCAB_35921 | 0.0106 | 0.0078 | 0.0131 |
| A0A0M8UW85 | DNA gyrase subunit B | ADL00_37655 (*gyrB*) | 0.0280 | 0.0149 | 0.0152 |
| Transcription and RNA processing | | | | | |
| C9YWW2 | Nucleotide-binding protein SCAB_69661 | SCAB_69661 | 0.0251 | 0.0363 | 0.0435 |
| A0A100JSY7 | Polyribonucleotide nucleotidyltransferase | SsS58_05524 (*pnp*) | 0.3063 | 0.2996 | 0.3239 |
| C9Z233 | Ribonuclease J | SCAB_25181 (*rnj*) | 0.1316 | 0.0930 | 0.0994 |
| C9Z7H1 | S1 motif domain-containing protein | SCAB_60141 | 0.0144 | 0.0332 | 0.0335 |
| C9Z4S3 | Ribonuclease J | SCAB_87641 (*rnj*) | 0.0692 | 0.0475 | 0.0603 |
| C9Z3S7 | Ribonuclease 3 | SCAB_26391 (*rnc*) | 0.0443 | 0.0562 | 0.0330 |
| C9Z2E1 | Ribonucleotide methyltransferase | SCAB_40811 | 0.0175 | 0.0222 | 0.0186 |
| C9ZAM4 | RNA nucleotidyltransferase | SCAB_45991 | 0.0206 | 0.0158 | 0.0230 |
| A0A117EET8 | tRNA (adenine(58)-N(1))-methyltransferase TrmI | SsS58_04935 (*trmI*) | 0.0179 | 0.0261 | 0.0315 |
| C9Z2I8 | Ribonuclease PH | SCAB_56731 (*rph*) | 0.0221 | 0.0332 | 0.0257 |
| F8JUP6 | Polyribonucleotide nucleotidyltransferase | SCATT_45040 (*pnp*) | 0.0884 | 0.0814 | 0.0782 |
| A0A100JSZ1 | Ribonuclease J | SsS58_05517 (*rnj*) | 0.0663 | 0.0577 | 0.0617 |
| A0A0M9ZEE2 | Ribonuclease J | ADL00_40440 (*rnj*) | ND | 0.0471 | 0.0274 |
| S3BBE9 | HTH cro/C1-type domain-containing protein | HMPREF1486_00810 | 0.1454 | 0.1349 | 0.1026 |
| C9YTS9 | Phage shock protein A (PspA) | SCAB_67181 | 0.0212 | 0.0968 | 0.0614 |
| C9Z4D7 | WYL domain-containing protein | SCAB_73541 | 0.0347 | 0.0280 | 0.0209 |
| C9YWA1 | DNA-directed RNA polymerase subunit beta' | SCAB_37181 (*rpoC*) | 0.2962 | 0.2600 | 0.2688 |
| C9YWA2 | DNA-directed RNA polymerase subunit beta | SCAB_37191 (*rpoB*) | 0.2448 | 0.2148 | 0.1873 |
| C9YW51 | DNA-directed RNA polymerase subunit alpha | SCAB_36671 (*rpoA*) | 0.5318 | 0.4956 | 0.5101 |
| C9Z8G5 | Transcription termination factor Rho | SCAB_28931 (*rho*) | 0.1343 | 0.2208 | 0.1584 |
| A0A124C4Y8 | HTH-type transcriptional regulator DegA | SsS58_06386 (*degA_4*) | 0.1439 | 0.1239 | 0.1047 |
| C9YV68 | Chitinase-promoter-binding protein | SCAB_52291 | 0.1793 | 0.1799 | 0.1464 |
| C9YWA8 | Transcription termination/antitermination protein NusG | SCAB_37251 (*nusG*) | 0.1691 | 0.1323 | 0.1130 |
| C9ZAC1 | Sigma factor | SCAB_30101 (*sig1*) | 0.0789 | 0.1048 | 0.1029 |
| C9Z459 | A-factor-responsive transcriptional activator | SCAB_57831 (*adpA*) | 0.0857 | 0.0880 | 0.1025 |
| C9YUZ0 | Transcription regulation protein | SCAB_36351 | 0.1389 | 0.2295 | 0.2426 |
| C9Z252 | Transcription termination/antitermination protein NusA | SCAB_25371 (*nusA*) | 0.0966 | 0.0663 | 0.0634 |
| C9Z0K3 | RNA polymerase sigma factor SigA | SCAB_24441 (*hrdB sigA*) | 0.0293 | 0.0527 | 0.0465 |
| C9Z2E6 | Anti-sigma factor antagonist | SCAB_40861 | 0.2580 | 0.2179 | 0.2246 |
| C9ZBT7 | TetR-family transcriptional regulator | SCAB_30931 | 0.0699 | 0.1032 | 0.0965 |
| C9Z8P0 | Regulatory protein phosphatase | SCAB_44531 | 0.0156 | 0.0128 | 0.0117 |
| C9YZN7 | IclR-family transcriptional regulator | SCAB_70641 | 0.0651 | 0.0458 | 0.0461 |
| C9Z2W2 | TetR-family transcriptional regulator | SCAB_72821 | 0.1053 | 0.0567 | 0.0911 |
| C9ZGM1 | Transcriptional regulator | SCAB_18651 | 0.0083 | 0.0113 | 0.0139 |
| C9YXX5 | Cold shock protein | SCAB_38231 (*scoF2*) | 0.2077 | 0.2312 | 0.1995 |
| C9YT85 | Sigma factor | SCAB_34911 | 0.0389 | 0.0276 | 0.0425 |
| C9Z7Q6 | AsnC-family transcriptional regulatory protein | SCAB_75801 | 0.0887 | 0.0824 | 0.0999 |
| C9ZFH7 | TetR-family regulator | SCAB_49111 | 0.0471 | 0.0715 | 0.0367 |
| C9YVX9 | Probable transcriptional repressor protein | SCAB_21031 | 0.0300 | 0.0278 | 0.0163 |
| C9Z2Z6 | GntR-family transcriptional regulator | SCAB_73181 | 0.0557 | 0.0405 | 0.0716 |
| A0A117EEY8 | DNA-binding transcriptional regulator EnvR | SsS58_05240 | 0.0260 | 0.0289 | 0.0285 |
| C9ZGW4 | DeoR-family transcriptional regulator | SCAB_34641 | 0.0174 | 0.0579 | 0.0120 |
| C9Z7L7 | Ligand-binding protein | SCAB_75381 | 0.0151 | 0.0253 | 0.0204 |
| V6K9A9 | DNA-directed RNA polymerase subunit beta' | M877_15630 (*rpoC*) | 0.2475 | 0.2025 | 0.2023 |
| C9YWR3 | GntR-family transcriptional regulator | SCAB_69171 | 0.0103 | 0.0334 | 0.0209 |
| D6K124 | Transcription termination factor Rho (Fragment) | SSTG_03398 | 0.0901 | 0.0572 | 0.1098 |
| A0A0M9ZL73 | DNA-directed RNA polymerase subunit beta' | ADK64_24110 (*rpoC*) | 0.1817 | 0.1969 | 0.1059 |
| A0A0U3QLT8 | Transcription termination factor Rho | AS200_16710 (*rho*) | 0.0638 | 0.0408 | 0.0789 |
| A0A1I2IKF5 | DNA-directed RNA polymerase subunit beta' | SAMN02787118_106325 (*rpoC*) | 0.2679 | 0.1026 | 0.1131 |
| A0A1V4A7L7 | DNA-directed RNA polymerase subunit beta | B1H18_17010 (*rpoB*) | 0.1500 | 0.1032 | 0.1341 |
| L7EQC9 | DNA-directed RNA polymerase subunit beta | STRTUCAR8_04485 (*rpoB*) | 0.0821 | 0.0713 | 0.0706 |
| A0A1J7CCD5 | DNA-directed RNA polymerase subunit beta' | BIV57_01535 (*rpoC*) | 0.1320 | 0.0680 | 0.1244 |
| G1AQV2 | RNA polymerase subunit B (Fragment) | *rpoB* | 0.0225 | 0.0699 | 0.0604 |
| A0A014P9S3 | XRE family transcriptional regulator | Z951_12795 | 0.1192 | 0.0359 | 0.1047 |
| G2GAH7 | DNA-directed RNA polymerase subunit beta | SZN_12498 (*rpoB*) | 0.0495 | 0.0915 | ND |
| Q82DQ5 | DNA-directed RNA polymerase subunit beta | SAV_4914 (*rpoB*) | 0.0442 | 0.0831 | 0.0830 |
| G1AQW6 | RNA polymerase subunit B (Fragment) | *rpoB* | 0.1011 | 0.0459 | 0.0465 |
| D9WRB8 | DNA-directed RNA polymerase subunit beta | SSOG_03798 (*rpoB*) | 0.0248 | 0.0657 | ND |
| Translation, ribosomal structure and biogenesis | | | | | |
| C9YW92 | Elongation factor Tu (EF-Tu) | SCAB_37091 (*tuf tuf1*) | 1.0461 | 1.1662 | 0.8639 |
| C9YWQ2 | 30S ribosomal protein S1 | SCAB_69061 (*rpsA*) | 0.4422 | 0.3744 | 0.4172 |
| C9YW93 | Elongation factor G (EF-G) | SCAB_37101 (*fusA*) | 0.2658 | 0.2558 | 0.2149 |
| C9Z3N9 | Elongation factor Ts (EF-Ts) | SCAB_26011 (*tsf*) | 0.4831 | 0.3940 | 0.2855 |
| C9Z3P0 | 30S ribosomal protein S2 | SCAB_26021 (*rpsB*) | 0.3625 | 0.3908 | 0.3752 |
| C9Z8T7 | Aspartate--tRNA ligase | SCAB_44991 (*aspS*) | 0.1426 | 0.1154 | 0.1367 |
| A0A117EGS1 | Lysine--tRNA ligase | SsS58_08083 (*lysS*) | 0.1666 | 0.1203 | 0.1576 |
| C9Z2B7 | Cysteine--tRNA ligase | SCAB_40551 (*cysS*) | 0.1842 | 0.1343 | 0.1516 |
| C9Z7J3 | Leucine--tRNA ligase | SCAB_60361 (*leuS*) | 0.0647 | 0.0600 | 0.0776 |
| C9Z659 | Alanine--tRNA ligase | SCAB_75061 (*alaS*) | 0.0913 | 0.0502 | 0.0477 |
| C9ZAS2 | Glycine--tRNA ligase | SCAB_62001 (*glyS glyQS*) | 0.1666 | 0.1040 | 0.0988 |
| C9YZB3 | Arginine--tRNA ligase | SCAB_38961 (*argS*) | 0.1086 | 0.0811 | 0.0990 |
| C9Z257 | Proline--tRNA ligase | SCAB_25421 (*proS*) | 0.1116 | 0.0902 | 0.0844 |
| C9YW94 | 30S ribosomal protein S7 | SCAB_37111 (*rspG rpsG*) | 0.3628 | 0.4196 | 0.3055 |
| C9Z250 | Translation initiation factor IF-2 | SCAB_25351 (*infB*) | 0.0668 | 0.0467 | 0.0383 |
| A0A086HMG2 | Ribosome-recycling factor | IQ62_19975 (*frr*) | 0.3309 | 0.3021 | 0.3635 |
| C9Z8S2 | Methionine--tRNA ligase | SCAB_44841 (*metG*) | 0.0647 | 0.0689 | 0.0748 |
| C9Z7K6 | Uncharacterized protein | SCAB_75261 | 0.5654 | 0.4928 | 0.6240 |
| A0A100JRU6 | Phenylalanine--tRNA ligase beta subunit | SsS58_04872 (*pheT*) | 0.0467 | 0.0491 | 0.0402 |
| C9YW61 | 30S ribosomal protein S5 | SCAB_36771 (*rpsE*) | 0.2976 | 0.2542 | 0.2101 |
| C9YW72 | 30S ribosomal protein S3 | SCAB_36881 (*rpsC*) | 0.1499 | 0.1719 | 0.1568 |
| C9YW66 | 50S ribosomal protein L5 | SCAB_36821 (*rplE*) | 0.2022 | 0.2346 | 0.1927 |
| C9Z7F5 | Valine--tRNA ligase | SCAB_59981 (*valS*) | 0.0510 | 0.0312 | 0.0315 |
| A0A100JPF0 | ABC transporter ATP-binding protein (Energy-dependent translational throttle protein EttA) | SsS58_03636 | 0.0969 | 0.0609 | 0.0390 |
| C9YW64 | 30S ribosomal protein S8 | SCAB_36801 (*rpsH*) | 0.3732 | 0.2517 | 0.2498 |
| C9YW75 | 50S ribosomal protein L2 | SCAB_36911 (*rplB*) | 0.0811 | 0.1622 | 0.1198 |
| C9Z3Q9 | Proline--tRNA ligase | SCAB_26211 (*proS*) | 0.0767 | 0.0599 | 0.0648 |
| C9Z5B3 | Aspartyl/glutamyl-tRNA(Asn/Gln) amidotransferase subunit B | SCAB_27201 (*gatB*) | 0.0707 | 0.0650 | 0.0562 |
| A0A124C3M5 | Serine--tRNA ligase | SsS58_02115 (*serS*) | 0.0998 | 0.0615 | 0.0619 |
| C9Z4I3 | Phenylalanine--tRNA ligase alpha subunit | SCAB_74011 (*pheS*) | 0.1021 | 0.0643 | 0.0621 |
| C9Z5B5 | Glutamyl-tRNA(Gln) amidotransferase subunit A | SCAB_27221 (*gatA*) | 0.0568 | 0.0618 | 0.0434 |
| C9YW69 | 30S ribosomal protein S17 | SCAB_36851 (*rpsQ*) | 0.2260 | 0.3815 | 0.2855 |
| C9Z656 | 30S ribosomal protein S4 | SCAB_75031 (*rspD rpsD*) | 0.1488 | 0.2189 | 0.1277 |
| C9YWA6 | 50S ribosomal protein L1 | SCAB_37231 (*rlpA rplA*) | 0.1256 | 0.1277 | 0.0818 |
| C9YW46 | 30S ribosomal protein S9 | SCAB_36621 (*rpsI*) | 0.1564 | 0.2437 | 0.1790 |
| C9Z1C5 | GTPase Der (GTP-binding protein EngA) | SCAB_72001 (*engA der*) | 0.0574 | 0.0457 | 0.0523 |
| C9Z629 | Threonine--tRNA ligase | SCAB_74751 (*thrS*) | 0.0395 | 0.0329 | 0.0292 |
| C9Z7L3 | Methionyl-tRNA formyltransferase | SCAB_75341 (*fmt*) | 0.0972 | 0.0606 | 0.0630 |
| C9YW63 | 50S ribosomal protein L6 | SCAB_36791 (*rplF*) | 0.1176 | 0.2101 | 0.0811 |
| C9Z4H8 | Translation initiation factor IF-3 | SCAB_73961 (*infC*) | 0.1254 | 0.1019 | 0.0923 |
| C9Z241 | 30S ribosomal protein S15 | SCAB_25261 (*rpsO*) | 0.2515 | 0.2114 | 0.2290 |
| C9YZV1 | Tyrosine--tRNA ligase | SCAB_71291 (*tyrS*) | 0.0554 | 0.0526 | 0.0379 |
| C9Z3Q3 | 50S ribosomal protein L19 | SCAB_26151 (*rplS*) | 0.2030 | 0.1660 | 0.1653 |
| C9YW53 | 30S ribosomal protein S13 | SCAB_36691 (*rpsM*) | 0.1725 | 0.1661 | 0.0837 |
| C9Z668 | Elongation factor P (EF-P) | SCAB_75151 (*efp*) | 0.1231 | 0.0925 | 0.1057 |
| E5FYQ7 | Translation elongation factor Tu (Fragment) | - | 0.3665 | 0.2430 | 0.3347 |
| A0A132MXX0 | 50S ribosomal protein L14 | LI90_3742 TH66_13215 TR74_20025 (*rplN*) | 0.1692 | 0.1291 | 0.1735 |
| C9Z653 | Histidine--tRNA ligase | SCAB_75001 (*hisS*) | 0.0400 | 0.0354 | 0.0317 |
| C9Z0Y8 | Peptide chain release factor 2 (RF-2) | SCAB_55691 (*prfB*) | 0.0337 | 0.0386 | 0.0327 |
| C9ZAN6 | 50S ribosomal protein L9 | SCAB_46131 (*rplI*) | 0.1419 | 0.0846 | 0.0724 |
| C9YWA4 | 50S ribosomal protein L10 | SCAB_37211 (*rplJ*) | 0.1077 | 0.0921 | 0.0484 |
| A0A124C3F5 | Peptide deformylase | SsS58_01480 (*def_1 def*) | 0.0958 | 0.0469 | 0.0503 |
| C9Z248 | Ribosome-binding factor A | SCAB_25331 (*rbfA*) | 0.1330 | 0.0787 | 0.0776 |
| C9YWA3 | 50S ribosomal protein L7/L12 | SCAB_37201 (*rplL*) | 0.1181 | 0.1236 | 0.0955 |
| A0A081XX37 | 50S ribosomal protein L3 | BU52_06685 (*rplC*) | 0.0925 | 0.0526 | 0.0255 |
| C9Z979 | Elongation factor 4 | SCAB_61461 (*lepA*) | 0.0164 | 0.0159 | 0.0177 |
| C9YW71 | 50S ribosomal protein L16 | SCAB_36871 (*rplP*) | 0.0454 | 0.1004 | 0.1134 |
| C9ZH32 | Cysteine--tRNA ligase | SCAB_50111 (*cysS1 cysS*) | 0.0269 | 0.0205 | 0.0262 |
| C9YW50 | 50S ribosomal protein L17 | SCAB_36661 (*rplQ*) | 0.1092 | 0.0853 | 0.0636 |
| C9YW59 | 50S ribosomal protein L15 | SCAB_36751 (*rplO*) | 0.1004 | 0.1252 | 0.0493 |
| C9YVD5 | Isoleucine--tRNA ligase | SCAB_68091 (*ileS*) | 0.0107 | 0.0092 | 0.0072 |
| C9YWA7 | 50S ribosomal protein L11 | SCAB_37241 (*rplK*) | 0.1227 | 0.0799 | 0.0390 |
| C9YW79 | 30S ribosomal protein S10 | SCAB_36951 (*rpsJ*) | 0.1818 | 0.0699 | 0.0418 |
| C9YW47 | 50S ribosomal protein L13 | SCAB_36631 (*rplM*) | 0.0762 | 0.1162 | 0.0716 |
| A0A0F7CQ52 | 30S ribosomal protein S2 | SXIM_44220 (*rpsB*) | 0.2842 | 0.3054 | 0.3151 |
| C9YY11 | 50S ribosomal protein L25 | SCAB_54151 (*rplY ctc*) | 0.0209 | 0.0576 | 0.0279 |
| E5FYP0 | Translation elongation factor Tu (Fragment) | - | 0.5420 | 0.5365 | 0.3765 |
| E5FYP2 | Translation elongation factor Tu (Fragment) | - | 0.4146 | 0.5219 | 0.5598 |
| A0A0M8T091 | Elongation factor G (EF-G) | ADK56_27910 (*fusA*) | 0.0832 | 0.1216 | 0.0665 |
| G2G6P4 | 30S ribosomal protein S1 | SZN_05759 (*rpsA*) | 0.0471 | 0.0915 | 0.1403 |
| C9ZC20 | Serine--tRNA ligase | SCAB_46561 (*serS*) | 0.0324 | 0.0574 | 0.0610 |
| A0A1D8STJ2 | 30S ribosomal protein S17 | BC342_21270 (*rpsQ*) | 0.1738 | 0.1895 | 0.1673 |
| A0A1Q5C2Q1 | Elongation factor Ts (EF-Ts) | AMK11_31075 (*tsf*) | 0.1829 | 0.0883 | 0.0390 |
| A0A1B6AJU9 | Elongation factor Tu (EF-Tu) | SSP35_05_01600 (*tuf*) | 0.1456 | 0.1669 | 0.0737 |
| Q82DQ1 | Elongation factor G (EF-G) | SAV_4919 (*fusA*) | 0.0938 | 0.1421 | 0.1537 |
| A0A1D7VGP5 | Leucine--tRNA ligase | SL103_06205 (*leuS*) | 0.0067 | 0.0019 | 0.0114 |
| A0A1I2HQJ2 | Elongation factor Tu (EF-Tu) | SAMN02787118_105331 (*tuf*) | 0.0735 | 0.1078 | 0.0628 |
| A0A100JPQ4 | Valine--tRNA ligase | SsS58_03767 (*valS_1 valS*) | 0.0385 | 0.0242 | 0.0265 |
| A0A0C1XJ11 | Elongation factor Tu (EF-Tu) | HY68_07095 (*tuf*) | ND | 0.1307 | 0.3423 |
| Secondary metabolism and differentiation | | | | | |
| C9ZD03 | Uncharacterized protein | SCAB_1641 | ND | 0.0338 | 0.0345 |
| C9ZED0 | polyketide dehydratase component | SCAB_79611 | 0.3941 | 0.5817 | 0.6494 |
| C9ZED1 | polyketide KS component | SCAB_79621 | 0.0451 | 0.1119 | 0.0692 |
| C9Z4F1 | RarA (Conservon) homolog | SCAB_73681 (*rarA3*) | 0.0364 | 0.0528 | 0.0470 |
| C9ZED2 | polyketide synthase component | SCAB_79631 | 0.1417 | 0.1179 | 0.1373 |
| C9Z571 | RarD (Conservon) homolog | SCAB_26771 | 0.1103 | 0.1330 | 0.1177 |
| C9ZFL1 | Mycothiol acetyltransferase | SCAB_49481 (*mshD*) | 0.0512 | 0.0661 | 0.0535 |
| C9ZEV2 | Polyketide cyclase / dehydrase and lipid transport | SCAB_17551 | 0.0443 | 0.1274 | 0.0963 |
| C9YYJ3 | Carbomoyltransferase | SCAB_84021 | 0.0613 | 0.1633 | 0.1775 |
| C9ZCZ1 | Carbamoyltransferase | SCAB_1511 | ND | 0.1013 | 0.0994 |
| C9ZGD0 | Serine/threonine protein kinase | SCAB_3621 | 0.0078 | 0.1126 | 0.1154 |
| C9YYT5 | Amidohydro-rel domain-containing protein | SCAB_8041 | 0.0035 | 0.0367 | 0.0423 |
| C9YYJ6 | O-methyltransferase | SCAB_84051 | 0.0690 | 0.1762 | 0.1670 |
| C9YWG0 | Secreted penicillin acylase | SCAB_53111 | 0.0289 | 0.0185 | 0.0376 |
| C9ZED4 | Polyketide synthase | SCAB_79651 | 0.0067 | 0.0065 | 0.0074 |
| C9ZED8 | Monooxygenase | SCAB_79691 | 0.0369 | 0.0496 | 0.0516 |
| C9Z6A5 | 2-hydroxyhepta-2,4-diene-1,7-dioate isomerase | SCAB_88391 | 0.0669 | 0.0410 | 0.0693 |
| C9Z680 | METHYLTRANSFERASE | SCAB_88131 | 0.0467 | 0.0391 | 0.0326 |
| C9ZGF2 | FAD dependent oxidoreductase | SCAB_17921 | 0.0341 | 0.0139 | 0.0255 |
| C9Z4F5 | RarE (Conservon) homolog | SCAB_73721 (*rarE3*) | 0.0078 | 0.0211 | 0.0115 |
| A0A0L0L3E1 | Carbamoyltransferase | IQ64_11950 | 0.0104 | 0.0335 | 0.0183 |
| C9ZD95 | BldKD oligopeptide ABC transporter subunit | SCAB_31511 (*bldKD*) | 0.1068 | 0.0839 | 0.0478 |
| C9ZD97 | BldKB-like transport system extracellular solute-binding protein | SCAB_31531 (*bldKB*) | 0.1480 | 0.3338 | 0.2431 |
| C9Z1F7 | Dienelactone hydrolase | SCAB_72331 | 0.1214 | 0.0888 | 0.1163 |
| C9ZAI3 | Penicillin-binding kinase | SCAB_45561 | 0.0164 | 0.0137 | 0.0282 |
| C9YT75 | Two component system response regulator | SCAB_34801 (*afsQ1*) | 0.1430 | 0.0853 | 0.0569 |
| Secretion and vesicular transport | | | | | |
| C9Z4E2 | Sec-independent protein translocase protein TatA | SCAB_73591 (*tatA*) | 0.1706 | 0.1702 | 0.1982 |
| C9YZK9 | Protein translocase subunit SecA | SCAB_55371 (*secA*) | 0.0417 | 0.0371 | 0.0295 |
| C9Z3R1 | Signal recognition particle protein (Fifty-four homolog) | SCAB_26231 (*ffh*) | 0.0532 | 0.0462 | 0.0471 |
| C9Z644 | Secreted protein | SCAB_74901 | 0.1096 | 0.1603 | 0.1419 |
| Stress and defense mechanism | | | | | |
| A0A0L0JS12 | PAS/PAC sensor protein | IQ64_44240 | 0.0091 | 0.0109 | 0.0159 |
| C9YZK1 | Two-component system response regulator | SCAB_55281 | 0.1825 | 0.1103 | 0.1214 |
| C9YVQ3 | Oxygen regulatory protein NreC | SCAB_6061 | 0.0216 | 0.0441 | 0.1107 |
| C9YVZ9 | Uncharacterized protein (N6_Mtase) | SCAB_21241 | 0.0113 | 0.0094 | 0.0097 |
| C9ZH64 | GroEL protein | SCAB_50441 (*groL groEL groEL2*) | 0.5223 | 0.7277 | 0.3968 |
| C9Z0R6 | Clp-family ATP-binding protease | SCAB_39491 | 0.2222 | 0.2036 | 0.2056 |
| C9YUY4 | GroEL protein | SCAB_36291 (*groL groEL groL1*) | 0.3202 | 0.2839 | 0.2230 |
| C9Z5G9 | Chaperone protein DnaK | SCAB_42541 (*dnaK*) | 0.1958 | 0.1920 | 0.1676 |
| C9Z5E5 | Chaperone protein ClpB | SCAB_42291 (*clpB*) | 0.0820 | 0.1040 | 0.1176 |
| C9YUY5 | GroES protein | SCAB_36301 (*groS groES*) | 0.6017 | 0.4196 | 0.3647 |
| D5ZUP9 | GroEL protein | SSFG_03971 (*groL groEL*) | 0.2788 | 0.4333 | 0.2122 |
| C9ZEQ8 | Chaperone protein DnaK | SCAB_17091 (*dnaK*) | 0.0309 | 0.0610 | 0.0454 |
| C9YY94 | Uncharacterized protein | SCAB_70031 | 0.0188 | 0.0339 | 0.0379 |
| C9YY95 | Fe-S cluster assembly protein SufD | SCAB_70041 | 0.0201 | 0.0359 | 0.0359 |
| C9YY97 | ABC transporter ATP-binding subunit (SufC) | SCAB_70061 | 0.0580 | 0.0933 | 0.0815 |
| C9ZBN1 | Beta-lactamase domain-containing protein | SCAB_15841 | 0.0355 | 0.0150 | 0.0383 |
| A0A1Q5HJ59 | Chaperone protein DnaK | AMK22_00325 (*dnaK*) | 0.0557 | 0.0412 | 0.0390 |
| A0A117EDI7 | GroEL protein | SsS58_02940 (*groL2 groEL groL*) | 0.2167 | 0.6364 | 0.1996 |
| A0A0M9ZIT9 | GroEL protein | ADL01_37585 (*groEL groL*) | 0.1980 | 0.4295 | 0.2431 |
| A0A0L0KGZ9 | GroEL protein | a10_07945 IQ63_11515 Saa2_07774 (*groEL groL groL2*) | 0.2511 | 0.5387 | 0.1864 |
| A0A0B5DCE1 | GroEL protein | SNOD_15365 (*groEL groL*) | 0.1445 | 0.2055 | 0.1527 |
| A0A0C1X3L7 | GroEL protein | HY68_13130 (*groEL groL*) | 0.1926 | 0.4028 | ND |
| A0A0M2GLQ4 | GroEL protein | UK15_13730 (*groEL groL*) | 0.1659 | 0.2245 | 0.0690 |
| A0A0T1UNS4 | GroEL protein | ASD48_08975 (*groEL groL*) | 0.1445 | 0.0845 | 0.1342 |
| C9YX04 | TerD domain-containing protein | SCAB_83061 | 0.0242 | 0.0417 | 0.0299 |
| C9Z1W0 | Catalase-peroxidase | SCAB_9531 (*cpeB katG*) | 0.2817 | 0.4756 | 0.5110 |
| C9ZE07 | Stress protein | SCAB_64331 | 0.5582 | 0.8763 | 0.9755 |
| C9ZH47 | Stress protein | SCAB_50261 | 0.3568 | 0.5547 | 0.5790 |
| C9Z7C8 | Superoxide dismutase | SCAB_59731 (*sodF2*) | 0.2260 | 0.2589 | 0.3294 |
| C9ZAL8 | Thioredoxin reductase | SCAB_45931 (*trxB*) | 0.1231 | 0.1216 | 0.1657 |
| C9YWU0 | Stress-induced protein | SCAB_69441 | 0.2826 | 0.2427 | 0.2085 |
| C9ZE08 | Stress protein | SCAB_64341 | 0.1495 | 0.1631 | 0.2881 |
| C9ZEQ9 | ATP/GTP binding protein | SCAB_17101 | 0.1956 | 0.2038 | 0.2319 |
| C9YZM9 | Stress inducible protein | SCAB_55571 | 0.0436 | 0.1635 | 0.1784 |
| C9ZHS9 | Tellurium resistance protein | SCAB_81661 (*terD*) | 0.1454 | 0.3592 | 0.2475 |
| C9ZBQ9 | Stress protein | SCAB_30631 | 0.0455 | 0.0287 | 0.0309 |
| C9ZAA4 | Nickel superoxide dismutase | SCAB_29931 (*sodN*) | 0.1509 | 0.0975 | 0.1122 |
| C9Z087 | Alkyl hydroperoxide reductase AhpD | SCAB_8351 | 0.0676 | 0.0501 | 0.0871 |
| C9YYC2 | Stress-induced protein | SCAB_70311 | 0.0257 | 0.0120 | 0.0421 |
| K4QYE6 | Catalase-peroxidase | BN159_1568 (*katG*) | 0.0928 | 0.1531 | 0.1728 |
| A0A1B1AXM1 | Catalase-peroxidase | AVL59_18355 (*katG*) | 0.0457 | 0.0949 | 0.1185 |
| C9YU91 | Non-heme chloroperoxidase | SCAB_4601 (*cpo*) | ND | 0.0340 | 0.1109 |
| A0A1W7CYX9 | Catalase-peroxidase | CAG99_14675 (*katG*) | ND | 0.0444 | 0.0465 |
| A0A117EFM9 | Beta-lactamase | SsS58_05982 (*ampC*) | ND | 0.0387 | ND |
| General function predicted only | | | | | |
| C9Z4H0 | Dehydrogenase | SCAB_73871 | 0.2284 | 0.2408 | 0.2757 |
| C9ZFP0 | O-methyltransferase | SCAB_65281 | 0.2282 | 0.1799 | 0.2202 |
| C9ZED7 | Bac_luciferase domain-containing protein | SCAB_79681 | 0.1352 | 0.0823 | 0.1112 |
| A0A117EGA2 | Limonene 1,2-monooxygenase | SsS58_07272 (*limB_3*) | 0.1101 | 0.0432 | 0.0626 |
| A0A100JVK8 | Anthranilate 1,2-dioxygenase system | SsS58_06931 (*andAa_2*) | 0.0431 | 0.0517 | 0.0631 |
| A0A0L0JPE4 | Anti-sigma regulatory factor | IQ64_44980 | 0.1558 | 0.1687 | 0.1441 |
| C9Z871 | Oxidoreductase | SCAB_13411 | 0.0629 | 0.0642 | 0.0432 |
| C9YTN3 | Hydrolase | SCAB_51411 | 0.0465 | 0.0757 | 0.0537 |
| C9Z258 | N-acetyltransferase domain-containing protein | SCAB_25431 | 0.0907 | 0.0909 | 0.1224 |
| C9ZAA1 | Methyltransferase | SCAB_29911 (*metZ*) | 0.0683 | 0.0574 | 0.0401 |
| C9Z6T5 | F420_oxidored domain-containing protein | SCAB_27741 | 0.0800 | 0.0495 | 0.0473 |
| C9YT02 | Oxidoreductase | SCAB_18931 | 0.0535 | 0.0792 | 0.0695 |
| C9YVB7 | Regulatory ATPase | SCAB_67901 | 0.0324 | 0.0322 | 0.0380 |
| C9ZDK2 | ATP binding protein | SCAB_47231 | 0.1469 | 0.0536 | 0.0751 |
| C9YYQ8 | Oxidoreductase | SCAB_7771 | 0.0530 | 0.0424 | 0.0426 |
| C9ZG65 | Oxidoreductase | SCAB_2951 | 0.0472 | 0.0246 | 0.0215 |
| C9Z8G4 | Secreted serine-type endopeptidase | SCAB_28921 | 0.0334 | 0.0092 | 0.0137 |
| C9ZCZ9 | Hydrolase | SCAB_1601 | ND | 0.0404 | 0.0314 |
| C9Z085 | Oxidoreductase | SCAB_8331 | 0.0416 | 0.0100 | 0.0175 |
| C9Z4B0 | Oxidoreductase | SCAB_58381 | ND | 0.0215 | 0.0386 |
| C9ZCL9 | Secreted tripeptidylaminopeptidase | SCAB_78431 | 0.0449 | 0.0076 | 0.0015 |
| C9Z0G5 | Thioesterase | SCAB_24031 | 0.0076 | 0.0318 | 0.0740 |
| A0A086GHP3 | Peptidase | IQ62_44750 | 0.1224 | 0.1679 | 0.0944 |
| A0A1C6LHC2 | Aminopeptidase N | YUWDRAFT_00059 | 0.0114 | 0.0105 | 0.0169 |
| M3DHC3 | DSBA domain-containing protein | SBD_3460 | 0.0127 | 0.0479 | 0.0174 |
| C9YZV3 | Inhibitor of DNA gyrase | SCAB_71311 | 0.1443 | 0.1245 | 0.1216 |
| C9Z5R3 | DNA-binding protein | SCAB_58741 | 0.0431 | 0.0491 | 0.0804 |
| A0A1M5XKP5 | Type I restriction enzyme M protein | SAMN05444521_3179 | 0.0091 | 0.0136 | 0.0192 |
| A0A0F5VRB1 | ATP-binding protein | TN53_32520 | 0.0112 | 0.0282 | 0.0227 |
| C9ZBW6 | Cytokinin riboside 5'-monophosphate phosphoribohydrolase | SCAB_31221 | 0.0432 | 0.0666 | 0.0349 |
| L7FDE0 | Tat pathway signal sequence domain protein | STRTUCAR8_07223 | 0.1381 | 0.0849 | 0.0959 |
| A0A089Z2W2 | Lipoprotein (General nucleoside transporter) | SGLAU_21035 | 0.1000 | 0.1430 | 0.1547 |
| C9Z982 | Histidine kinase | SCAB_61491 | 0.0777 | 0.0796 | 0.0900 |
| C9ZAB5 | Cyclic-nucleotide-binding protein | SCAB_30041 | 0.1999 | 0.1700 | 0.1881 |
| C9Z230 | Histidine kinase | SCAB_25151 | 0.0170 | 0.0200 | 0.0257 |
| C9Z4L8 | Histidine kinase | SCAB_87071 | 0.0246 | 0.0156 | 0.0211 |
| C9YT07 | TIR domain-containing protein | SCAB_18981 | 0.0286 | 0.0120 | 0.0211 |
| A0A100JQJ1 | Response regulator PleD | SsS58_04257 (*pleD*) | 0.1351 | 0.1274 | 0.1487 |
| C9YZ98 | Two component response regulator | SCAB_38801 | 0.1101 | 0.0786 | 0.0809 |
| C9YZK6 | Two-component system response regulator | SCAB_55341 | 0.0782 | 0.0928 | 0.0747 |
| C9ZAH7 | PPM-type phosphatase domain-containing protein | SCAB_45491 | 0.0537 | 0.0251 | 0.0232 |
| C9ZC02 | Phosphoesterase | SCAB_46371 | 0.0548 | 0.0333 | 0.0442 |
| C9ZF72 | PPM-type phosphatase domain-containing protein | SCAB_33591 | 0.0141 | 0.0208 | 0.0214 |
| C9YZW1 | Uncharacterized protein | SCAB_71391 | 0.0623 | 0.0782 | 0.1080 |
| C9ZA87 | Two-component system response regulator | SCAB_29761 | 0.0582 | 0.0697 | 0.0504 |
| C9YUV1 | Two-component system response regulator | SCAB_35961 | 0.0637 | 0.0348 | 0.0335 |
| C9YYX8 | Calcineurin-like phosphoesterase | SCAB_23201 | 0.0137 | 0.0145 | 0.0119 |
| C9Z1Q3 | Regulatory protein | SCAB_85931 | 0.0211 | 0.0382 | 0.0418 |
| A0A117EC11 | Thioredoxin reductase | SsS58_00543 (*trxB_1*) | 0.0205 | 0.0166 | 0.0153 |
| C9Z0S9 | Two component system response regulator | SCAB_39621 | 0.0760 | 0.0325 | 0.0279 |
| A0A1V9K3X8 | Histidine kinase | B0675_29350 | 0.0155 | 0.0076 | 0.0094 |
| L1KVZ3 | Histidine kinase | STRIP9103_03046 | 0.0346 | 0.0610 | 0.0211 |
| A0A101PCJ3 | Histidine kinase | AQI96_26145 | 0.0182 | 0.0070 | 0.0060 |
| L7ESI1 | Histidine kinase | STRTUCAR8_07988 | 0.0161 | 0.0045 | ND |
| A0A1C6NA13 | Histidine kinase | YWIDRAFT_01395 | ND | 0.0101 | 0.0048 |
| A0A0M9YG60 | Histidine kinase (EC 2.7.13.3) | ADK54_14870 | ND | 0.0236 | 0.0085 |
| C9Z3V0 | Cyclic-nucleotide-binding protein | SCAB_41311 | 0.4334 | 0.3809 | 0.4313 |
| C9Z0H0 | Two-component response regulator | SCAB_24081 | 0.2597 | 0.0958 | 0.1741 |
| C9YZF6 | Two-component system reponse regulator | SCAB_54821 | 0.1789 | 0.1368 | 0.1145 |
| A0A143C616 | DNA-binding response regulator | A4E84_24745 | 0.0383 | 0.0461 | 0.0174 |
| Unknown function | | | | | |
| C9ZEV0 | Uncharacterized protein | SCAB_17531 | 0.0203 | 0.0413 | 0.0484 |
| C9Z8C3 | cellulose-binding protein | SCAB_28521 (*abpS*) | 0.3597 | 0.3667 | 0.4687 |
| C9ZB66 | Uncharacterized protein | SCAB_77921 | 0.1167 | 0.1270 | 0.1549 |
| C9YZV2 | Uncharacterized protein | SCAB_71301 | 0.1027 | 0.1110 | 0.0927 |
| C9YVE9 | Uncharacterized protein | SCAB_68241 | 0.0924 | 0.0908 | 0.1116 |
| M3EQ27 | FHA domain-containing protein | SBD_1273 | 0.1843 | 0.1305 | 0.2144 |
| C9Z393 | regulatory protein | SCAB_86731 | 0.2180 | 0.2922 | 0.1436 |
| C9Z8U9 | Uncharacterized protein | SCAB_45111 | 0.1776 | 0.1087 | 0.1502 |
| C9ZBQ5 | Uncharacterized protein | SCAB_30591 | 0.0973 | 0.1014 | 0.0943 |
| C9ZB81 | Uncharacterized protein | SCAB_78081 | 0.0677 | 0.0564 | 0.0682 |
| C9Z700 | Uncharacterized protein | SCAB_28411 | 0.1696 | 0.1679 | 0.1960 |
| C9ZAJ3 | Uncharacterized protein | SCAB_45661 | 0.1546 | 0.1617 | 0.1847 |
| C9YTI3 | Uncharacterized protein | SCAB_50881 | 0.0274 | 0.0276 | 0.0270 |
| C9YV54 | Uncharacterized protein | SCAB_52131 | 0.1544 | 0.1075 | 0.1047 |
| C9YY57 | Uncharacterized protein | SCAB_54651 | 0.1471 | 0.4437 | 0.2772 |
| C9YYP6 | Uncharacterized protein | SCAB_7641 | 0.2192 | 0.1261 | 0.1210 |
| C9Z2N1 | Uncharacterized protein | SCAB_57151 | 0.1130 | 0.0856 | 0.0816 |
| C9YWC0 | UPF0234 protein SCAB_37371 | SCAB_37371 | 0.2349 | 0.1919 | 0.2697 |
| C9YUE9 | secreted protein | SCAB_5221 | 0.1334 | 0.2514 | 0.2748 |
| C9ZD54 | DNA-binding protein | SCAB_16471 | 0.1225 | 0.1168 | 0.1792 |
| C9ZBU0 | Uncharacterized protein | SCAB_30961 | 0.1238 | 0.1195 | 0.1317 |
| C9Z1N4 | Uncharacterized protein | SCAB_85721 | 0.1422 | 0.1434 | 0.1824 |
| C9Z6Y6 | Uncharacterized protein | SCAB_28271 | 0.1160 | 0.1397 | 0.1520 |
| A0A117EDT7 | BNR/Asp-box repeat protein | SsS58_03198 | 0.1207 | 0.0520 | 0.0560 |
| A0A100JK15 | HTH cro/C1-type domain-containing protein | SsS58_01304 | 0.1042 | 0.1153 | 0.1223 |
| C9Z0G3 | Uncharacterized protein | SCAB_24011 | 0.1013 | 0.0899 | 0.1002 |
| C9ZBP7 | UPF0182 protein SCAB_30501 | SCAB_30501 | 0.0244 | 0.0246 | 0.0247 |
| C9ZFN9 | Uncharacterized protein | SCAB_65271 | 0.2065 | 0.2168 | 0.2394 |
| C9YVG8 | Uncharacterized protein | SCAB_68431 | 0.0894 | 0.1134 | 0.1099 |
| C9ZC26 | membrane protein | SCAB_46621 | 0.1567 | 0.0900 | 0.0647 |
| C9Z0U7 | Uncharacterized protein | SCAB_39811 | 0.0528 | 0.0512 | 0.0698 |
| C9ZGR0 | VWFA domain-containing protein | SCAB_34071 | 0.0657 | 0.1032 | 0.0967 |
| C9Z064 | secreted protein | SCAB_85031 | 0.1368 | 0.2153 | 0.1451 |
| C9ZBQ8 | Uncharacterized protein | SCAB_30621 | 0.0943 | 0.0759 | 0.0975 |
| C9ZC41 | Uncharacterized protein | SCAB_46771 | 0.0564 | 0.0623 | 0.0427 |
| C9ZGE6 | Uncharacterized protein | SCAB_17861 | 0.0387 | 0.0466 | 0.0466 |
| C9YUS2 | Uncharacterized protein | SCAB_20571 | 0.1107 | 0.1199 | 0.1101 |
| C9YVV6 | Uncharacterized protein | SCAB_20801 | 0.0446 | 0.0535 | 0.0623 |
| C9Z382 | exported protein | SCAB_86621 | 0.0762 | 0.0512 | 0.0387 |
| A0A117EFE3 | Tetratricopeptide repeat protein | SsS58_05887 | 0.0072 | 0.0171 | 0.0178 |
| A0A117EB44 | AAA domain-containing protein | a10_08168 | 0.0308 | 0.0198 | 0.0251 |
| C9YZX6 | Uncharacterized protein | SCAB_71561 | 0.1129 | 0.0599 | 0.0911 |
| C9ZBQ7 | Uncharacterized protein | SCAB_30611 | 0.0888 | 0.0413 | 0.0760 |
| C9YTI5 | secreted protein | SCAB_50911 | 0.0762 | 0.0399 | 0.0447 |
| C9Z436 | Uncharacterized protein | SCAB_42191 | 0.0603 | 0.0505 | 0.0444 |
| A0A0L0KZI9 | Uncharacterized protein | IQ64_19465 | 0.0467 | 0.0611 | 0.0319 |
| C9ZFR1 | integral membrane protein | SCAB_65491 | 0.0641 | 0.0737 | 0.0886 |
| C9Z6Z8 | Uncharacterized protein | SCAB_28391 | 0.0838 | 0.0805 | 0.0359 |
| C9YZ07 | Uncharacterized protein | SCAB_23491 | 0.0229 | 0.0242 | 0.0353 |
| C9ZCQ2 | Uncharacterized protein | SCAB_78771 | 0.1601 | 0.1061 | 0.1725 |
| A0A0L0KN23 | Cellulose-binding protein (Chromosome partition protein Smc) | a10_08877 IQ63_02090 (*smc_5*) | 0.2747 | 0.2433 | 0.3739 |
| C9Z1M7 | hydroxylase | SCAB_85651 | 0.0712 | 0.0493 | 0.0771 |
| C9Z8S1 | VWFA domain-containing protein | SCAB_44831 | 0.0206 | 0.0108 | 0.0247 |
| C9Z4C0 | HTH cro/C1-type domain-containing protein | SCAB_58481 | 0.0528 | 0.0542 | 0.1022 |
| C9YX90 | Uncharacterized protein | SCAB_6791 | 0.0302 | 0.0281 | 0.0402 |
| C9ZAJ4 | Uncharacterized protein | SCAB_45671 | 0.0392 | 0.1205 | 0.0691 |
| C9ZFS7 | zf-RING_7 domain-containing protein | SCAB_65651 | 0.0677 | 0.0687 | 0.0889 |
| A0A100JNJ8 | Uncharacterized protein | SsS58_03179 | 0.0278 | 0.0441 | 0.0189 |
| C9YY46 | Uncharacterized protein | SCAB_54541 | 0.1498 | 0.1038 | 0.0837 |
| C9ZCF1 | NB-ARC domain-containing protein | SCAB_63411 | 0.0076 | 0.0090 | 0.0101 |
| C9YTI6 | secreted protein | SCAB_50921 | 0.0582 | 0.0198 | 0.0317 |
| C9Z632 | integral membrane protein | SCAB_74781 | 0.0200 | 0.0325 | 0.0399 |
| C9Z5E4 | Uncharacterized protein | SCAB_42281 | 0.0575 | 0.0695 | 0.0656 |
| C9YZN9 | Uncharacterized protein | SCAB_70661 | 0.0645 | 0.0289 | 0.0657 |
| A0A086GJ66 | zf-RING_7 domain-containing protein | IQ62_41960 | 0.0670 | 0.0715 | 0.0716 |
| C9ZGY8 | Uncharacterized protein | SCAB_49641 | 0.0961 | 0.0934 | 0.0753 |
| C9YZB7 | Uncharacterized protein | SCAB_39001 | 0.0123 | 0.0425 | 0.0315 |
| C9Z2B5 | Acetyltransf_6 domain-containing protein | SCAB_40531 | 0.0192 | 0.0233 | 0.0249 |
| C9Z0J1 | membrane protein | SCAB_24311 | 0.0209 | 0.0565 | 0.0818 |
| C9Z5R2 | Uncharacterized protein | SCAB_58731 | 0.0461 | 0.0759 | 0.0470 |
| C9Z8Q6 | secreted protein | SCAB_44691 | ND | 0.0195 | 0.0133 |
| C9Z0H9 | Uncharacterized protein | SCAB_24171 | 0.0096 | 0.0407 | 0.0149 |
| C9YYX4 | PHB domain-containing protein | SCAB_23161 | ND | 0.0051 | 0.0781 |
| C9Z623 | secreted protein | SCAB_74681 | ND | 0.0040 | 0.0124 |
| A0A0M8WS29 | Uncharacterized protein | ADL04_37195 | 0.0097 | 0.0232 | 0.0278 |
| C9Z081 | Uncharacterized protein | SCAB_8291 | 0.0959 | 0.0727 | 0.0682 |

Analysis was performed after 5 days of growth in MS-S medium (see Materials and methods for details).

*Data are the mean of four biological replicates

^†^RB: Russet Burbank and YG: Yukon Gold

^¶^ND: Not detected
